# Metal-Ion Interactions with Dodecapeptide Fragments of Human Cationic Antimicrobial Protein LL-37 [hCAP(134–170)]

**DOI:** 10.1021/acs.jpcb.2c05200

**Published:** 2022-09-01

**Authors:** Jakub Brzeski, Dariusz Wyrzykowski, Agnieszka Chylewska, Mariusz Makowski, Anna Maria Papini, Joanna Makowska

**Affiliations:** † Faculty of Chemistry, University of Gdańsk, Wita Stwosza 63, 80-308 Gdańsk, Poland; ‡ Department of Chemistry, University of Pittsburgh, Pittsburgh, Pennsylvania 15218, United States; § Interdepartmental Research Unit of Peptide and Protein Chemistry and Biology, Department of Chemistry “Ugo Schiff”, University of Florence, Via della Lastruccia 13, 50019 Sesto Fiorentino, Italy

## Abstract

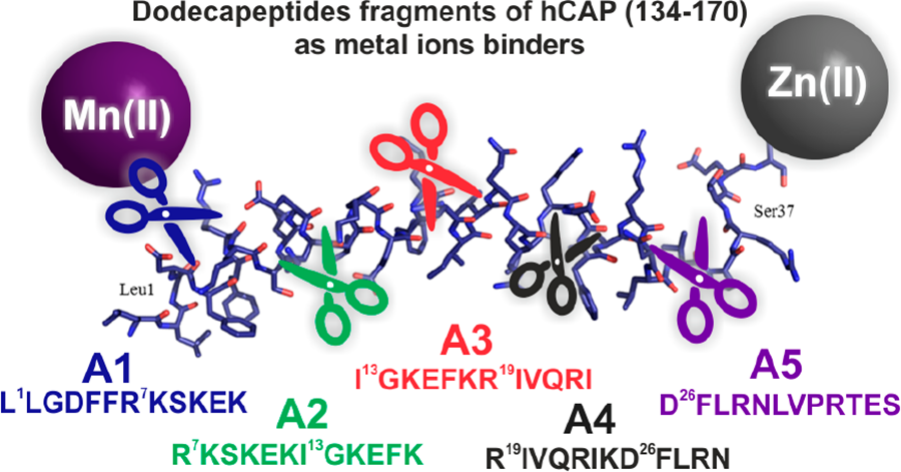

Isothermal titration calorimetry, circular dichroism (CD) techniques, and *in silico* analysis were used to determine potential metal binding sites in human cationic antimicrobial protein (hCAP) corresponding to overlapping the dodecapeptide sequences of hCAP(134–170) referred to as LL-37. The correct antibacterial action of LL-37 is closely related to its established unique structure. Disturbances in the LL-37 structure (e.g., unwanted presence of metal ions) lead to a radical change in its biological functions. Five fragments of the LL-37 [hCAP(134–170)], namely, hCAP(134–145) (**A1**), hCAP(140–151) (**A2**), hCAP(146–157) (**A3**), hCAP(152–163) (**A4**), and hCAP(159–170) (**A5**), were taken into account and their affinity to Mn(II) and Zn(II) ions was rigorously assessed. We prove that only three of the investigated peptides (**A1**, **A2**, and **A5**) are capable of forming thermodynamically stable complexes with metal ions. Additionally, based on density functional theory (DFT) calculations, we propose the most likely coordination modes of metal(II) to peptides as well as discuss the chemical nature of the interactions. Finally, we present the structural features of the strongest binding peptide, hCAP(159–170), responsible for the metal binding. The presented results provide important structural and thermodynamic information to understand the influence of some metal ions on the activity of hCAP(134–170).

## Introduction

Antimicrobial peptides (AMPs) are a group of naturally occurring compounds, and it is believed that these peptides are the oldest element of immunity in living organisms.^1,2^ They are short cationic peptides of up to 100 amino acids^3^ with an α-helical secondary structure and amphiphilic surface properties, which are considered essential for establishing antimicrobial activity.^4,5^ Over 5000 AMPs have been so far identified or synthesized in a wide variety of organisms ranging from prokaryotes (e.g., bacteria) to eukaryotes (e.g., yeasts, fungi, viruses, parasites, protozoa, insects, plants, and animals).^6^ For example, more than 300 different AMPs exist in the skin of frogs, which is a crucial part of the innate immunity against a wide range of microbes including viruses, bacteria, and fungi. Besides their antimicrobial activity, AMPs have anti-inflammatory, antiparasitic, anticancer, antiviral, insecticidal, antibiofilm, wound-healing, and/or chemotactic properties that make them interesting candidates for novel therapeutic strategies.^7−9^

The peptide called LL-37 (LLGDFFRKSKEKIGKEFKRIVQRIKDFLRNLVPRTES) is a fragment of 37 amino acids of the human cationic antimicrobial protein (hCAP), corresponding to residues hCAP(134–170). This is so far the only human cathelicidin that has been described in the literature.^1,10,11^ The mechanism of action of LL-37 is mainly based on the principle of operation of the “carpet” model.^12−17^ Cationic LL-37, as a result of contacts with negatively charged components of the pathogen cell membrane, breaks its continuity by creating pores leading to its death.^18,19^ The correct antibacterial action is closely related to the established unique structure of LL-37. Disturbances in the LL-37 structure^20−22^ lead to a radical change in its biological functions. Metal ions play an important role in the activity of antimicrobial peptides (AMPs) and their interaction with other biomolecules (e.g., proteins or nucleic acids).^23^ Therefore, the maintenance of a precise molecular structure in the presence of metal ions is often of vital importance.^24^ In this situation, the side chains usually act as ligands, and the metal ion interactions with the side chains are designed to constrain the geometry.^25^ Additionally, complexes of biologically active peptides with metal ions may be resistant to enzymatic degradation compared to free ligands. Therefore, studies on the mechanism and specificity of such reactions have attracted the attention of the scientific community for several decades. Usually, peptides binding to metal ions, are contained in the sequence histidine and cysteine residues (known for being good metal anchoring sites) surrounded by residues bearing coordinating side chains, especially aspartate and glutamate residues. Copper ions are known for their good interactions with peptides and many of the rules governing this coordination process are already known. Unfortunately, much less is known about interactions of metals with peptide sequences where no histidine residues are present, especially with zinc or manganese ions, which do not have the same binding features as Cu(II) ions. It is commonly known that zinc is necessary for the proper functioning of many enzymes that are crucial for living processes in prokaryotes and eukaryotes. Since this metal ion is necessary for pathogen virulence and survival, the host binds them, thereby reducing their bioavailability and leading to pathogen impairment and ultimately death.^26,27^ There is considerable evidence that excessive environmental and occupational exposure to Mn(II), particularly when inhaled, attacks the central nervous system, inducing symptoms that resemble Parkinson’s disease, called Parkinsonism or manganism.^28,29^ Additionally, hormonal contraception used nowadays forces people to take supplements rich in Mn(II) ions. Improper intake of these drugs causes a high increase in Mn(II) concentration in blood, which can result in various types of subsequent reactions with enzymes and proteins.

**Figure 1:**
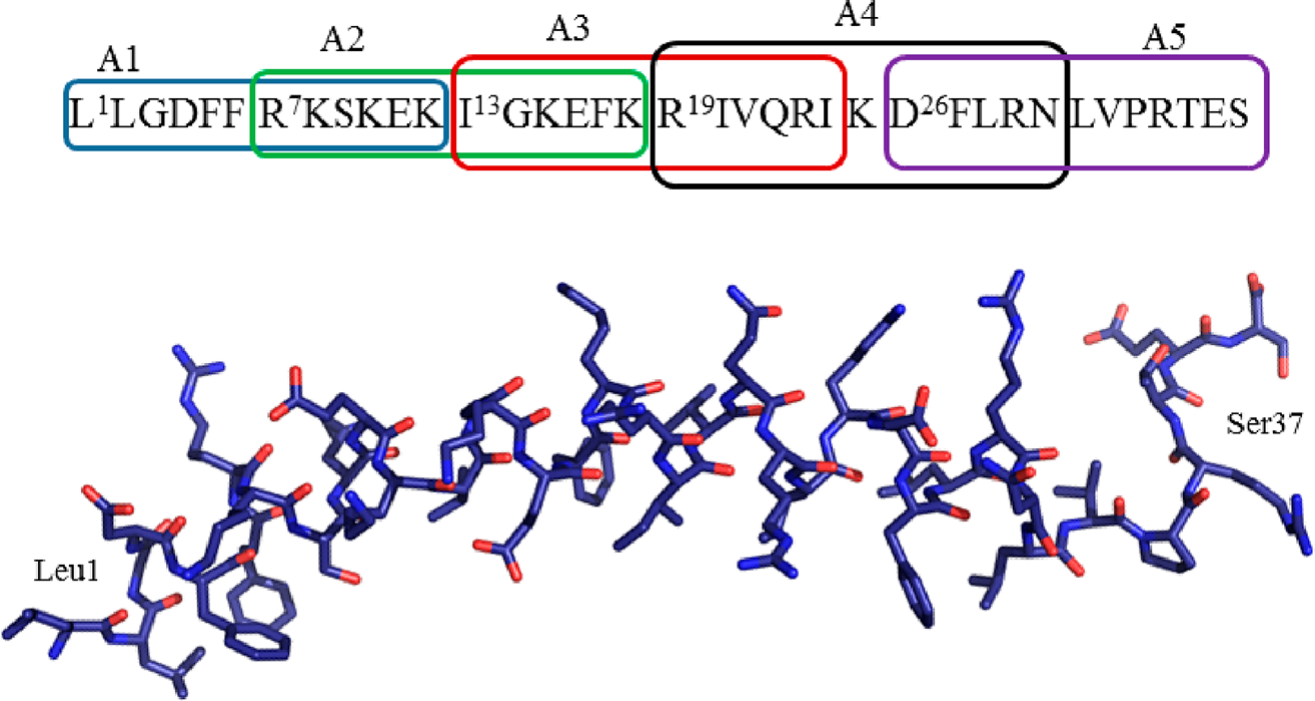
Peptide analogs **A1**–**A5** designed for the present study and correspond to the overlapping shortened dodecapeptide sequences of the peptide called LL37, i.e., hCAP(134–170), and solution structure of human LL-37 in complex with deuterated SDS micelles (PDB id code 2K60).^21,22^

The interactions of hCAP(134–170) (LL-37) with selected metal ions (i.e., Cu(II), Zn(II), and Ni(II)) have previously been investigated.^30^ It has been proven that hCAP(134–170) interacts with Cu(II) and Zn(II) ions, and on this basis, it was found that an excess of both ions in the body may affect the biological activity of this peptide. Since Cu(II) can replace Mn(II) ions as a cofactor of some enzymes, which is related to similar preferences of the interaction of these ions with proteins, we decided to also study the affinity of the peptide hCAP(134–170) toward Mn(II) ions. To get some insight into the putative mode of binding of hCAP(134–170) to metal ions, such as Mn(II) and Zn(II), we selected five fragments of hCAP(134–170) corresponding to the overlapping dodecapeptide sequences hCAP(134–145) (**A1**), hCAP(140–151) (**A2**), hCAP(146–157) (**A3**), hCAP(152–163) (**A4**), and hCAP(159–170) (**A5**) (Figure 1).

In this work, the isothermal titration calorimetry (ITC) technique supported by theoretical calculations was employed for assessing the potential fragments of hCAP(134–170) capable of binding metal ions. The results obtained from the metal–peptide interactions were discussed concerning the structural features of the investigated ligands, which determine their affinity to metal ions. The ITC experiment allowed us to investigate the energy effects of the forming combinations of peptides with metal ions. However, ITC results do not provide knowledge about the type of geometry or the place where the systems bind with metal ions. For this reason, theoretical research has been implemented to determine the geometry of connections in complexes.

## Materials and Methods

To perform the planned tests in the laboratory work, the following chemical reagents were used: sodium salt of 2-(*N*-morpholino) ethanesulfonic acid (Mes, purity ≥ 99), zinc nitrate (V) hexahydrate (Zn(NO_3_)_2_·6H_2_O, purity ≥ 99.99%), manganese(II) bromide (MnBr_2_, purity ≥ 99.99%). The Mes buffer solution was prepared by dissolving *m* = 0.2 g of the substance in 50 mL of doubly deionized water to obtain a solution with a concentration of *c* = 20 mM. To adjust the Mes solution to pH 6.0, a NaOH solution with *c* = 0.0958 M was added.

Peptide analogs **A1**–**A5** designed for the present study and corresponding to the overlapping shortened dodecapeptide sequences of the peptide called LL-37 (i.e., hCAP(134 170)), were synthesized and purified using the procedure described below (“Peptide Synthesis” section).

### Peptide Synthesis

The five dodecapeptides, hCAP(134–145), H-LLGDFFRKSKEK-NH_2_ (**A1**); hCAP(140–151), Ac-RKSKEKIGKEFK-NH_2_ (**A2**); hCAP(146–157), Ac-IGKEFKRIVQRI-NH_2_ (**A3**); hCAP(152–163), Ac-RIVQRIKDFLRN-NH_2_ (**A4**); and hCAP(159–170), Ac-KDFLRNLVPRTES–OH (**A5**), were prepared by microwave-assisted solid phase synthesis (MW-SPPS) with an automated CEM Liberty Blue peptide synthesizer, equipped with a Discovery MW reactor (Matthews, Charlotte, NC). The fully automated MW-SPPS was performed following the three-dimensional orthogonal protection strategy, Fmoc/*t*-Bu.^31,32^ The syntheses started from the Fmoc-Lys(Trt)-Rink amide resin for peptides **A1** and **A2** (loading 0.25 mmol/g), Fmoc-Ile-Rink amide resin for peptide **A3** (loading 0.32 mmol/g,), Fmoc-Asn(Trt)-Rink amide resin for peptide **A4** (loading 0.27 mmol/g), and Fmoc-Ser(tBu)-Rink amide resin for peptide **A5** (loading 0.35 mmol/g).

The Fmoc/tBu MW-SPPS protocol consisted of (1) swelling in DMF for 30 min; (2) double deprotection (20% piperidine in DMF): (a) 15 s, 348.15 K, 155 W, (b) 50 s, 365.15 K, 30 W; (3) washings with DMF (3 × 5 mL); (4) couplings with (a) orthogonally protected amino acids (5 equiv, 0.2 M in DMF), (b) addition of the coupling reagents Oxyma Pure (5 equiv, 1 M in DMF) and DIC (5 equiv, 0.5 M in DMF) in separate bottles; and (5) washings with DMF (3 × 5 mL). Peptide elongation was performed by repeating this general protocol for each amino acid adequately orthogonally protected as follows: Fmoc-Arg(Pbf)–OH, Fmoc-Asp(OtBu)–OH, Fmoc-Asn(Trt)–OH, Fmoc-Gln(Trt)–OH, Fmoc-Glu(OtBu)–OH, Fmoc-Gly-OH, Fmoc-His(Trt)–OH, Fmoc-Ile-OH, Fmoc-Leu-OH, Fmoc-Lys(Boc)–OH, Fmoc-Ser(tBu)–OH, Fmoc-Phe-OH, Fmoc-Pro-OH, Fmoc-Thr(tBu)–OH, Fmoc-Tyr(tBu)–OH, and Fmoc-Val-OH. Both deprotection and coupling reactions were performed in a glass vessel under mechanical mixing and nitrogen bubbling.

Reaction temperatures were monitored by an internal fiberoptic sensor. Both deprotection and coupling reactions were performed in a Teflon vessel under microwave energy and nitrogen bubbling. After Fmoc removal of the last amino acid inserted at the end of the synthesis of peptides **A2**–**A5**, N-terminal acetylation was performed with Ac_2_O and NMM. Peptide **A1** was obtained with the free N-terminal amino function (as represented in LL-37). In all cases, the resin was filtered, washed with DMF (3 × 5 mL) and 2-propanol (3 × 5 mL), and dried under vacuum.

The cleavage of each peptide from the resin, with concomitant deprotection of acid labile amino-acid side-chains, was achieved in 2.5 h at room temperature under magnetic stirring by treatment of the peptide resin with a cocktail of TFA/TIS/H_2_O (10 mL, 95:2.5:2.5). The resin was filtered and rinsed with fresh TFA. The cleavage mixture was precipitated by the addition of ice-cold Et_2_O (20 mL). Precipitated crude peptides **A1**–**A5** were washed with ice-cold Et_2_O (4 × 20 mL) and dried under a vacuum.

Crude peptides **A1**–**A5** were purified by reverse-phase flash chromatography monitored by a UV detector (Biotage Isolera One, Sweden) using a column SNAP Ultra C18 (30 g) [column volume (CV), 45 mL; flow, 25 mL/min; eluents, 0.1% TFA in H_2_O (A) and 0.1% TFA in CH_3_CN (B); gradient, 3 CV of A, 10 CV from 0 to 50% B, 3 CV of B]. After lyophilization, desired dodecapeptides **A1**–**A5** were obtained as white powders (HPLC purity >95%).

Characterization of peptides **A1**–**A5** (Table 1) was performed by ultra-high-performance liquid chromatography RP-UHPLC-MS on a Thermo Scientific Ultimate 3000 (Bremen, Germany), equipped with a diode array detector and a Thermo Scientific-MSQ PLUS, using a C18 Waters (Milford, MA) Acquity CSH column (130 Å, 1.7 μm, 2 × 100 mm; temperature 318.15 K; flow: 0.5 mL/min; eluents: 0.1% TFA in H_2_O (A) and 0.1% TFA in CH_3_CN (B), λ 215 nm). Mass analysis was performed on an MSQ Plus Single Quadrupole Mass Spectrometer equipped with an ElectroSpray Ionization interface module. The UHPLC functions of *A*(*R*_t_) and ESI-MS spectra for peptides studied in this work were shown in Figures S1 and S2, respectively.

**Table 1:** Analytical Data of the Synthetic Peptides

peptide	sequence	UHPLC *R*_t_ (min)	ESI-MS(*m*/*z*)found (calcd)	HPLC purity %	quantity (mg)	yield (%)
hCAP(134–145) (**A1**)	H-LLGDFFRKSKEK-NH_2_	4.033^a^	1467.9 (1466.8) [M + H]^+^	94.0%	44	30%
hCAP(140–151) (**A2**)	Ac-RKSKEKIGKEFK-NH_2_	2.800^a^	1518.9 (1518.9) [M + H]^+^	90.2%	15	10%
hCAP(146–157) (**A3**)	Ac-IGKEFKRIVQRI-NH_2_	4.773^a^	1528.0 (1527.9) [M + H]^+^	93.3%	38	26%
hCAP(152–163) (**A4**)	Ac-RIVQRIKDFLRN-NH_2_	5.013^a^	1599.0 (1598.9) [M + H]^+^	97.7%	37.	23%
hCAP(159–170) (**A5**)	Ac-DFLRNLVPRTES–OH	4.720^a^	1488.8 (1488.0) [M + H]^+^	99.3%	43	29%

a Analytical RP-HPLC gradient: 10–60% B in 5 min.

### Isothermal Titration Calorimetry

All ITC experiments were performed at 298.15 K using the AutoITC isothermal titration calorimeter (MicroCal Inc. GE Healthcare, Northampton, MA). The details of the measuring devices and experimental setup were previously described.^33^ To avoid hydrolysis of metal ions, ITC experiments were carried out in an acidic buffer solution. The reagents (metal ions and peptides) were dissolved directly in 20 mM Mes buffer (pH 6.0). The experiment consisted of injecting 10.02 μL (29 injections, 2 μL for the first injection only, injection duration: 20 s, injection interval: 240 s) of 1 mM peptide solution (**A1**, **A2**, and **A5**) into the reaction cell, which initially contained 0.1 mM solution of the appropriate salt (Mn^2+^ or Zn^2+^). For each experiment, a blank was performed by injecting the titrant solution (peptide) into the cell filled with water only. This blank was subtracted from the corresponding titration to account for the heat of a dilution. The ITC parameters (binding constants *K*_ITC_, the enthalpy change, Δ*H*_ITC_) were obtained by fitting binding isotherms, using nonlinear least-squares procedures, to a model that assumes a single set of identical sites. The stoichiometry of the resulting metal–peptide complexes was fixed to 1:1. The change in free energy of binding (Δ*G*_ITC_) and the entropy change (Δ*S*_ITC_) were calculated directly, using the relationship Δ*G*_ITC_ = Δ*H*_ITC_ – *T*Δ*S*_ITC_ = −*RT* ln *K*_ITC_.

### Circular Dichroism Spectroscopy (CD) Measurements

Circular dichroism (CD) spectra were recorded in 10 mM CACO buffer of pH 6.0 on a Jasco-715 automatic recording spectropolarimeter (Jasco Inc., Easton, MD) at 298.15 K for pure **A5** (c = 1.5 mg/mL) and its complexes with Mn(II) and Zn(II) ions. The spectra were recorded in the 200–700 nm wavelength range in 1 mm quartz cuvettes (the volume of each sample was 0.3 mL), using a sensitivity of five millidegrees and a scan speed of 50 nm min^–1^. Direct CD measurements (θ in millidegrees) were converted to molar ellipticity θ [deg cm^2^ dmol^–1^].

### Theoretical Calculations

#### Molecular Dynamic Simulations with NMR Restraints (Conformational Studies for **A5** Peptide)

The dominant conformation of peptide **A5** present in the solution was determined by using molecular dynamics calculations (MD) with the AMBER 20 program^34^ (AMBER ff20SB force field) at constant volume and temperature (the *NVT* scheme). The simulation was performed in a periodic box of TIP3P water^35^ with the particle-mesh Ewald procedure^36,37^ for long-range electrostatic interactions at 283 K. The total simulation time was 10 ns, and the integration time step was 2 fs. The time-averaged restraint method (TAV)^38,39^ was used to include experimental values for the calculations, with interproton-distance restraints calculated from the intensities of the ROE signals (data not published). The numbers of main interproton-distance restraints were 145 and 47 according to the torsional angles. The interproton distances were restrained with the force constant *k* = 20 kcal/(mol × Å^2^), and the angles were restrainedwith *k* = 2 kcal/(mol × Å^2^). The force constants corresponding to anti-ROE restraints were the same as those corresponding to ROE restraints. A total of 3000 conformations were obtained for the **A5** peptide, and the last 500 were analyzed. The set of the final conformations was clustered by using the MOLMOL program.^40^

#### Quantum Chemical Calculations

The quantum chemical calculations on the 12-amino acid long part of hCAP corresponding to hCAP(159–170) (**A5**) and metal ions Mn(II) and Zn(II) were carried out to qualitatively characterize the metal–peptide interactions. The initial structure of **A5** was taken from the averaged structure of side chains from the NMR experiment. Subsequently, the geometry of **A5** was optimized with the use of the M06 hybrid Minnesota functional created in the group of Truhlar.^41^ The choice of the M06 functional was reasoned by the fact that it has proven itself reliable for both the main group^42^ and transition metal^43^ thermochemistry. The SVP basis set of Ahlrichs and co-workers^44,45^ was selected for all calculations. The SVP basis set itself, although rather modest, was selected to enable the quantum chemical calculations on the system of the significant size that is being dealt with within this paper (210 atoms). Moreover, the SVP basis set was very recently shown to give similar results as its newer version, def-SVP basis^46^ set, regarding the energetics of the transition of l-Ala to its zwitterion.^47^ The harmonic vibrational frequencies corresponding to the stationary points were obtained at the same M06/SVP level of theory to ensure that all of the obtained structures are indeed true minima on the potential energy surface. The five most probable metal ion (Mn(II) and Zn(II)) binding sites were selected based on the analysis of the molecular electrostatic potential map generated for the **A5** peptide fragment. The affinity of the metal ions to a certain binding site was assessed simply by the means of the change in Gibbs free energy of the complexation reaction, Δ*G*_cpx_^298^, corresponding to the following equation:
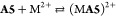
1where M^2+^ represents the metal ions like Zn(II) or Mn(II).

All quantum chemical calculations were performed using the Gaussian16 (Rev. C.01) computational package.^48^

To describe the nature of the bonds formed between **A5** and metal ions, Bader’s QTAIM^49^ (quantum theory of atoms in molecules) analysis has been performed. Additionally, the Dis^50^ (Delocalization Indices) corresponding to the formed bonds have been calculated. The QTAIM is an electron density (ρ) topology analysis. A plethora of useful parameters can be obtained based on the analysis of the electron density distribution. Bearing in mind that the ρ can be treated as a scalar field, one can examine the corresponding gradient vector field, which reveals the direction in which the ρ is increasing the most at a given point. This, in turn, allows for the identification of the so-called critical points (CPs). Due to the subject of the presented study, only the bond critical points (BCPs) corresponding to bonds were scrutinized. Another parameter that is often used in the electron density topology analysis is the Laplacian of the electron density (∇^2^ρ), which indicates the places of local concentration (∇^2^ρ < 0) and depletion (∇^2^ρ > 0) of electron density. Furthermore, the ∇^2^ρ at a given BCP is used to determine the total energy density (*H*_BCP_), according to the following equations:
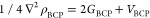
2
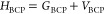
3where *G*_BCP_ and *V*_BCP_ represent the kinetic and potential electron energy density at a given CP respectively.^51,52^

## Results and Discussion

### ITC Measurements

#### Thermodynamic Parameters of the Peptide–Metal Interactions

The diverse analytical methodologies enable the calculation of the stability constant, *K*, and indirectly the free energy of binding, Δ*G* = *f*(*K*). These two parameters provide only general information about the stability of the resulting complexes. The change in the binding enthalpy and the entropy change is necessary for understanding the nature and magnitude of the forces responsible for the mutual affinity of the metal to the ligand.

The ITC technique has been applied for the direct calculation of binding parameters (Δ*H*, and indirectly Δ*S*) of the investigated metal ions with the peptides. However, it should be stressed that the calorimetric investigation of systems in which the metal and the peptide species are involved is not always a simple task. The ITC experimental conditions, namely, pH of the solution, and type and concentration of the buffer used, can affect the affinity of the metal for the peptide, especially for peptides with side chains that contain donor atoms capable to release or uptake protons during the complex formation.^53,54^ Furthermore, hydrolysis of metal ions and competitive reactions of buffer component and metal for a specific peptide, as well as proton competition with the metal for the peptide can also influence the ITC result. For these reasons, the thermodynamic parameters of the metal–peptide binding interactions, obtained directly from the ITC experiments, are so-called conditional dependent parameters. Thus, they can only be compared with those obtained under the same experimental conditions (Tables 2–4).

**Table 2:** Thermodynamic Parameters of **A1** Binding to the Mn^2+^ and Zn^2+^ Ions (Standard Deviation Values in Parentheses) in 20 mM Mes Buffer (pH 6.0) at 298.15 K

ITC parameter	**A1**/Mn^2+^	**A1**/Zn^2+^
log *K*_ITC_	3.58 (±0.02)	3.59 (±0.04)
Δ*G*_ITC_ [kcal mol^–1^]	–4.88 (±0.03)	–4.89 (±0.05)
Δ*H*_ITC_ [kcal mol^–1^]	–4.77 (±0.16)	–4.15 (±0.11)
*T*Δ*S*_ITC_ [kcal mol^–1^]	0.11	0.74

**Table 3:** Thermodynamic Parameters of **A2** Binding to the Mn^2+^and Zn^2+^ Ions (Standard Deviation Values in Parentheses) in 20 mM Mes Buffer (pH 6.0) at 298.15 K

ITC parameter	**A2/**Mn^2+^	**A2**/Zn^2+^
log *K*_ITC_	3.66 (±0.04)	3.68 (±0.04)
Δ*G*_ITC_ [kcal mol^–1^]	–5.00 (±0.05)	–5.02 (±0.05)
Δ*H*_ITC_ [kcal mol^–1^]	–3.89 (±0.18)	–3.47 (±0.16)
*T*Δ*S*_ITC_ [kcal mol^–1^]	1.11	1.155

**Table 4:** Thermodynamic Parameters of **A5** Binding to the Mn^2+^and Zn^2+^ Ions (Standard Deviation Values in Parentheses) in 20 mM Mes Buffer (pH 6.0) at 298.15 K

ITC parameter	**A5**/Mn^2+^	**A5**/Zn^2+^
log *K*_ITC_	3.89 (±0.02)	3.86 (±0.03)
Δ*G*_ITC_ [kcal mol^–1^]	–5.31 (±0.03)	–5.27 (±0.05)
Δ*H*_ITC_ [kcal mol^–1^]	–3.37 (±0.08)	–2.87 (±0.11)
*T*Δ*S*_ITC_ [kcal mol^–1^]	1.94	2.40

The maintenance of the experimental conditions for all the systems under study enabled us to draw some general conclusions regarding the investigated interactions as well as to compare the ITC data with previously published results for the 37 amino acid peptide fragment of hCAP corresponding to the hCAP(134–170) ligand.

The ITC data revealed that the thermodynamic stability of the investigated metal complexes is comparable to each other in the range of the experimental errors (Tables 2–4). However, it is worth emphasizing that stronger metal–ligand interactions have been observed for Zn^2+^ ions with LL-37 (log *K*_ITC_ = 5.19 for LL-37/Zn^2+^).^30^ Furthermore, it is also worth noticing that in contrast to the LL-37/Zn^2+^ complex the formation of complexes under study is accompanied by the release of heat. The thermodynamic parameters revealed that the formation of the investigated metal–peptide complexes is an enthalpy-driven process (|Δ*H*| > |*T*Δ*S*|) (Tables 2–4). In contrast, for the **A5**/Zn^2+^ complex, the entropic contribution is also pronounced. The binding enthalpy has been found to depend on the peptide sequence, and it decreases in the order **A1** > **A2** > **A5** for the given metal ion. Simultaneously, the increase in entropy change is observed in the same direction (**A1** > **A2** > **A5**) and is higher for interactions with Zn(II) than Mn(II). Thus, different types of binding forces seem to be involved in the interactions of metal ions with dodecapeptides **A1**, **A2**, and **A5** but also with the longer sequence LL-37.

### CD Results

**Figure 2:**
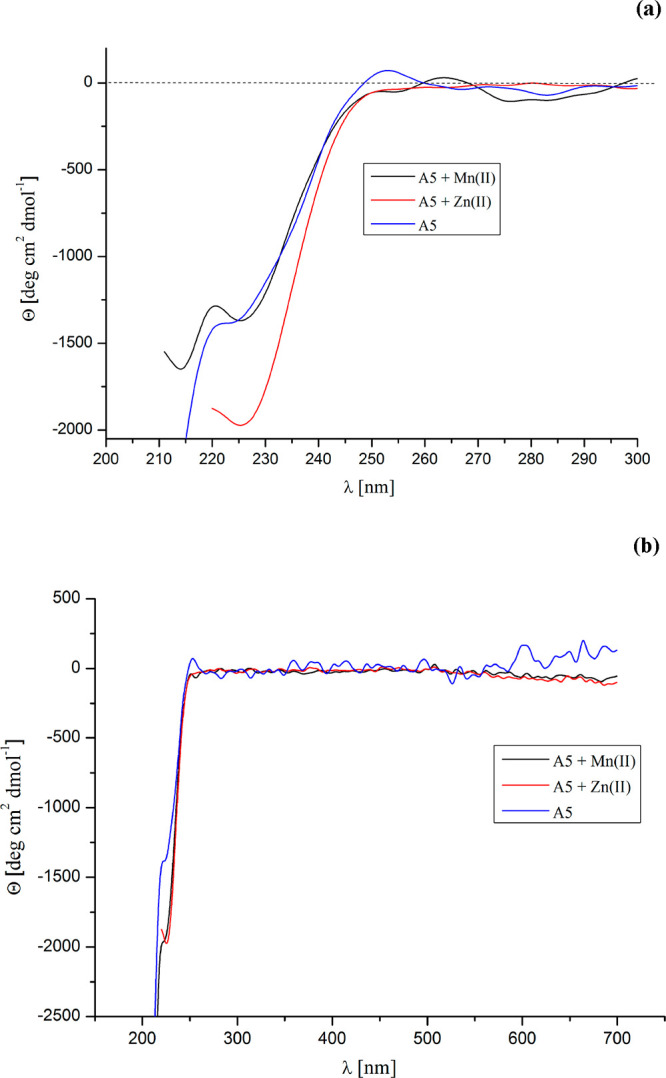
(a) Fragment of CD spectra recorded in the region 200–300 nm for **A5** peptide (blue line) at *T* = 298.15K as well as their manganese(II) (black line) and zinc(II) (red line) complexes. (b) CD spectra recorded for **A5** peptide (blue line) at *T* = 298.15K as well as their manganese(II) (black line) and zinc(II) (red line) complexes.

The CD curve in the region 220–230 nm indicates that **A5** is mostly unordered in the solutions (blue line, see Figure 2a,b). Some changes in the conformation of the peptide were detected after the complexation process. Both Zn(II) and Mn(II) resulted in different types of changes in secondary structure, suggesting that these metal ions provide different environments for the peptide to assume unique secondary structures.^55,56^ (see Figure 2a). Since for the Mn(II) and Zn(II) ions the d–d transitions are not expected in the visible region, the CD curves in 400–700 nm for the complexes under study showed no changes in comparison with the curve estimated for pure peptide **A5** (Figure2b).

#### MD Calculations

**Figure 3:**
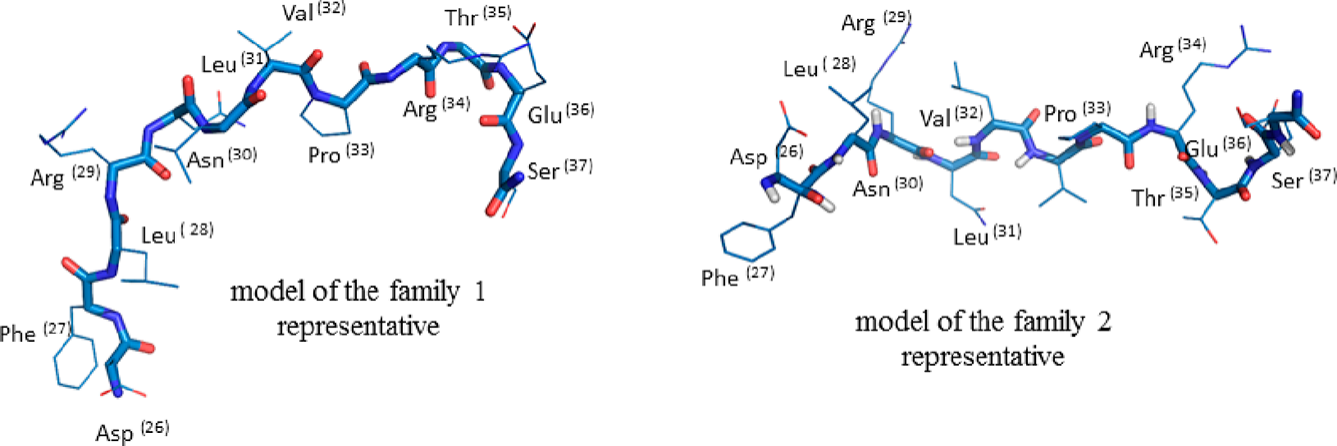
Models of the representatives of the main families for the fragment of human cationic antimicrobial protein hCAP(159–170) (**A5**) clustered by using the hierarchical minimal spanning tree method.^56^ The rms deviation cutoff of 2.1 Å for **A5** over the Leu(28)–Thr(35) residues were used for the clustering. The backbone is shown in stick representation.

The representatives of the two most populated families of conformations of the dodecapeptide hCAP(159–170) (**A5**), obtained by MD simulations with time-averaged constraints derived from NMR measurements and clustered by using the MOLMOL program,^40^ are shown in Figure 3. The representative of the most populated family of peptide **A5** (family 1, populated by 283 conformations) has a characteristic bend at the N-terminal part of the sequence in contrast to the representative of family 2 (populated by 183 conformations), which consists of conformations characterized by a more stretched shape. In both cases, the central part of the main peptide chain in the section (−Asn30–Arg34−) seems to be more rigid while the ends are flexible, especially in family 2. These determined conformations were then used as the starting conformations of peptide **A5** to study interactions with Mn(II) and Zn(II) ions by using the DFT method.

#### DFT Calculations

##### Equilibrium Structures of (M**A5**)^2+^ Complexes

**Figure 4:**
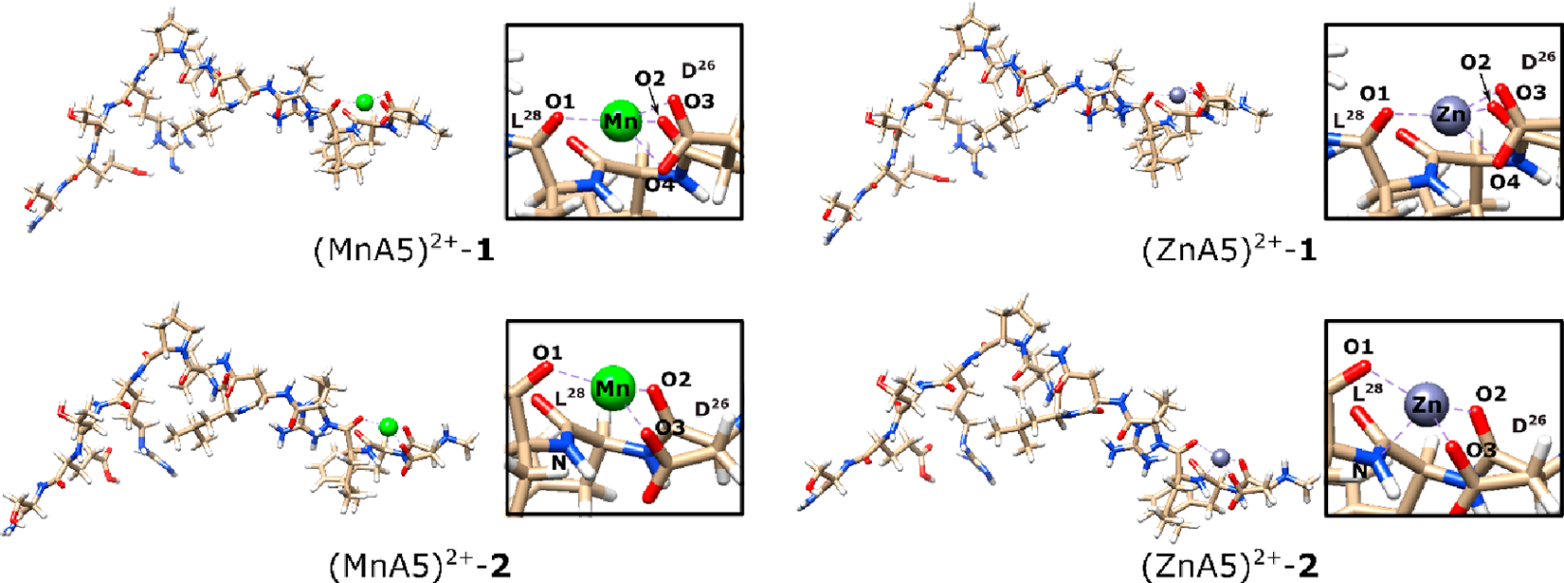
Two of the most thermodynamically favorable M(II) docking conformations.

**Figure 5:**
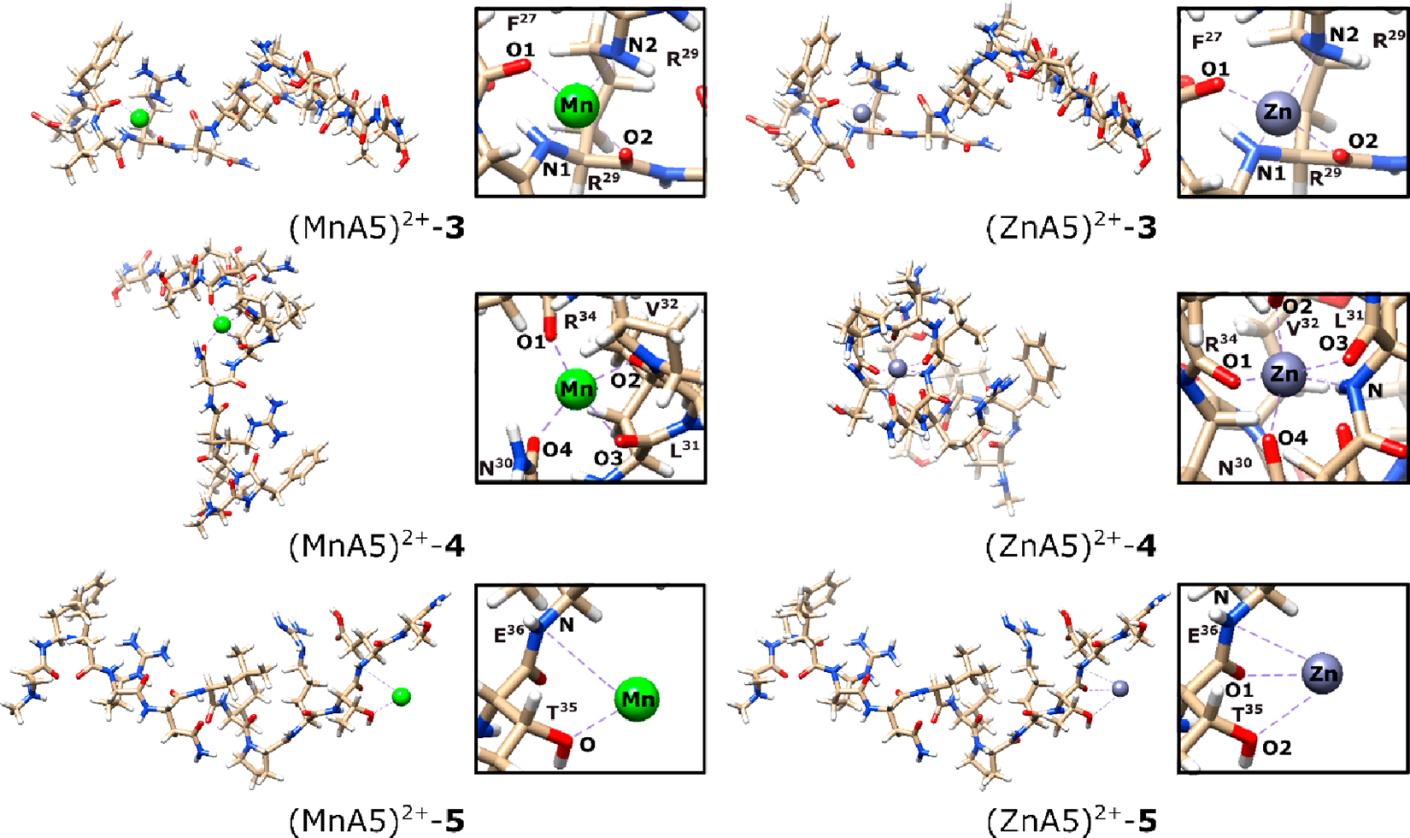
Three remaining M(II) binding sites that are analyzed in this paper.

The metal ion–**A5** binding modes were predicted using quantum chemical calculations. Five binding modes were selected for each metal ion based on their relative Gibbs free energies. The corresponding Δ*G*_cpx_^298^ values are collected in Table 5, whereas the corresponding binding modes are visualized in Figures 4 and 5. It is apparent from Table 5 that dodecapeptide **A5** exhibits a significantly higher affinity toward Zn(II) ions than it does toward Mn(II), regardless of the binding mode considered. The lowest difference in affinity is expected for the strongest complexes, namely, (Mn**A5**)^2+^–1 and (Zn**A5**)^2+^–1). The aforementioned gap constitutes an ample margin of 42.3 kcal/mol.

**Table 5:** Values of the Gibbs Free Energy of Complexation (Δ*G*_cpx_^298^) Corresponding to Studied (M**A5**)^2+^ Systems

system	Δ*G*_cpx_^298^ (kcal/mol)	system	Δ*G*_cpx_^298^ (kcal/mol)
(Mn**A5**)^2+^–1	**–342.3**	(Zn**A5**)^2+^–1	**–384.6**
(Mn**A5**)^2+^–2	–333.4	(Zn**A5**)^2+^–2	–379.0
(Mn**A5**)^2+^–3	–290.4	(Zn**A5**)^2+^–3	–341.4
(Mn**A5**)^2+^–4	–278.1	(Zn**A5**)^2+^–4	–349.7
(Mn**A5**)^2+^–5	–242.1	(Zn**A5**)^2+^–5	–337.5

As illustrated in Figure 4, the two isomers that are the most thermodynamically favorable correspond to the same binding site and differ in the formed bonding pattern.

In the case of (M**A5**)^2+^–1, (where M denotes the metal ion: Mn(II) or Zn(II), respectively), four M–O bonds are formed, whereas in (M**A5**)^2+^–2 complexes three M–O bonds and one M–N bond are formed. Three bond donor atoms remain unchanged in every example. All mentioned bonds are donated between M^2+^ and O atoms of the carboxyl group of D(26), the amide group of D(26), and the amide group of L(28). Hence, the difference in binding is based on the nature of the fourth bond, that is, M–O (carboxyl group of D(26) in the case of (M**A5**)^2+^–1) or M–N (amide group of L(28) in the case of (M**A5**)^2+^–2). The differences in the Δ*G*_cpx_^298^ value between (M**A5**)^2+^–2 and (M**A5**)^2+^–1 are equal to 8.9 and 5.6 kcal/mol for Mn(II) and Zn(II) complexes, respectively. The fact that (M**A5**)^2+^–1 complexes are favored thermodynamically over (M**A5**)^2+^–2 arises from two factors: (i) Both metal ions have a higher affinity toward the more electronegative atoms, especially when those are part of an ionized moiety (such as COO^–^ group). (ii) The discussed O-atom is more sterically accessible than the N-atom. Additionally, in (M**A5**)^2+^–1 the metal ions are bonded in a tetrahedral manner, whereas in the case of (M**A5**)^2+^–2, the tetrahedron is significantly distorted.

As visualized in Figure 5, the second most favorable binding site ((M**A5**)^2+^–3) is located between the amino acids F(27) and R(29). Although it is only the second most preferred site, the corresponding Gibbs free energies relative to that of (M**A5**)^2+^–1 are significantly higher, by 51.9 and 43.2 kcal/mol for Mn(II) and Zn(II) bonded complexes, respectively.

The considerable difference in relative energies does not arise only from the differences in the energetics of formed bonds. The binding of metal ions to the three binding sites enforces a less energetically favorable arrangement of the **A5** sequence itself. For example, this binding imposes that the side chains of phenylalanine and leucine are only 2.497 Å apart (H–H distance). In the structure of said complexes, the metal ions are bonded by two M–N and two M–O type bonds. Three of the formed bonds come from one amino acid (i.e., arginine R(29)): One oxygen atom and one nitrogen donor atom reside in the backbone; the remaining bond comes from the nitrogen atom of the guanidine group of the R(29) side chain. Additionally, the complex is stabilized by the oxygen atom from the carbonyl group of phenylalanine F(27). In the next reported binding site ((M**A5**)^2+^–4), both metal ions are bonded with four oxygen atoms; in the case of Zn(II) complexes, there is an additional bond with the nitrogen atom (i.e., L(31)). All of the formed bonds are a part of carbonyl groups of the backbone. The amino acids involved in the bonding are N(30), L(31), V(32), and R(34). Due to the high relative Gibbs energies (64.2 and 34.9 kcal/mol for Mn(II) and Zn(II), respectively), these complexes are not expected to dominate the population under standard conditions. It is worth noting here that the (Zn**A5**)^2+^–4 complex is described by a Δ*G*_cpx_^298^ value lower than that of (Zn**A5**)^2+^–3, by 8.3 kcal/mol. This indicates a lower affinity of Zn(II) toward the latter. This is due to the bond network of (Zn**A5**)^2+^–4 being advantageous over that of (Zn**A5**)^2+^–3. In the last described binding sites ((M**A5**)^2+^–5), Mn(II) and Zn(II) are rather weakly tethered to the amino acids T(35) and E(36). In the case of both Mn(II) and Zn(II), the nitrogen atom (amide group) of E(36) and the oxygen atom (side-chain) of T(35) are involved in the formation of the complex. Interestingly, the (Zn**A5**)^2+^–5 complex is bound by an additional 2.813 Å Zn–O2 bond from the oxygen atom of the amide group of threonine. The analogous distance for the case of (Mn**A5**)^2+^–5 was found to be equal to 4.117 Å. The QTIAM analysis has also proved the nonexistence of the Mn–O2 interaction in this complex. The (M**A5**)^2+^–5 complexes are expected to be highly labile. Due to the length and electron-density related parameters (see Table 5), only one of each bond formed between the metal ion and peptide in (Mn**A5**)^2+^–5 and (Zn**A5**)^2+^–5 may be regarded (Mn–O for (Mn**A5**)^2+^–5 and in Zn–O2 for (Zn**A5**)^2+^–5), the remaining bonds should be regarded as weaker, electrostatic-based interactions. This is because within their structure the metal ions are neither entangled in any significant net of bonds nor are they surrounded by them, which was the case for the remaining complexes. The lability of those systems is manifested by the corresponding values of Δ*G*_cpx_^298^. Final electronic energies (in Hartree, *E*), Gibbs free energies (in Hartree, *G*), enthalpies (in Hartree, *H*), and atom coordinates (in Å) calculated at the M06/SVP level (gas phase) for all 10 Mn/Zn**A5**)^2+^ metal-ion complexes were also shown in Table S1. Here it should be emphasized that MD simulations were performed on the hCAP(159–170) (**A5**) dodecapeptide only, while the metal ion/peptide interactions were studied with DFT.

#### Analysis of Topology of Electron Density

The bonds formed between dodecapeptide **A5** and the studied metal ions were investigated using the QTAIM topology of the electron density analysis. This analysis allows for the description of the interaction based on the values of certain parameters (e.g., ∇^2^ρ_BCP_ or |*V*_BCP_|/*G*_BCP_) at the corresponding BCP. The Laplacian of the electron density (∇^2^ρ_BCP_), which mathematically is a trace of the Hessian matrix, allows for the detection of the valence electrons. This would be impossible with the ρ itself since it is dominated by the core electrons.^57,58^ Based on the value of ∇^2^ρ_BCP_ corresponding to a given BCP, one can assign the nature of the interaction it describes, namely, negative and positive values correspond to open- and closed-shell interactions, respectively. As apparent from Table 6, all detected M^2+^–**A5** interactions are described by a positive value of ∇^2^ρ_BCP_, which is in line with the coordinative nature of the interactions under study.

**Table 6:** Topological and Energetic Parameters at the BCPs Corresponding to the Bonds Formed in (M**A5**)^2+^ Systems^a^

bond type	bond length	ρ_BCP_	∇^2^ρ_BCP_	*V*_BCP_	*G*_BCP_	*H*_BCP_	*E*_BCP_	**|***V*_BCP_**|/***G*_BCP_	*H*_BCP_**/**ρ_BCP_
(Mn**A5**)^2+^–1									
Mn–O1	2.000	0.07563	0.51826	–76.3	78.8	2.5	38.1	0.97	0.053
Mn–O2	2.127	0.05426	0.33345	–45.1	48.7	3.6	22.6	0.93	0.105
Mn–O3	2.087	0.06130	0.35770	–50.9	53.5	2.6	25.5	0.95	0.068
Mn–O4	2.110	0.06411	0.38519	–54.9	57.7	2.7	27.5	0.95	0.068
(Zn**A5**)^2+^–1									
Zn–O1	1.906	0.08900	0.60972	–92.7	94.2	1.5	46.4	0.98	0.026
Zn–O2	2.082	0.05707	0.30126	–48.9	48.1	–0.8	24.4	1.02	–0.023
Zn–O3	2.012	0.06474	0.33735	–55.8	54.3	–1.4	27.9	1.03	–0.035
Zn–O4	2.060	0.07162	0.40186	–64.2	63.6	–0.6	32.1	1.01	–0.013
(Mn**A5**)^2+^–2									
Mn–O1	2.010	0.07553	0.50020	–74.2	76.3	2.2	37.1	0.97	0.045
Mn–O2	2.056	0.06438	0.42185	–59.1	62.7	3.5	29.6	0.94	0.087
Mn–O3	1.931	0.09233	0.61304	–99.5	97.8	–1.7	49.8	1.02	–0.029
Mn–N	2.480	0.03211	0.11404	–18.1	18.0	–0.1	9.1	1.01	–0.006
(Zn**A5**)^2+^–2									
Zn–O1	1.922	0.08713	0.57585	–88.3	89.3	1.0	44.2	0.99	0.018
Zn–O2	1.993	0.07014	0.42513	–65.3	66.0	0.7	32.7	0.99	0.015
Zn–O3	1.872	0.09759	0.66573	–104.9	104.7	–0.3	52.5	1.00	–0.004
Zn–N	2.352	0.04069	0.12056	–27.9	23.4	–4.5	13.9	1.19	–0.175
(Mn**A5**)^2+^–3									
Mn–O1	1.940	0.09248	0.60330	–98.2	96.4	–1.8	49.1	1.02	–0.030
Mn–O2	2.031	0.07248	0.45964	–68.0	70.1	2.1	34.0	0.97	0.045
Mn–N1	2.215	0.05888	0.25836	–42.9	41.7	–1.2	21.4	1.03	–0.032
Mn–N2	2.261	0.05157	0.23080	–36.1	36.2	0.0	18.1	1.00	0.001
(Zn**A5**)^2+^–3									
Zn–O1	1.883	0.09704	0.64896	–102.4	102.1	–0.3	51.2	1.00	–0.005
Zn–O2	1.985	0.07608	0.44581	–70.1	70.0	0.0	35.0	1.00	–0.001
Zn–N1	2.084	0.07288	0.31812	–62.0	56.0	–6.1	31.0	1.11	–0.133
Zn–N2	2.118	0.06601	0.28718	–54.8	49.9	–4.9	27.4	1.10	–0.118
(Mn**A5**)^2+^–4									
Mn–O1	2.016	0.06905	0.48700	–68.6	72.5	3.9	34.3	0.95	0.090
Mn–O2	2.117	0.05576	0.34243	–46.9	50.3	3.4	23.5	0.93	0.097
Mn–O3	2.173	0.04954	0.27881	–38.2	41.0	2.8	19.1	0.93	0.089
Mn–O4	2.117	0.05522	0.34624	–46.9	50.6	3.7	23.5	0.93	0.107
(Zn**A5**)^2+^–4									
Zn–O1	1.972	0.03631	0.47249	–70.5	72.3	1.8	35.3	0.98	0.079
Zn–O2	1.987	0.07114	0.43704	–67.1	67.8	0.7	33.6	0.99	0.016
Zn–O3	2.198	0.04658	0.18602	–35.5	32.3	–3.1	17.7	1.10	–0.108
Zn–O4	1.989	0.06953	0.44266	–66.1	67.8	1.7	33.1	0.98	0.038
Zn–N	2.168	0.05950	0.23527	–46.9	41.9	–5.0	23.4	1.12	–0.133
(Mn**A5**)^2+^–5									
Mn–O	2.394	0.03088	0.13442	–19.5	20.3	0.8	9.8	0.96	0.040
Mn–N	3.680	0.00560	0.01018	–1.5	1.6	0.0	0.8	0.98	0.009
(Zn**A5**)^2+^–5									
Zn–O1	3.840	0.00764	0.01825	–1.9	2.4	0.5	1.0	0.81	0.097
Zn–O2	2.813	0.01539	0.04081	–6.9	6.6	–0.3	3.4	1.04	–0.026
Zn–N	3.350	0.00789	0.01582	–2.4	2.5	0.0	1.2	0.99	0.006

a Individual parameters are given in the following units: bond lengths in Å; ρ_BCP_ in e Å^–3^; ∇^2^ρ_BCP_ in e Å^–5^; *V*_BCP_, *G*_BCP_, *H*_BCP_, and *E*_BCP_ in kcal mol^–1^ Å^–3^; *H*_BCP_/ρ_BCP_ in [a.u.].

The open-/closed-shell classification has been further improved by Binachi et al.,^59^ who have proposed the classifications of the interactions based on the |*V*_BCP_|/*G*_BCP_ ratio. According to this approach, a ratio of |*V*_BCP_|/*G*_BCP_ higher than 2 describes a shared-shell region of covalent bonds. In contrast, a |*V*_BCP_|/*G*_BCP_ ratio value lower than 1 is related to the closed-shell region of van der Waals interactions and ionic bonds. Finally, an intermediate ratio value (1 < |*V*_BCP_|/*G*_BCP_ < 2) corresponds to the interactions and ionic bonds of weak covalence degree.^36,60^ All but one of all ion–peptide bonds observed in this paper are characterized by a value of |*V*_BCP_|/*G*_BCP_ in the range of 0.93–1.19. This indicates a similar nature of the resulting bonds, which are characterized by a low covalent degree. One mentioned outlier from this trend is the Zn–O1 bond from (Zn**A5**)^2+^–5, for which the |*V*_BCP_|/*G*_BCP_ ratio was calculated to be equal to 0.81 only. This indicates the rather electrostatic nature of the interaction, which is consistent with the significant Zn–O1 bond length of 3.840 Å (the longest bond reported in this paper). Overall, the bonds formed by Zn(II) seem to be described by a higher |*V*_BCP_|/*G*_BCP_ ratio, which indicates that the bonds formed by them are more covalent (or less ionic) than their Mn(II) counterparts. This concurs well with the thermodynamic stability trends observed for the corresponding complexes.

The degree of covalency of a given bond can be assessed based on the *H*_BCP_/ρ_BCP_ value. Espinosa et al.^61^ introduced the concept of bond degree (BD = *H*_BCP_/ρ_BCP_). According to their theory, in the regions where |*V*_BCP_|/*G*_BCP_ < 1, *H*_BCP_/ρ_BCP_ is positive, and the larger the *H*_BCP_/ρ_BCP_ value, the more closed and weaker the noncovalent interaction. The *H*_BCP_/ρ_BCP_ ratio takes negative values and measures the covalency in the regions where the |*V*_BCP_|/*G*_BCP_ > 1. Therefore, the higher the *H*_BCP_/ρ_BCP_ magnitude, the more covalent the interaction. The values of the *H*_BCP_/ρ_BCP_ ratio (together with |*V*_BCP_|/*G*_BCP_) corresponding to the bonds formed between the studied metal ions and **A5** explain why Zn(II) complexes are bonded more tightly than Mn(II) ones. In ITC experiments, the more covalent the contribution to the bonding of metal–ligand, the lower Δ*H*. For example, in the case of the most thermodynamically favorable (M**A5**)^2+^–1 complexes, three out of four bonds formed by Zn(II) are described by a negative value of *H*_BCP_/ρ_BCP_, whereas in the case of Mn(II)-bonded complexes there are no such bonds.

Last, the values of the bond energies were estimated according to the approach proposed by Espinosa:^61,62^
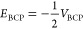
4This equation, although initially used only for the description of hydrogen bonds, now has its applicability extended.^63^ All bond energies span a wide 0.8–52.5 kcal/mol range. Once again, the bonds formed by Zn(II) turned out to be stronger than their Mn(II) counterparts. The average difference was found to be equal to 3.9 kcal/mol.

## Conclusions

The ITC results revealed that under specific experimental conditions (pH 6, 10 mM Caco buffer) only hCAP(134–145) (**A1**), hCAP(140–151) (**A2**), and h CAP(159–170) (**A5**) dodecapeptides form stable complexes of a stoichiometry 1:1 (metal to ligand) with Zn(II) and Mn(II) ions. The formation of the investigated complexes is an enthalpy-driven process. The differences in binding enthalpies of the resulting complexes reflect a different coordination mode of the metal ions as well as the type of donor atoms engaged in the interactions. It is also interesting to note that calorimetric experiments have not provided evidence for the affinity of the investigated metal ions toward the peptides hCAP(146–157) (**A3**) and hCAP(152–163) (**A4**) (Figure S3).

The values of the *H*_BCP_/ρ_BCP_ ratio (together with |*V*_BCP_|/*G*_BCP_) corresponding to the bonds formed between the studied metal ions and **A5** showed that Zn(II) complexes are bonded more tightly than Mn(II) ones, namely, most of the bonds formed by Zn(II) exhibit more covalent character than their Mn(II) counterparts.

Because Zn(II) and Mn(II) ions have an affinity for **A1**, **A2**, and **A5** peptides, it can be presumed that the excess of Zn(II) and Mn(II) ions in the human body may affect the biological stability of the entire fragment hCAP(134–170). Binding to human cathelicidin may make it difficult for this molecule to attach to the cell membrane of pathogens and thus affect the microbial activity of this peptide. As previously mentioned, it should be noted that when peptides with relevant functions in the body form complexes with metal ions, they may be resistant to enzymatic degradation compared to free ligands; thus, LL-37 may have impaired functions when interacting in a nonstandard manner, such as with metal ions.

## Supporting Information Available

UHPLC as functions of *A*(*R*_t_) and ESI-MS spectra for five LL-37 peptide fragments studied in this work; calorimetric titration isotherms of the binding interactions of Zn(II) and Mn(II) metal ions with **A3** and **A4**; final electronic energies (in Hartree, *E*), Gibbs free energies (in Hartree, *G*), enthalpies (in Hartree, *H*), and atom coordinates (in Å) calculated at the M06/SVP level (PDF)
