# Supplementary material for: Metal-Ion Interactions with Dodecapeptide Fragments of Human Cationic Antimicrobial Protein LL-37 [hCAP(134–170)]
Source: J Phys Chem B. 2022 Sep 1;126(36):6911–21. doi: 10.1021/acs.jpcb.2c05200 (PMC9483913; doi:10.1021/acs.jpcb.2c05200)
Supplement: Supplementary file 1 — jp2c05200_si_001.pdf [file jp2c05200_si_001.pdf]

Supplementary Information

Metal-Ions Interactions with Dodecapeptide Fragments of Human Cationic Antimicrobial Protein LL-37 - [hCAP(134-170)]

Jakub Brzeski[1,2], Dariusz Wyrzykowski[1], Agnieszka Chylewska[1], Mariusz Makowski[1], Anna Maria Papini[3], Joanna Makowska[1]*

[1] *Faculty of Chemistry, University of Gdańsk, Wita Stwosza 63, 80-308 Gdańsk, Poland*

[2] *Department of Chemistry, University of Pittsburgh, Pittsburgh, PA 15218, USA*

[3] *Interdepartmental Research Unit of Peptide and Protein Chemistry and Biology, Department of Chemistry "Ugo Schiff", University of Florence, Via della Lastruccia 13, 50019 Sesto Fiorentino, Italy*

Keywords: hCAP fragment, LL-37; Mn(II) complexes; Zn(II) complexes; coordination properties; isothermal titration calorimetry; DFT

***Corresponding author**: e-mail: joanna.makowska@ug.edu.pl

Figure S1. UHPLC spectra for five LL-37 peptide fragments studied in this work as functions of A(R_t).

a) hCAP (134-145) (**A1**)

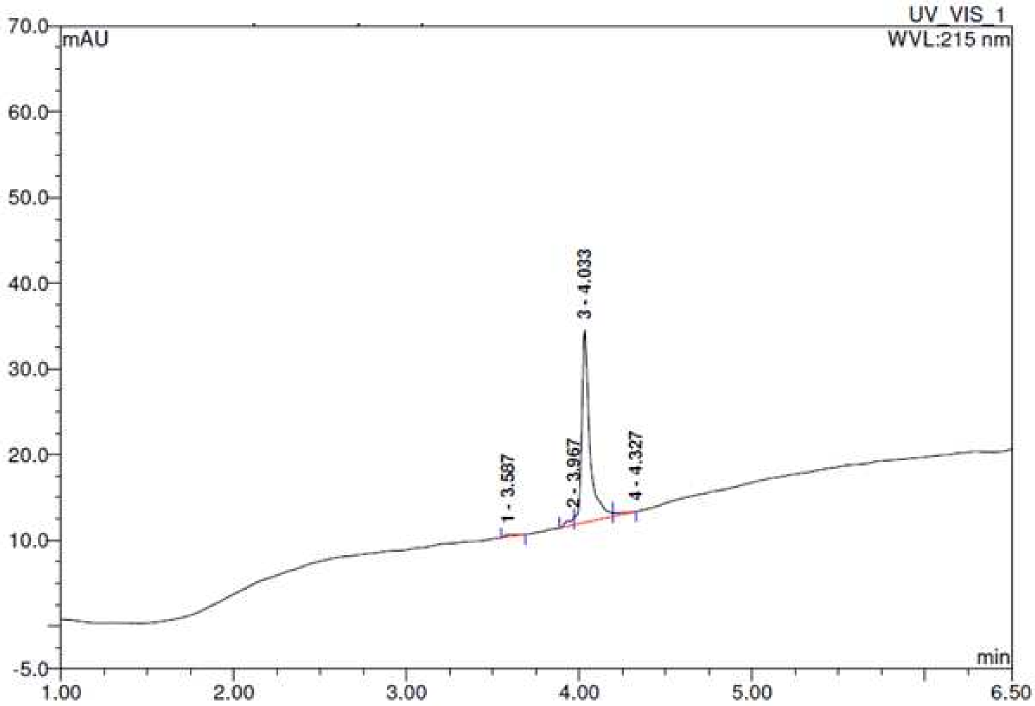

b) hCAP (140-151) (**A2**)

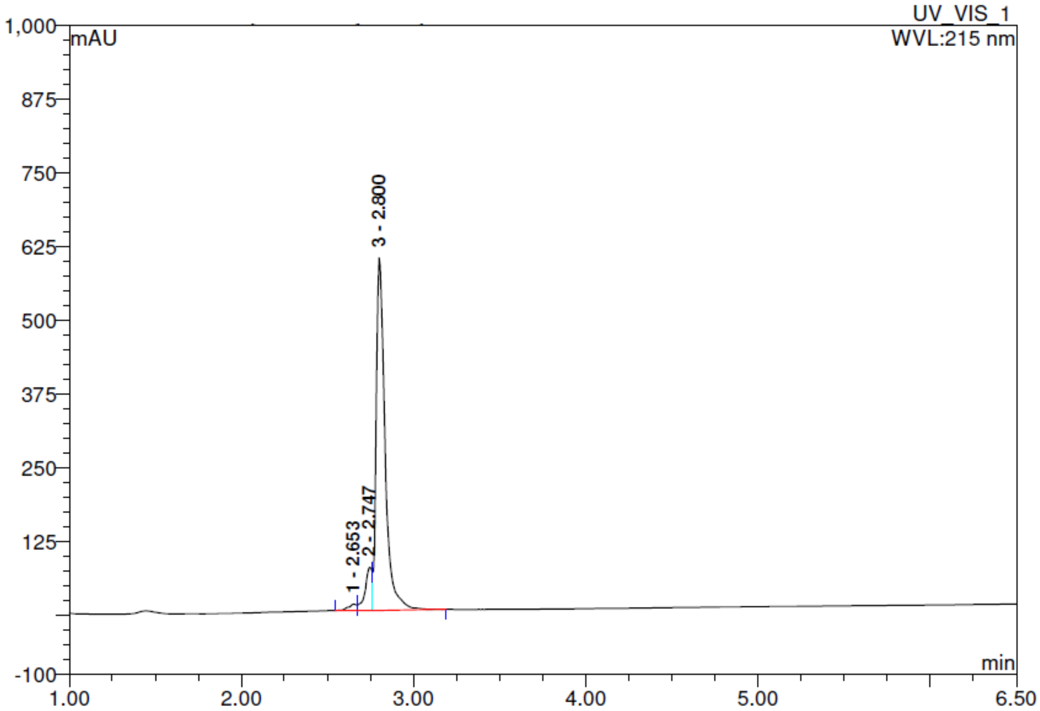

c) hCAP (146-157) (**A3**)

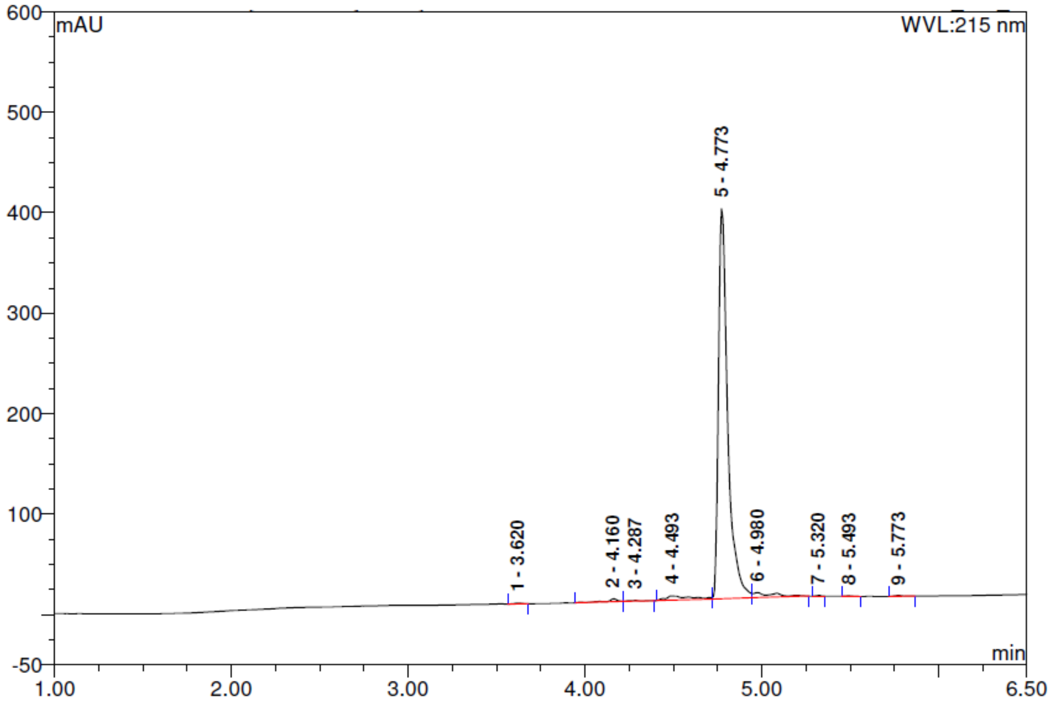

d) hCAP (152-163) (**A4**)

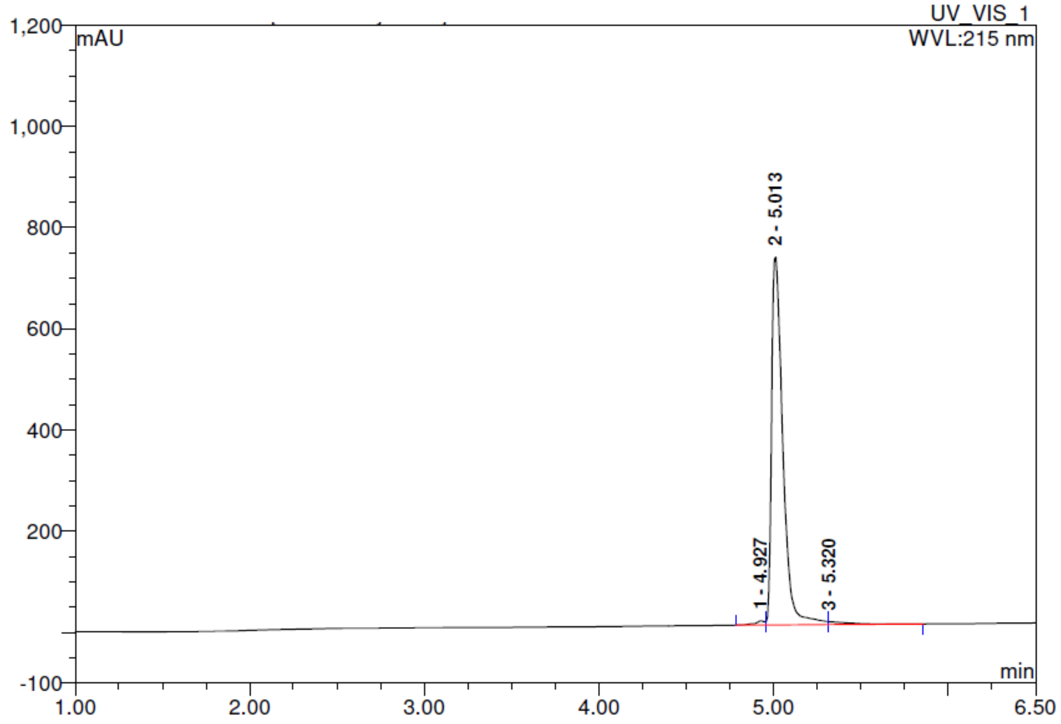

e) hCAP(159-170) (**A5**)

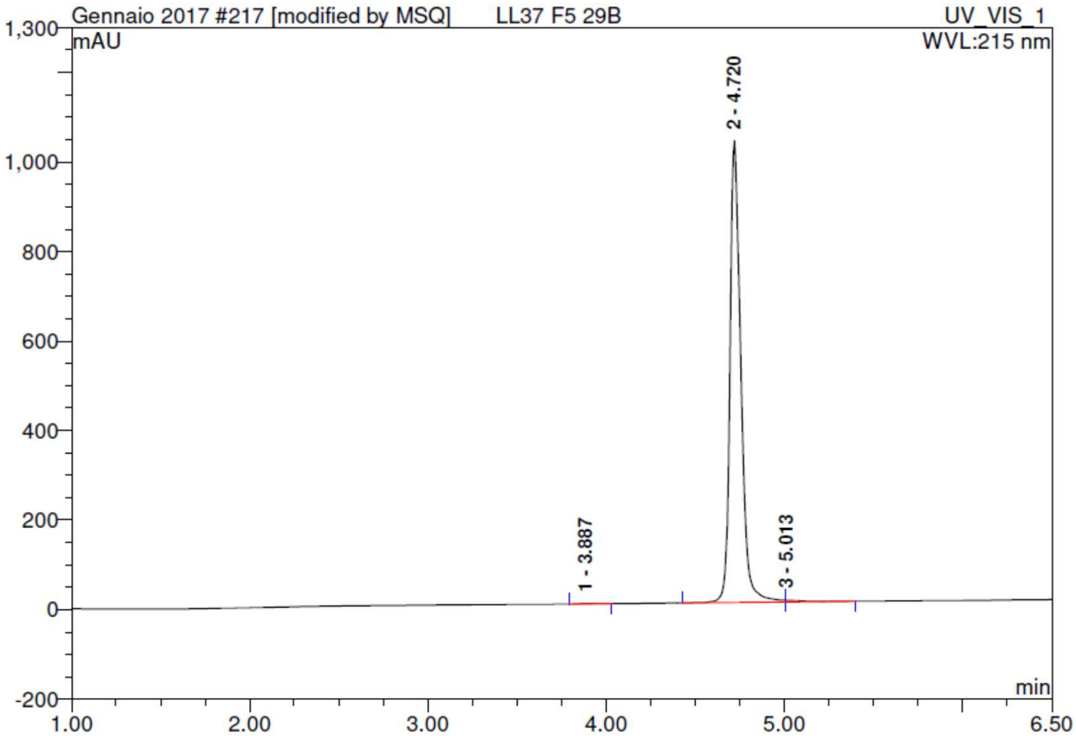

Figure S2. ESI-MS spectra of five LL-37 peptide fragments studied in this work.

a) hCAP (134-145) (**A1**)

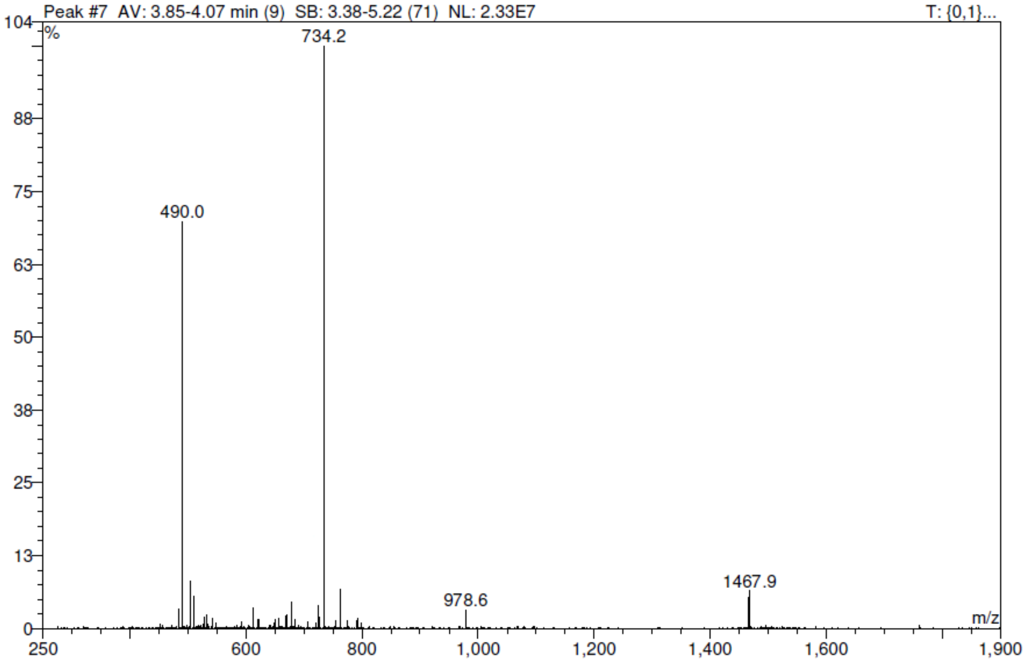

b) hCAP (140-151) (**A2**)

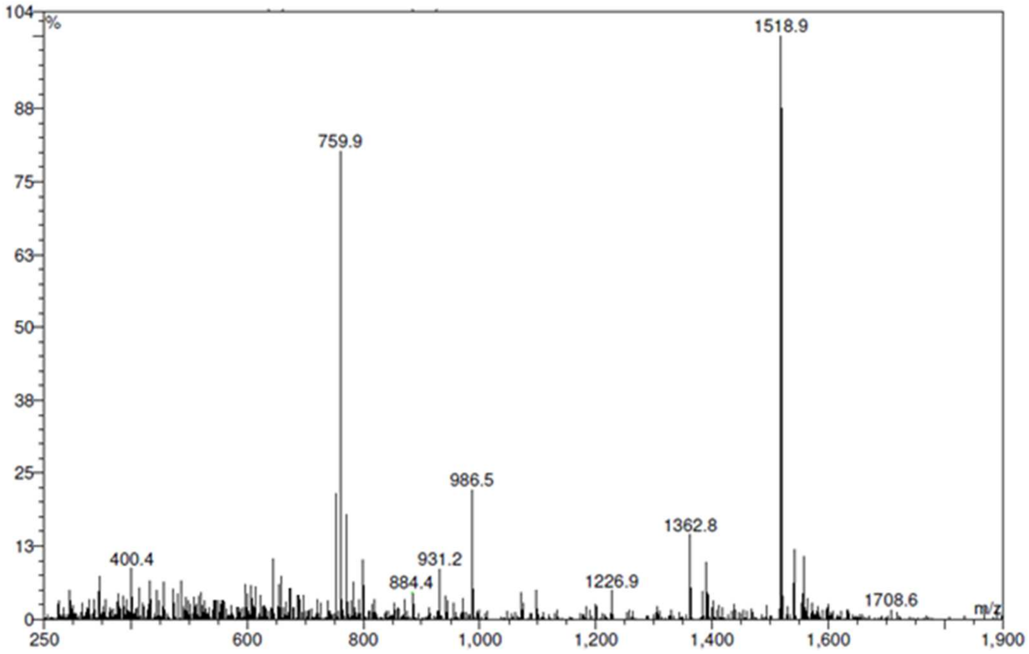

c) hCAP (146-157) (**A3**)

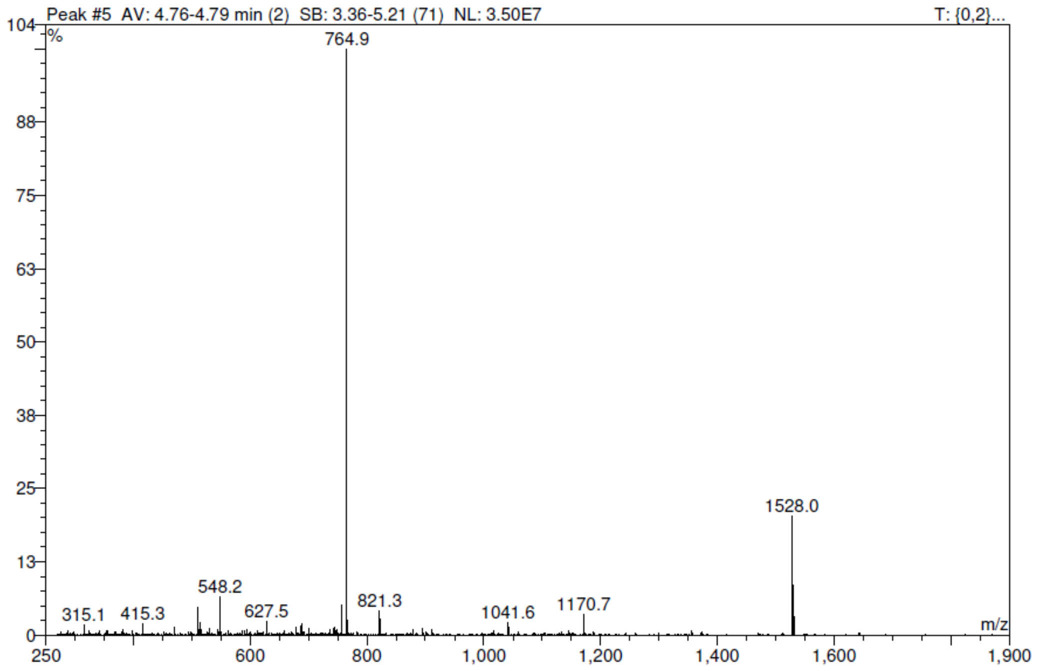

d) hCAP (152-163) (**A4**)

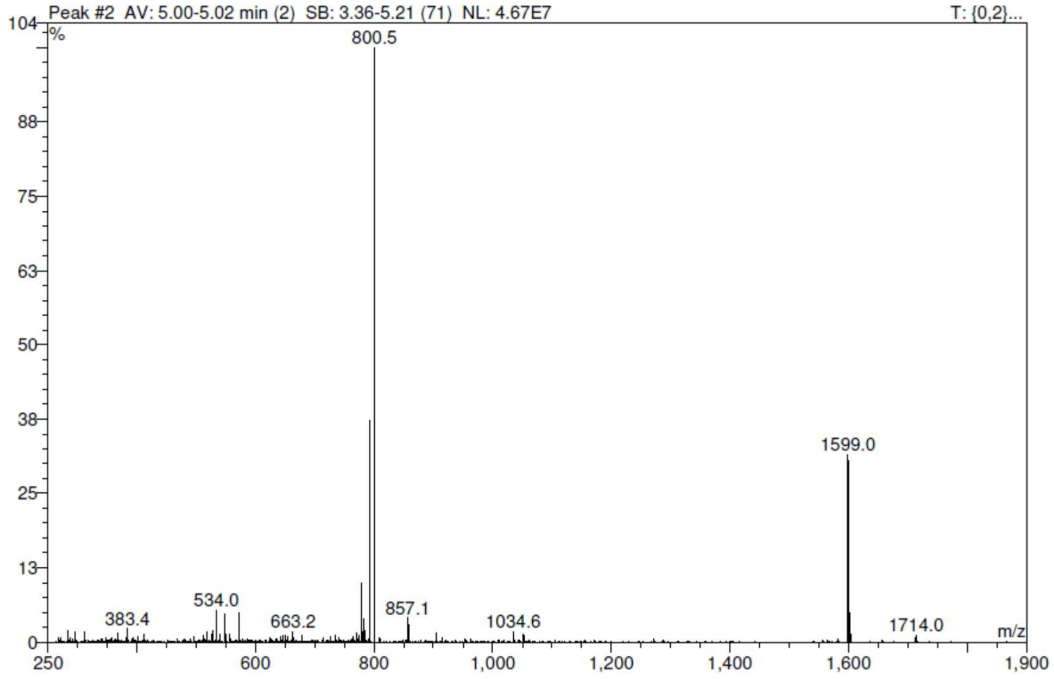

e) hCAP(159-170) (**A5**)

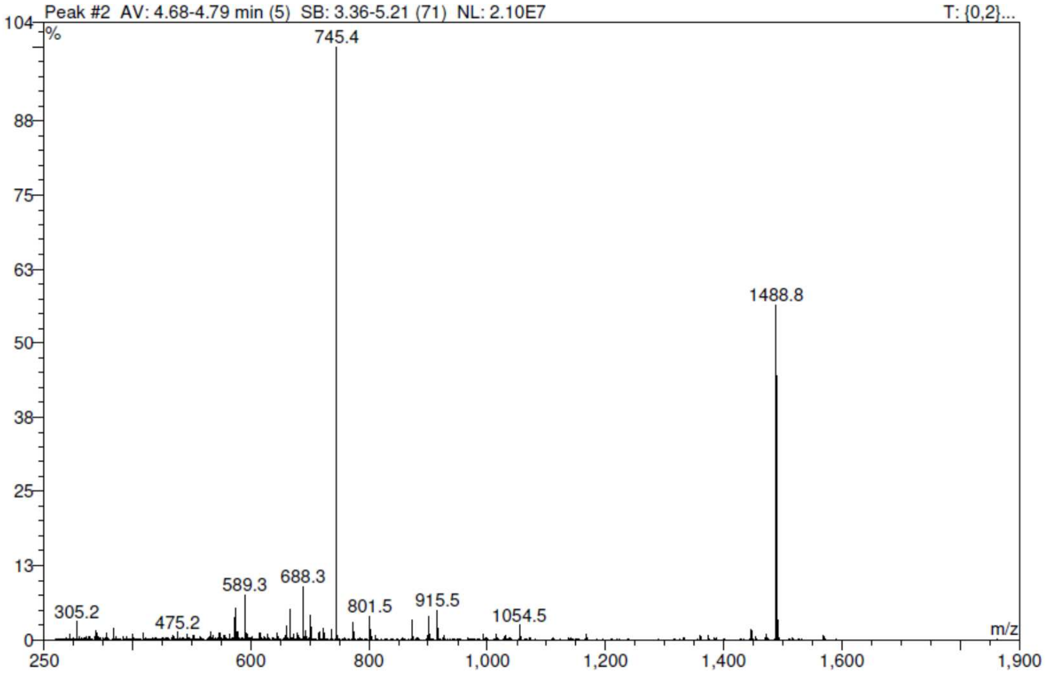

Figure S3. Calorimetric titration isotherms of the binding interactions of Zn(II) and Mn(II) metal ions with A3 and A4:

Zn^{2+} (1 mM) / A3 (0.1 mM)/ MES **Mn^{2+} (1 mM) / A3 (0.1 mM)/ MES**

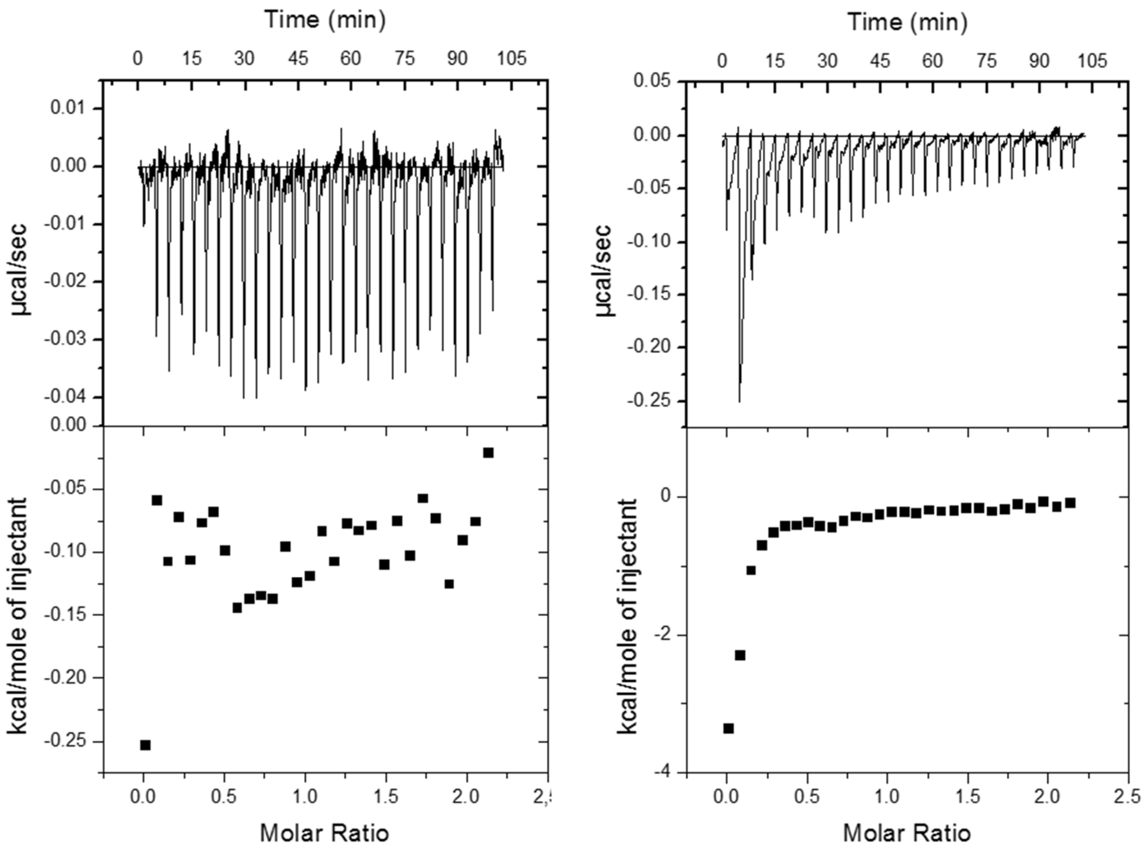

Zn^{2+} (1 mM) / A4 (0.1 mM)/ MES

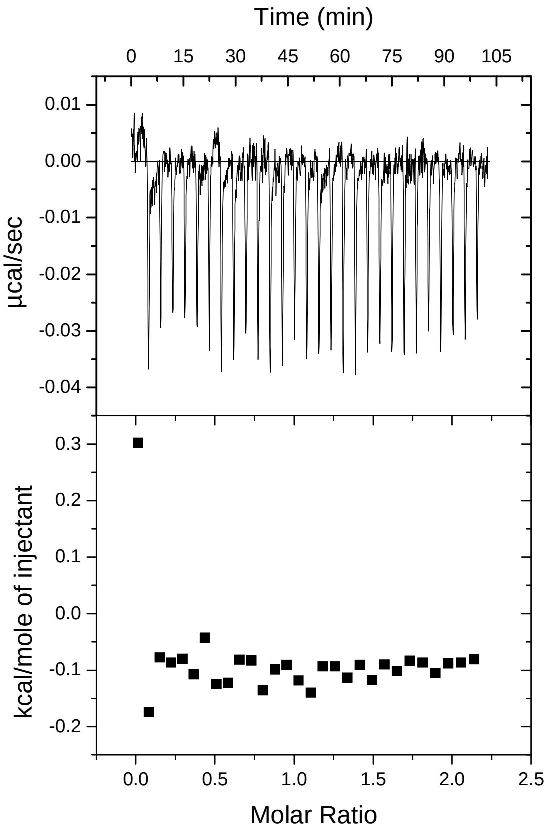

Mn^{2+} (1 mM) / A4 (0.1 mM)/ MES

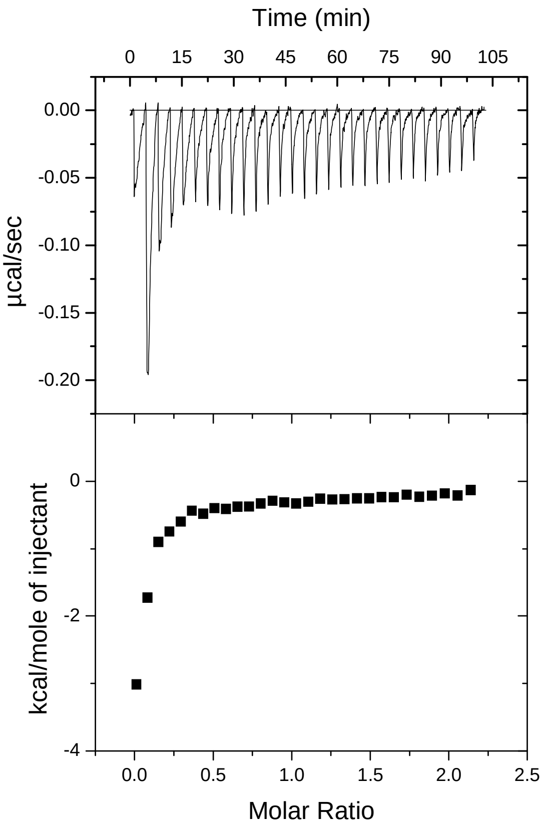

Table S1. Final Electronic Energies (in Hartree) - E, Gibbs Free Energies (in Hartree) - G, Enthalpies (in Hartree) – H, atom coordinates (in Å) calculated at the M06/SVP level (gas phase) level. Acronyms for the systems in the table as in Figures 3 and 4 of the main text.

System	Cooridnates [Å]			
	N	14.711979709242	2.006601344807	0.866653107328
	H	15.054010672411	1.199433687052	1.389283989249
	C	13.810214580171	1.601570856458	-0.178413151450
	H	13.504288057757	2.526837530340	-0.708513518940
	C	14.337407202816	0.659883510909	-1.274993673454
	H	15.100517573693	1.199918207033	-1.858445611282
	H	14.799390159751	-0.251874594453	-0.865126436256
	C	13.166268734395	0.270503201040	-2.131774211895
	O	12.899884505654	-0.944333009540	-2.335821376579
	O	12.285085266493	1.163029536178	-2.377820523799
	C	12.495985419430	1.131225570755	0.457972166986
	O	11.897397631128	0.075073702673	0.185309551817
	N	11.934877826896	2.025689244438	1.278551035760
	H	12.548312265014	2.779299052742	1.597650473268
	C	10.607393516916	1.883579720823	1.842358067276
	H	10.602828833966	1.172259313461	2.689098229432
	C	10.151598335389	3.276753448608	2.308450831689
**$(MnA5)^{2+}$ - 1**	H	10.837225832620	3.591745422893	3.116598832632
	H	10.315622803420	3.983551777081	1.473841354161
	C	8.728849843677	3.384475425884	2.779763897913
**E** = -6171.060825	C	7.791514107859	4.102671808959	2.031044026063
**H** = -6169.177962	H	8.096194470768	4.573295076763	1.088099879256
**G** = -6169.452034	C	6.489079090891	4.281326736732	2.500672625852
	H	5.781718971974	4.885549775893	1.924958391844
	C	6.107827838405	3.725788142065	3.721657500879
	H	5.100344519921	3.894815113134	4.113711679356
	C	7.029581209279	2.981448624854	4.462701370976
	H	6.739072583521	2.553009356467	5.425734821857
	C	8.329285936231	2.813989166757	3.994810483438
	H	9.055498417984	2.258363580642	4.598779263514
	C	9.578825930003	1.323686161442	0.866640481159
	O	8.691978783840	0.586562150650	1.260559141588
	N	9.616018994196	1.742450736192	-0.433694166749
	H	10.293593419133	2.453699037863	-0.706412986892
	C	8.429505809982	1.605842713886	-1.264557028078
	H	7.563565239820	2.081009275296	-0.759074208996
	C	8.645586555269	2.234237559052	-2.643852119405
	H	9.571484071932	1.810868115633	-3.088266698596
	H	7.816438885464	1.891614605294	-3.288853922506
	C	8.704053814897	3.763288689990	-2.720662572814
	H	7.783462676015	4.156456172731	-2.242817607217

	C	9.914269747694	4.373830809310	-2.018590358102
	H	10.849380928194	3.869173162771	-2.330260332246
	H	10.023441289625	5.435546244313	-2.288132510286
	H	9.840387290214	4.351993278234	-0.916861883930
	C	8.698558626672	4.171443573112	-4.187264639330
	H	7.824450470261	3.770882727479	-4.724215872542
	H	8.684248297998	5.266388799301	-4.295588460475
	H	9.605364698217	3.800732536573	-4.696259962363
	C	8.108367293780	0.133616773821	-1.476771477382
	O	9.026696834935	-0.679594442666	-1.740640754205
	N	6.837927603312	-0.216554175829	-1.471400682853
	H	6.102726834313	0.494946411095	-1.356410059179
	C	6.279206388500	-1.489465942158	-1.915423528002
	H	6.582453792337	-1.630861372827	-2.973115607213
	C	6.776942782922	-2.728446965942	-1.160492284730
	H	6.587036308561	-3.602684723154	-1.808683920662
	H	7.875612279213	-2.652809444490	-1.135773044860
	C	6.256934376444	-3.042296938681	0.238767719091
	H	5.232048455573	-3.455472433287	0.198152103672
	H	6.876756513135	-3.860460920838	0.638342049213
	C	6.264826261347	-1.895630785442	1.234830358530
	H	6.284911077998	-2.292004531968	2.266342314115
	H	7.175679956752	-1.284275135479	1.109129732388
	N	5.072933926896	-1.089770449497	1.036426530533
	H	4.213560906779	-1.574249334549	0.726801043273
	C	4.877659880696	0.169109297290	1.423701095429
	N	5.892580071026	0.950180640396	1.796273560567
	H	6.864297669820	0.636666975673	1.768104989926
	H	5.723288754754	1.862340366418	2.215864604343
	N	3.626362081813	0.635873559057	1.422764049523
	H	2.887940882955	0.013891763070	1.074404740301
	H	3.443565400132	1.627385530276	1.509917097635
	C	4.762651116826	-1.243900334867	-1.945063994576
	O	4.323627176310	-0.109064435926	-1.840063581183
	N	3.979490193525	-2.321149942642	-2.122618376549
	H	4.392519317704	-3.245846401579	-2.189235944790
	C	2.538683358854	-2.186126732112	-2.228325881874
	H	2.295200185748	-1.503959027487	-3.063144686047
	C	1.906628427495	-3.546438145268	-2.513497151813
	H	2.520333132309	-4.081190117833	-3.262426329140
	H	1.878037234736	-4.161748765446	-1.596835370968
	C	0.503646420567	-3.431150039311	-3.093491329077
	O	0.053083845810	-2.365619246927	-3.483530924476
	N	-0.157686630655	-4.596112845192	-3.212832239538
	H	-1.116563032287	-4.554576130094	-3.544155607997
	H	0.141916235275	-5.428584388425	-2.720479689156
	C	1.941101711131	-1.600168970674	-0.948300828179
	O	2.520492277074	-1.678804355327	0.141015402556

	N	0.731284031840	-1.058570846826	-1.097621979343
	H	0.314934300844	-1.170756349530	-2.031036231283
	C	-0.210233047225	-1.008782909015	0.004486160201
	H	0.206277951595	-0.394201947532	0.824702348075
	C	-1.516762254064	-0.398690718418	-0.518470842292
	H	-1.235963623359	0.491418216510	-1.115484992396
	H	-1.959623242936	-1.124901092988	-1.228105521132
	C	-2.577650262228	0.025458183457	0.495711221448
	H	-2.888583595183	-0.869956750310	1.066489047714
	C	-2.078001844571	1.097733619867	1.453742053929
	H	-1.786983245407	2.011278075748	0.903527934907
	H	-2.863524893400	1.384336060260	2.174695291718
	H	-1.202693948084	0.779441643340	2.050516499968
	C	-3.808805881647	0.507858614847	-0.258718042698
	H	-4.273503740123	-0.308955666868	-0.837747932540
	H	-4.565507868412	0.913465952436	0.433475383178
	H	-3.550505645025	1.320020407761	-0.961702014463
	C	-0.432884601759	-2.443833729287	0.505423043749
	O	-0.166862128111	-3.410792045490	-0.190870296106
	N	-0.972249808228	-2.549240598433	1.738607374750
	H	-1.298009317131	-1.690239510545	2.173183501111
	C	-1.654762922720	-3.765227997605	2.143362712097
	H	-0.916998099446	-4.586564243908	2.167548536008
	C	-2.228496197571	-3.562476385829	3.564661078226
	H	-1.354900014856	-3.248209241497	4.170925339248
	C	-3.285798167315	-2.466329837325	3.636926116028
	H	-4.178437941650	-2.743563728993	3.051893250585
	H	-3.601408643968	-2.325905806749	4.683033055313
	H	-2.944710272088	-1.479472593856	3.275301735494
	C	-2.788763737098	-4.840814972799	4.167293860377
	H	-2.039001968162	-5.647429132319	4.211057353338
	H	-3.119356661033	-4.654223656316	5.200697677546
	H	-3.672485324298	-5.190997255309	3.605081901384
	C	-2.749670831329	-4.064759905099	1.110771388384
	O	-3.318995694564	-3.135215261793	0.552350640415
	N	-3.113734898902	-5.343491258007	0.873600723056
	C	-2.527847877878	-6.596091219221	1.340351069577
	H	-2.073282742408	-6.493989379502	2.333971775091
	H	-1.736170513518	-6.930625857482	0.639742924269
	C	-3.715249196144	-7.543143654768	1.326687310834
	H	-4.326261716995	-7.377463867385	2.229308945033
	H	-3.419954474384	-8.602312239048	1.298607467343
	C	-4.486550807493	-7.095559187437	0.091764373273
	H	-5.544838597409	-7.397480102664	0.104638478909
	H	-4.035640963871	-7.522917918217	-0.820095897165
	C	-4.324218658889	-5.571657459142	0.079109585148
	H	-4.187233507833	-5.177409915052	-0.943859163064
	C	-5.492912497756	-4.852853125493	0.758097707635

	O	-5.714697826705	-5.010756086982	1.946757282397
	N	-6.231666639064	-4.070803765463	-0.058686441669
	H	-5.829285233191	-3.799615776782	-0.957263265115
	C	-7.137328206055	-3.062742172664	0.454779328429
	H	-7.963999961348	-3.544319621194	1.007745160679
	C	-6.378051353923	-2.081471040655	1.365890978692
	H	-6.238685540260	-2.577460605102	2.342200581857
	H	-5.368025714473	-1.973607593133	0.928313507145
	C	-6.952389436037	-0.690710043619	1.564818448744
	H	-6.955078820652	-0.144103384642	0.599731679010
	H	-8.001514341966	-0.700787123638	1.915409515388
	C	-6.076524305323	0.064319800720	2.548478826616
	H	-6.266915347291	-0.337993351872	3.566375078044
	H	-5.011688354417	-0.167694599066	2.324966252543
	N	-6.306242107070	1.483677727324	2.491223292928
	H	-6.886290011699	1.857823764513	1.741780924797
	C	-5.547447176658	2.422293786329	3.133306990424
	N	-4.717087717412	1.916607599139	4.120142818089
	H	-5.053318072416	1.095023332751	4.614417763581
	H	-4.340431268646	2.618210528882	4.748289664338
	N	-5.605813839691	3.657367143052	2.769479412164
	H	-7.084334279631	3.983714133953	1.959952692816
	H	-5.081922896780	4.274637560589	3.387156602960
	C	-7.668238140122	-2.300349387304	-0.748577851260
	O	-6.948628202904	-2.082243771733	-1.709543997132
	N	-8.933410982348	-1.839197455613	-0.624564808408
	H	-9.399774786057	-1.902616136800	0.284584292776
	C	-9.434963485583	-0.740317256817	-1.420397738203
	H	-8.574184933650	-0.211149962699	-1.873311374980
	C	-10.382006025516	-1.195310623684	-2.543001159451
	H	-10.669854900584	-0.290222823475	-3.111722675954
	C	-9.736592559558	-2.187367652761	-3.483540809507
	H	-9.491859088782	-3.120772554139	-2.947807567693
	H	-10.431961892435	-2.435368443793	-4.298912429155
	H	-8.799506859989	-1.798509609616	-3.909696879152
	O	-11.577559303427	-1.685180232079	-1.996301047242
	H	-11.377429759628	-2.508148609188	-1.531624953825
	C	-10.177554479453	0.183688049536	-0.451801352768
	O	-10.439585960733	-0.185366674913	0.684196824203
	N	-10.500516381701	1.386496595716	-0.954814164014
	H	-10.322151792318	1.605145104810	-1.936933895243
	C	-11.376866344611	2.347731891721	-0.319946232763
	H	-12.080121106429	1.804339094984	0.339246238806
	C	-10.603492686825	3.392062584068	0.494109243691
	H	-9.756063477681	3.731686822650	-0.125822905774
	H	-11.240224470836	4.278162362618	0.663797162965
	C	-10.103870728030	2.855099772458	1.828268506059
	H	-10.898537684550	2.856956232083	2.590238251251

	H	-9.802518572510	1.796798049305	1.711693414574
	C	-8.902835226737	3.553148361498	2.409337283562
	O	-8.777441914991	3.833468250838	3.573567803382
	O	-7.948540312607	3.742579613523	1.493690448806
	C	-12.148987598601	3.024885483159	-1.450105987091
	O	-11.749595038748	2.978758838065	-2.604393784996
	N	-13.260019786701	3.686391462097	-1.073218277283
	H	-13.494955329041	3.830550748494	-0.087974887896
	C	-13.987843348589	4.541694553534	-1.971991755695
	H	-13.274847597807	5.158073497910	-2.559189019587
	C	-14.826116680053	3.748236624150	-2.979166267504
	H	-14.203492761500	2.931031320665	-3.382856503343
	H	-15.687940621642	3.286968051210	-2.466530464295
	O	-15.330323748040	4.575633108852	-3.998730660249
	H	-14.633720955913	4.722497215965	-4.649664519144
	C	-14.833160293826	5.477548259673	-1.102403736092
	O	-14.710324125301	5.479679679797	0.109012300855
	N	-15.684490756403	6.267553164629	-1.784003596487
	H	-15.840957938305	6.103788152204	-2.777178380209
	H	-16.309560532200	6.860404248210	-1.248848845951
	H	16.397447295151	3.168121741556	1.319218159176
	H	16.509427604084	2.377326007660	-0.272808021436
	H	15.414533681629	3.765949668371	-0.030661170747
	C	15.812446291470	2.853941768592	0.443602992215
	Mn	11.001220984230	-0.365198374352	-1.692909611941
**(MnA5)$^{2+}$ - 2**<br><br>**E** = -6171.050323<br>**H** = -6169.166838<br>**G** = -6169.437862	N	14.898149398459	1.023004547582	0.700262285642
	H	14.813350913934	0.030814300619	0.479395715821
	C	13.918181026752	1.785328846822	-0.019725772797
	H	14.060721586404	2.851154512645	0.238370682164
	C	13.938637219996	1.664276963437	-1.570411603553
	H	14.644075367144	2.405338730309	-1.977685575892
	H	14.289859727392	0.662285689864	-1.864718372556
	C	12.543245974949	1.913964909124	-2.094997827160
	O	11.965109670214	0.906290488860	-2.679369019954
	O	11.956688078885	2.947377071418	-1.782740778484
	C	12.564412482815	1.365033362292	0.516466263309
	O	12.089424994986	0.230433380623	0.262160601058
	N	11.862136645989	2.256023505930	1.211110422269
	H	12.285103046849	3.171942993972	1.347908387456
	C	10.529680504084	2.049461675083	1.748972925162
	H	10.570685390754	1.379011611075	2.626464677946
	C	9.973523470550	3.425986460010	2.150795435192
	H	10.681306031664	3.856295645000	2.884478968350
	H	10.010950378169	4.081394237373	1.259588836893
	C	8.586496242348	3.433709876044	2.731614774416
	C	7.571934019260	4.179932211199	2.125853618080
	H	7.785780842790	4.744529801774	1.211177828867
	C	6.304140955885	4.263746643004	2.706681313012

	H	5.535333515663	4.888284066489	2.242011786686
	C	6.036300325519	3.587088313845	3.895367145549
	H	5.054646767695	3.673713216603	4.370376467231
	C	7.038954843397	2.822470115898	4.497550767342
	H	6.839273036468	2.300676514096	5.437320522711
	C	8.302866550403	2.750074747031	3.921453149709
	H	9.089634372292	2.173913543460	4.421183949681
	C	9.542264809281	1.385624056517	0.801503270397
	O	8.676158871228	0.655383805634	1.246174172463
	N	9.593709939732	1.671903804623	-0.545032791116
	H	10.227575064750	2.414336569203	-0.891824253985
	C	8.373932620729	1.434768054409	-1.323617670031
	H	7.508673736699	1.882567918048	-0.793597552801
	C	8.498007275688	2.036060531132	-2.729819270718
	H	9.538974120558	1.899234440173	-3.086035565604
	H	7.861775035000	1.450191438633	-3.417758499928
	C	8.088146901919	3.506975138223	-2.842879265284
	H	7.005448080838	3.558893353135	-2.604594639512
	C	8.830711966103	4.421207418692	-1.879000808297
	H	9.922305621727	4.368440096550	-2.041458980464
	H	8.526077006601	5.468327716534	-2.031260533643
	H	8.620600869362	4.181008213758	-0.820650284418
	C	8.288443295305	3.967390984361	-4.277656887218
	H	7.748272388957	3.329050157547	-4.994846007859
	H	7.935499215565	5.000453058862	-4.417252041010
	H	9.359280555431	3.943252193723	-4.544508945690
	C	8.128993776263	-0.060872037372	-1.437229432423
	O	9.095787169084	-0.852465013307	-1.576912725977
	N	6.874482479743	-0.460692135400	-1.461560298526
	H	6.110604801855	0.230339780810	-1.442291838326
	C	6.373471324833	-1.786890009667	-1.805062210403
	H	6.715880861407	-2.015051765713	-2.835196527106
	C	6.882652553125	-2.930653371691	-0.919676815847
	H	6.733262217327	-3.869889726975	-1.481747458056
	H	7.978047183093	-2.820572724413	-0.874826472193
	C	6.330593203619	-3.118805094294	0.489969916589
	H	5.315527574214	-3.556303094002	0.462624791257
	H	6.954739565117	-3.880366960246	0.983590600560
	C	6.287365137490	-1.881511823137	1.370579309198
	H	6.286482416921	-2.176862018548	2.435816048369
	H	7.189700031705	-1.264946147985	1.209365862465
	N	5.087112157054	-1.124490170979	1.063597242218
	H	4.250052811117	-1.651889442632	0.760154434390
	C	4.847035461944	0.152263454987	1.355509325416
	N	5.828936822890	0.978539402631	1.723908328344
	H	6.802453465577	0.677594967598	1.769887534073
	H	5.626153190598	1.906850073385	2.090057788204
	N	3.589742693385	0.588690131121	1.260745798279

	H	2.880090400880	-0.076304122329	0.930255344227
	H	3.377382360497	1.577800484314	1.287233051089
	C	4.851214596934	-1.594917161650	-1.900751633681
	O	4.376871207778	-0.469460018205	-1.911783497626
	N	4.101913640839	-2.706166645803	-1.993777395649
	H	4.538834652134	-3.621965232720	-1.975258483272
	C	2.660727608335	-2.614970254876	-2.139219296571
	H	2.419305945746	-2.044459664179	-3.054358184670
	C	2.052956578598	-4.009697049093	-2.263490196913
	H	2.682545766231	-4.622913249222	-2.935223394039
	H	2.020889172865	-4.508910334883	-1.278888031707
	C	0.655878766282	-3.983333173960	-2.869258089675
	O	0.205039579464	-2.982876731371	-3.403336149722
	N	0.004518595776	-5.160562414307	-2.843133683122
	H	-0.952709795431	-5.166332996956	-3.181756183880
	H	0.293484715431	-5.908125076057	-2.224179354786
	C	2.032005091324	-1.891565767526	-0.948543757578
	O	2.583235431752	-1.851891856889	0.158146642321
	N	0.827490519499	-1.372347015215	-1.185247677990
	H	0.430035759635	-1.592643959066	-2.107303690624
	C	-0.136375303381	-1.199110248626	-0.114889501077
	H	0.262344538836	-0.494419322509	0.638838391013
	C	-1.436199944544	-0.655593052902	-0.722747318000
	H	-1.144627773060	0.122634101135	-1.455391748128
	H	-1.904806369683	-1.475592581598	-1.300846760248
	C	-2.463654555283	-0.043757240150	0.226927942777
	H	-2.796060906149	-0.829655187829	0.929561460559
	C	-1.905707595580	1.144807936746	0.998329229424
	H	-1.448982877173	1.876360430787	0.305606443204
	H	-2.705412705810	1.682679499154	1.538295878645
	H	-1.132793221312	0.860959154837	1.737446825542
	C	-3.696475368502	0.358563518254	-0.568066596156
	H	-4.171544302001	-0.509489689944	-1.057413758437
	H	-4.439038310686	0.831771764531	0.095508949494
	H	-3.444482776519	1.098174207553	-1.349170014811
	C	-0.374772839703	-2.570520762988	0.532733515909
	O	-0.095625517187	-3.606008977590	-0.051971172484
	N	-0.956866424513	-2.550626688277	1.751459899479
	H	-1.298977912584	-1.651578101197	2.080730499429
	C	-1.695558234032	-3.713601401692	2.213374862429
	H	-0.987429633332	-4.553464381612	2.322357239369
	C	-2.322552606318	-3.403775737263	3.590786606635
	H	-1.465048894248	-3.092554314922	4.220977532303
	C	-3.330867754395	-2.261746462626	3.552989253775
	H	-4.207253690774	-2.524311386323	2.937101862146
	H	-3.691006088796	-2.049649601799	4.571773162995
	H	-2.924933621809	-1.311813016798	3.161946449439
	C	-2.964512836177	-4.624820663324	4.230540177950

	H	-2.250354292475	-5.454544448400	4.359336596834
	H	-3.342619495195	-4.368686301556	5.232194386783
	H	-3.829741377184	-4.976857230541	3.640680580984
	C	-2.755284772220	-4.043609468347	1.154604912663
	O	-3.276212211409	-3.132557093344	0.522942388988
	N	-3.139365016862	-5.323827190187	0.965304801344
	C	-2.605052801175	-6.563080560334	1.518794305284
	H	-2.203018514409	-6.422659673071	2.530433766006
	H	-1.782733444087	-6.942989819316	0.878614502862
	C	-3.809939453399	-7.487044727918	1.482393599353
	H	-4.464661337671	-7.266554084027	2.341447927764
	H	-3.536961242210	-8.551965204306	1.518906723698
	C	-4.505035651440	-7.082364417322	0.188509018849
	H	-5.569090102801	-7.361706979784	0.160327577559
	H	-4.017366118624	-7.562210916487	-0.677282070382
	C	-4.307073321313	-5.564413128439	0.113001349556
	H	-4.100577971004	-5.223688243513	-0.917721437119
	C	-5.496734321684	-4.790894454919	0.686707489786
	O	-5.826223149039	-4.931700387424	1.851861021730
	N	-6.132345467035	-3.987979704755	-0.194392214120
	H	-5.635750190433	-3.724399099413	-1.046108907825
	C	-7.076652266425	-2.977165072610	0.232883984588
	H	-7.925582275165	-3.460422807075	0.749149256853
	C	-6.399155418873	-1.950522108643	1.162239961388
	H	-6.314155529487	-2.408828738934	2.163162318085
	H	-5.364432548035	-1.825337245107	0.788581735662
	C	-7.031934037163	-0.571723105111	1.265260511022
	H	-6.974139996831	-0.072112995124	0.276147220366
	H	-8.107878545673	-0.618693565663	1.520991131715
	C	-6.281825551294	0.278705623558	2.281611697176
	H	-6.699943683116	0.122505957689	3.290469606483
	H	-5.222420260739	-0.067176315385	2.330245218944
	N	-6.330294641815	1.697627036612	1.950679872256
	H	-6.029917177870	1.856084512357	0.988339558903
	C	-5.561136727501	2.550642341340	2.755140830717
	N	-5.953480223056	2.569619399504	4.065119306412
	H	-6.919606376284	2.326819394227	4.295533431333
	H	-5.445859855978	3.158934425456	4.711460774636
	N	-4.586119338136	3.191227133694	2.218374534370
	H	-8.225664669876	4.341413855545	3.312883665588
	H	-4.183976204902	3.860293060522	2.880026947236
	C	-7.544092892918	-2.249588250197	-1.015718475377
	O	-6.756958404113	-1.976489577099	-1.906708322676
	N	-8.842217284933	-1.865701075875	-1.002243945126
	H	-9.388233906515	-2.008075405077	-0.147978395117
	C	-9.316831914472	-0.726498634351	-1.758867645409
	H	-8.441685512298	-0.129609349440	-2.081584718512
	C	-10.133648166844	-1.110258403959	-3.002519748520

	H	-10.380097759438	-0.167228644740	-3.527821996571
	C	-9.366462601973	-2.016781370968	-3.938224586516
	H	-9.153501409486	-2.980633337342	-3.444865858513
	H	-9.965227452982	-2.217721048093	-4.838675429805
	H	-8.399879980450	-1.579796694358	-4.231094803333
	O	-11.368307431786	-1.657864970337	-2.625835300985
	H	-11.198333847286	-2.490717584690	-2.166272499157
	C	-10.183174553508	0.073711326320	-0.785587301531
	O	-10.603606777440	-0.442775240462	0.238632163459
	N	-10.418854965743	1.355282049079	-1.124681584973
	H	-10.141070406938	1.735426315678	-2.031044883331
	C	-11.299391196952	2.212920759557	-0.364681091949
	H	-12.080356166963	1.584331828051	0.102588709248
	C	-10.562909289833	3.000936746530	0.726002705308
	H	-9.852124388680	3.693467985403	0.240051394616
	H	-11.289541938323	3.635988890203	1.263344540123
	C	-9.827509550189	2.099864844947	1.699379888623
	H	-10.481174635744	1.326344573313	2.131396913808
	H	-9.030280370348	1.544751962794	1.169588709346
	C	-9.100262632749	2.761484464700	2.823083887422
	O	-8.815257556217	2.208956539323	3.860778956364
	O	-8.745067485910	4.020683520214	2.557849365047
	C	-11.930424694510	3.185554935680	-1.355464361225
	O	-11.426547462703	3.384356996016	-2.450371454902
	N	-13.034414909287	3.819059516363	-0.915326973569
	H	-13.358543012357	3.735194463462	0.051166773799
	C	-13.635992178622	4.909959447072	-1.634264215376
	H	-12.843781980398	5.596327067478	-2.000476250445
	C	-14.419179534325	4.435552826526	-2.862663032998
	H	-13.802772231341	3.698072446607	-3.405371090250
	H	-15.342738616726	3.923605731631	-2.541119055896
	O	-14.793034105977	5.513041183201	-3.685400360735
	H	-14.041118215215	5.755632914896	-4.238775477721
	C	-14.509478657601	5.669261503916	-0.630506066594
	O	-14.495772437675	5.375514207559	0.551377091863
	N	-15.256878920145	6.654363371198	-1.163002738642
	H	-15.332597358090	6.740126387095	-2.175392646789
	H	-15.900176523791	7.143035858945	-0.549787565117
	H	16.930077743207	0.867652749610	1.178721430230
	H	16.650535998429	1.490088175227	-0.470200999177
	H	16.340588469771	2.521539831065	0.954273376616
	C	16.262649709925	1.491897209987	0.568542231251
	Mn	10.985442876621	-0.183771709284	-1.421545401945
**(MnA5)$^{2+}$ - 3**	N	-11.941934352594	1.724683319009	-0.601987910880
	H	-11.223947190328	1.946095980172	-1.288793055035
**E** = -6170.982064	C	-11.896125224433	0.344625673741	-0.235847130057
**H** = -6169.100285	H	-12.698243843302	0.151355521239	0.503917183632
**G** = -6169.369270	C	-12.109464302393	-0.676284571268	-1.389893299809

	H	-12.985979051097	-0.346047231690	-1.970469519129
	H	-11.229641984211	-0.673461066679	-2.060614080313
	C	-12.438123644410	-2.050867386819	-0.841639290359
	O	-13.457105767553	-2.245883702532	-0.237821371308
	O	-11.518444648353	-3.005835647247	-0.989338481504
	C	-10.553738277684	0.002139410208	0.371945079471
	O	-9.527215008319	0.636549794663	0.103261683535
	N	-10.504745090016	-1.126901462366	1.107830581075
	H	-11.368115340444	-1.616515779781	1.339961515746
	C	-9.240936307984	-1.718640665170	1.469446082015
	H	-8.694310973812	-0.997564367212	2.111375476717
	C	-9.429516134982	-3.011550581616	2.273266090408
	H	-10.267751259849	-2.851609065319	2.974187730903
	H	-9.735443080737	-3.823948600841	1.589469234245
	C	-8.195343448521	-3.375751542051	3.053634719994
	C	-7.287607400878	-4.334019069166	2.591514505065
	H	-7.499205591524	-4.867111240247	1.657859514519
	C	-6.128882372364	-4.622157049641	3.309940524607
	H	-5.440165326684	-5.389309276346	2.944916095579
	C	-5.862921432504	-3.958577860077	4.508881388887
	H	-4.972949018427	-4.214474192626	5.091766302709
	C	-6.766684946539	-3.007372335644	4.986633499752
	H	-6.591746621089	-2.515077428540	5.948362020704
	C	-7.921297421145	-2.718230315424	4.259252113082
	H	-8.640686162272	-1.988206529787	4.650036932925
	C	-8.347092644824	-1.941116194713	0.252888207014
	O	-7.066467403585	-1.927679685847	0.547609894902
	N	-8.862783705798	-2.078397774988	-0.916569942134
	H	-10.623463095354	-2.625373398040	-1.152057940229
	C	-8.066480744895	-2.222044034626	-2.112303452524
	H	-7.052683923780	-2.672655760954	-1.888980794874
	C	-8.710351606255	-3.146817345975	-3.138109692009
	H	-9.802734582663	-2.965945350741	-3.128397098696
	H	-8.357871562044	-2.856839215118	-4.141760333140
	C	-8.428143178566	-4.635108837439	-2.925805473077
	H	-7.339645368602	-4.782070620088	-3.093907151267
	C	-8.758199019344	-5.130340016803	-1.524135762225
	H	-9.821140478656	-4.955684817058	-1.279112492821
	H	-8.578179428114	-6.214611785506	-1.449194172357
	H	-8.143730939306	-4.644660492075	-0.744537117288
	C	-9.182765810957	-5.442668017565	-3.969064819625
	H	-8.954357405395	-5.106610502434	-4.993068972447
	H	-8.936221866060	-6.513449396574	-3.903226845891
	H	-10.272178832973	-5.344841030416	-3.818126550414
	C	-7.672378528753	-0.862591671942	-2.714340518679
	O	-7.410465265671	-0.662028647307	-3.864610056769
	N	-7.415036192912	0.130743145034	-1.710808884219
	H	-8.227105407854	0.257753806078	-1.067824865719

	C	-6.810484735768	1.391397168633	-2.142358704102
	H	-7.176087317399	1.638868925293	-3.158928900845
	C	-7.197045488181	2.524448416771	-1.180004494555
	H	-7.052083730944	3.487497790596	-1.698698959215
	H	-8.287573910002	2.446034254668	-1.029145485512
	C	-6.504502226401	2.634836822954	0.181922343280
	H	-5.461485243033	2.981956592679	0.061746668938
	H	-7.013118875486	3.458490272928	0.707932254774
	C	-6.508933345014	1.446058596069	1.140154987031
	H	-6.446717076247	1.820737906339	2.175327325149
	H	-7.441647405492	0.860831384990	1.063720947983
	N	-5.303689773318	0.567863751933	0.905896511106
	H	-4.556619066490	1.119548607992	0.429104913570
	C	-4.751955359810	-0.060929676417	2.014142797953
	N	-5.504439936195	-0.899303724599	2.696490862060
	H	-6.349384975661	-1.284001804846	2.241690549637
	H	-5.185627064043	-1.391563007491	3.532193699161
	N	-3.489981626892	0.214650171745	2.300772803188
	H	-2.985722747160	0.896430593768	1.717785493568
	H	-2.986027886637	-0.289634104790	3.024901792401
	C	-5.299935313654	1.176508622645	-2.263750409242
	O	-4.790576345308	0.057660784504	-2.015953494329
	N	-4.528915430981	2.209467645169	-2.563259011770
	H	-4.967990770482	3.103106871282	-2.773980890183
	C	-3.073667402102	2.120377284441	-2.601162607546
	H	-2.776059360831	1.424299721900	-3.405996022122
	C	-2.476405673777	3.495422013520	-2.899801353804
	H	-3.095790270842	4.009228092215	-3.658316190697
	H	-2.462658670160	4.120607853764	-1.989256771997
	C	-1.062205839435	3.398061908874	-3.460963398677
	O	-0.552596231576	2.321838031819	-3.733013835146
	N	-0.456762490021	4.574630609013	-3.678597776616
	H	0.493338897086	4.562353146492	-4.037451940711
	H	-0.852415635778	5.457482017144	-3.380079571644
	C	-2.502697215672	1.597465562862	-1.277532126134
	O	-3.111957768238	1.720948893996	-0.201618441670
	N	-1.289935178292	1.070437170376	-1.383705857782
	H	-0.848382696586	1.172702033731	-2.310287298998
	C	-0.380974709218	1.027203193337	-0.254270596851
	H	-0.804056582374	0.374350026023	0.532728213553
	C	0.958764599180	0.459607154291	-0.737598375862
	H	0.722514046085	-0.416435972070	-1.373395024298
	H	1.423463235547	1.214724845757	-1.400893794127
	C	1.973561380084	0.018399151523	0.316357723084
	H	2.258753182100	0.905090367777	0.914397124474
	C	1.433807497159	-1.070265738114	1.233726658833
	H	1.165875710720	-1.974251040917	0.656915255176
	H	2.187968274231	-1.369263626387	1.982388268268

	H	0.534870947486	-0.761161024769	1.801790598520
	C	3.235402257762	-0.453827762958	-0.392765547220
	H	3.724232710210	0.369837538471	-0.941167926329
	H	3.961678103199	-0.870225254846	0.324439156103
	H	3.005805468269	-1.256210124826	-1.116264761531
	C	-0.233477832060	2.460518566238	0.283373178036
	O	-0.525893366026	3.426314634573	-0.400124981383
	N	0.239003752253	2.551006558165	1.548509089511
	H	0.654166756171	1.701132380794	1.924587248088
	C	0.871007870955	3.780883489554	2.007609887851
	H	0.109725613794	4.580252095971	2.011196529534
	C	1.381093119200	3.558141905566	3.450859499955
	H	0.485895933881	3.213223285650	4.007762197151
	C	2.456042091843	2.481033095981	3.549569498721
	H	3.369665649139	2.787422876034	3.013766040379
	H	2.726400526393	2.325163180565	4.605848039665
	H	2.154636981556	1.493008820883	3.155917802922
	C	1.887859224862	4.830960443564	4.110067717194
	H	1.124704307844	5.625620679533	4.132478749405
	H	2.165983436084	4.624667725807	5.155024225139
	H	2.795538020818	5.204972630660	3.604345551242
	C	2.006359320301	4.122673306652	1.032421632444
	O	2.603008097327	3.210074464056	0.473078900541
	N	2.368492245463	5.408518833826	0.850340631241
	C	1.748386723773	6.642896100212	1.324555726665
	H	1.240390201631	6.505820989024	2.287473192476
	H	0.995076662630	6.994319430374	0.591269372784
	C	2.926666015828	7.597665633416	1.405875627502
	H	3.489582161570	7.409300440711	2.334867545171
	H	2.623676019680	8.654822814973	1.394420363548
	C	3.767202611927	7.195164368896	0.200830833832
	H	4.820531672346	7.503983374152	0.280507119658
	H	3.362631968023	7.648293872949	-0.720128065246
	C	3.619817804985	5.671180459374	0.131327178914
	H	3.543410256550	5.307894986163	-0.908998045421
	C	4.753724288226	4.940132837470	0.855001751452
	O	4.894853537446	5.051746331318	2.061366268731
	N	5.553093339152	4.201901666162	0.056790652004
	H	5.217064992271	3.965645170407	-0.878375780421
	C	6.450401155337	3.191606109273	0.580774671372
	H	7.242722004523	3.666381799199	1.187309230841
	C	5.667028746349	2.167780066929	1.422478616607
	H	5.481165239088	2.621925586008	2.411395370303
	H	4.677489330952	2.064871395950	0.938261954757
	C	6.253097629718	0.777245266709	1.586687397073
	H	6.299333063071	0.273796184276	0.599661425439
	H	7.289263090655	0.785023018663	1.973030424799
	C	5.353896999431	-0.033323215472	2.503468555014

	H	5.527367545945	0.295813362032	3.549788572810
	H	4.294527179627	0.221256570279	2.277757667521
	N	5.573832218882	-1.447767462868	2.349910535622
	H	6.160241574893	-1.769867965099	1.581222281992
	C	4.781214559469	-2.422804632879	2.889570161495
	N	3.963190147812	-1.991968165094	3.924277617400
	H	4.349085121161	-1.257343860623	4.511941790255
	H	3.572075714044	-2.748373352216	4.476390895360
	N	4.797843240129	-3.613008812529	2.397304004591
	H	6.333770648411	-3.916588550262	1.629583700818
	H	4.262834055671	-4.275765835277	2.956256776783
	C	7.048356100567	2.479647339519	-0.623264032520
	O	6.375270739151	2.289533237172	-1.623597102725
	N	8.312700092468	2.032767508737	-0.457693877963
	H	8.730717363107	2.061757732892	0.476443447061
	C	8.874140590410	0.977428029864	-1.272703341403
	H	8.049816635818	0.473577932678	-1.813462613475
	C	9.898236735404	1.497333654245	-2.295507267306
	H	10.249535020982	0.624832953103	-2.879169060956
	C	9.310137293095	2.520138273370	-3.240393886208
	H	8.977881133316	3.413280030535	-2.683294044029
	H	10.071904400443	2.835376749001	-3.968624438319
	H	8.436872063124	2.124577888151	-3.780937470926
	O	11.034879620659	1.980944974364	-1.630943628138
	H	10.791240276764	2.797927663564	-1.176929821277
	C	9.548132697042	0.001654803558	-0.303642862758
	O	9.710879519522	0.302445718297	0.870757501323
	N	9.926540997675	-1.164190572229	-0.851682714704
	H	9.834338794258	-1.320243477138	-1.857759786984
	C	10.767560071130	-2.155176906395	-0.214261373049
	H	11.415125383454	-1.648033047554	0.526010475597
	C	9.951281780350	-3.254804742684	0.475348760312
	H	9.153711281183	-3.559670518718	-0.223724979471
	H	10.583981604148	-4.145816826011	0.633125816775
	C	9.353392929359	-2.807784431218	1.802762056822
	H	10.087122532688	-2.870598802570	2.621080278271
	H	9.065706617541	-1.741106186762	1.738656905740
	C	8.109198842909	-3.531471409259	2.241254534042
	O	7.890528833343	-3.888949574920	3.369896280460
	O	7.224530531152	-3.647723295155	1.244487568152
	C	11.628542515058	-2.752405243636	-1.326401396366
	O	11.320742176006	-2.621132005347	-2.502046915043
	N	12.706849323725	-3.442563667599	-0.910375541426
	H	12.860195047789	-3.659480301269	0.077741520140
	C	13.505407267639	-4.239069102301	-1.803627256332
	H	12.842596147101	-4.800923781983	-2.494870001521
	C	14.435750984466	-3.384913658731	-2.670350511396
	H	13.856708516610	-2.533702456831	-3.068849560756

	H	15.248499504243	-2.970674168598	-2.048730828384
	O	15.027848255056	-4.142632491331	-3.696312567121
	H	14.397642761139	-4.228421505707	-4.421611149182
	C	14.262338224108	-5.247452059151	-0.932707503973
	O	14.029820386784	-5.334230391561	0.259645452815
	N	15.165553933854	-5.998030967719	-1.590478676890
	H	15.411994054176	-5.766208522218	-2.551325773757
	H	15.734839907854	-6.637423296185	-1.046575499280
	H	-13.177482106137	3.307798023512	-1.186328496062
	H	-13.701833523214	1.744552014379	-1.861956464199
	H	-13.944764616280	2.107867984075	-0.135016337576
	C	-13.242468829472	2.230509648211	-0.976006659773
	Mn	-5.842165089925	-0.996824635627	-0.634237692260
**(MnA5)$^{2+}$ - 4**<br><br>**E** = -6170.962234<br>**H** = -6169.081608<br>**G** = -6169.349744	N	10.684175389461	-0.613177067471	3.549372253193
	H	9.713417718197	-0.492973063624	3.832556446687
	C	10.790564570334	-1.194603341248	2.243201943361
	H	11.843040332440	-1.124365856659	1.910223356093
	C	10.362693648896	-2.682851768267	2.214322401920
	H	10.758131349661	-3.195260538561	3.105152495898
	H	9.258185105004	-2.762158562430	2.280937250189
	C	10.789366248454	-3.496674337939	0.967803650399
	O	10.737588424266	-4.718411634221	1.133305930565
	O	11.056780785299	-2.869534029152	-0.090198678025
	C	9.905337764909	-0.387205031535	1.302539249011
	O	8.830041392693	0.073224006992	1.672246732647
	N	10.363593197301	-0.266550850782	0.032915363484
	H	11.048550068204	-0.996176912091	-0.217185583244
	C	9.454263691127	0.159556646213	-0.999537875552
	H	9.046834622228	1.153204344530	-0.731045759200
	C	10.168786488697	0.273708390066	-2.350753525563
	H	11.093814196716	0.852162916599	-2.185974270855
	H	10.475736969087	-0.737116697563	-2.680365714719
	C	9.309673522154	0.943451871211	-3.386234352850
	C	8.546743600719	0.200399680847	-4.292106315424
	H	8.613280601724	-0.893221959269	-4.278363031923
	C	7.703655338385	0.833518631547	-5.202209097729
	H	7.124634196371	0.235272439331	-5.911592721652
	C	7.614582107124	2.225953358012	-5.223626303316
	H	6.977036078080	2.724816794139	-5.960328676260
	C	8.376953640726	2.979356099259	-4.329689399124
	H	8.336200589508	4.072554723020	-4.356480267380
	C	9.214599367972	2.339463820003	-3.418300081091
	H	9.819716409761	2.933504155366	-2.723225447024
	C	8.238794086601	-0.753619973799	-1.118900323528
	O	7.150682787700	-0.293194937440	-1.502739723729
	N	8.407854946181	-2.034421224683	-0.805134622266
	H	9.356421357837	-2.378510121131	-0.567355291973
	C	7.297646369551	-2.982389697799	-0.726267934237

	H	6.635586998213	-2.808067324084	-1.597105685290
	C	7.795115053203	-4.415841386759	-0.701065105739
	H	8.584329314431	-4.509883751140	0.068740256410
	H	6.970145184861	-5.043714203861	-0.322210133998
	C	8.288476686389	-5.009228331097	-2.021004448837
	H	7.412003425181	-5.092441956221	-2.698573269787
	C	9.352922808291	-4.179834837293	-2.727941141285
	H	10.219905645963	-4.001273685020	-2.065491840399
	H	9.717072639620	-4.709775212804	-3.623163364096
	H	8.970233516631	-3.200072627750	-3.063867679755
	C	8.822966081203	-6.405675676347	-1.737134743259
	H	8.065387358863	-7.043909529979	-1.253200757312
	H	9.152324466367	-6.909810934981	-2.659533583374
	H	9.688498761542	-6.339480682598	-1.053863629985
	C	6.532555446985	-2.654260115125	0.564894009212
	O	6.729211682993	-3.236060226170	1.614065043522
	N	5.637223419894	-1.627373819584	0.469050988004
	H	5.589099882117	-1.093839933669	-0.397576293886
	C	4.951791919334	-1.188897916194	1.651674236828
	H	4.771307228928	-2.111773264547	2.242470054183
	C	5.791324650316	-0.289193035024	2.582823070381
	H	5.444915010000	-0.427652582906	3.625008472869
	H	6.804944779148	-0.723698318309	2.568442324091
	C	5.911734488237	1.209460103641	2.315115739764
	H	5.016359846503	1.760922313664	2.666950028977
	H	6.741188392916	1.570155825730	2.943598105556
	C	6.216669173733	1.631686323115	0.889805130565
	H	6.759857184166	2.594694585159	0.881347819772
	H	6.875546549355	0.886982726665	0.423480919899
	N	4.981715956121	1.777023499860	0.114933018497
	H	4.107424286331	1.890343521542	0.631951694170
	C	4.928523900443	2.140008987780	-1.169416732032
	N	6.006104625007	2.077600913428	-1.939801792511
	H	6.692868583297	1.311536193596	-1.757608568689
	H	5.955426748649	2.384082712112	-2.907910344257
	N	3.765655345276	2.626483527240	-1.641238358759
	H	2.956831225146	2.637527223589	-1.018473880146
	H	3.618951250780	2.657965631185	-2.642001374895
	C	3.579017127814	-0.666949884596	1.296109205675
	O	3.056564515125	-0.745998842435	0.201204201719
	N	2.901904097813	-0.069731044499	2.333614977331
	H	3.300092594212	-0.092896562285	3.267242127862
	C	1.519499153504	0.280814407066	2.154830988519
	H	0.889839590588	-0.628442243573	2.070362260796
	C	1.075578508357	1.095772817072	3.364853739870
	H	1.394735202844	0.568252591376	4.283646533372
	H	1.623330847192	2.057486790941	3.359892872498
	C	-0.392195176168	1.395088449825	3.537782424756

	O	-1.292217535642	0.933181027651	2.816388646908
	N	-0.674030466691	2.212692006815	4.558599136628
	H	-1.646063216995	2.380842268769	4.804450526093
	H	0.046006993514	2.572836098664	5.176993357305
	C	1.341056716855	1.117732148875	0.880944877755
	O	2.017529421219	2.112706399832	0.654984340312
	N	0.289788166279	0.739302130808	0.129218505474
	H	-0.160172718071	-0.132334898557	0.390033948019
	C	-0.415142953921	1.566860337369	-0.816020300996
	H	0.173410624599	1.734376226928	-1.740090483950
	C	-1.702006465191	0.780323919400	-1.137370937898
	H	-1.374754914576	-0.183819993533	-1.573822849437
	H	-2.157932713338	0.516205309603	-0.154509593330
	C	-2.800216191121	1.365878918743	-2.011042142072
	H	-3.148072210444	2.308658512592	-1.542189723597
	C	-2.346191271537	1.646349909354	-3.434225744485
	H	-1.989460812161	0.721718189961	-3.920495984485
	H	-3.183078888873	2.030055390668	-4.041230006972
	H	-1.526168490792	2.385330739611	-3.501249958982
	C	-3.970589347529	0.391859477234	-2.003326356773
	H	-4.342279107973	0.226425314350	-0.975272219961
	H	-4.806958201824	0.753329894706	-2.619598650767
	H	-3.681699914193	-0.588933251025	-2.419966972799
	C	-0.739777691818	2.914704165201	-0.168139129652
	O	-0.825446440080	3.006178337142	1.067849043828
	N	-1.040623826886	3.941683585355	-0.967694190085
	H	-0.982990074342	3.801695194658	-1.974816816065
	C	-1.650531362140	5.189577044117	-0.507399304341
	H	-0.858347972036	5.847184283214	-0.104261216277
	C	-2.322685006465	5.892556681392	-1.716896332912
	H	-1.552020857905	5.855963122104	-2.514015914854
	C	-3.575478159710	5.178134782048	-2.205278662191
	H	-4.389027255871	5.251661156157	-1.461349775941
	H	-3.935721609879	5.654373154933	-3.129760645588
	H	-3.411700891539	4.113070131318	-2.429826830848
	C	-2.633534485993	7.357779002900	-1.463150047526
	H	-1.749842379258	7.934214108615	-1.144948734936
	H	-3.000504959062	7.819935053713	-2.391783632821
	H	-3.439018264699	7.475564650843	-0.717600174944
	C	-2.649800103435	4.872530248115	0.598375048464
	O	-3.257253224648	3.779698465898	0.575538499536
	N	-2.907025268849	5.741796199860	1.571639363564
	C	-2.353399787524	7.077035284918	1.832084355576
	H	-1.970497639608	7.542897005253	0.917577506199
	H	-1.519082235917	6.989506616212	2.553307780684
	C	-3.541725282105	7.797699607805	2.438725512510
	H	-4.244823010574	8.088638242994	1.640257126554
	H	-3.255825282176	8.704708936331	2.989377197616

	C	-4.167177446674	6.726514360719	3.325093340503
	H	-5.231198612786	6.910341964350	3.536325869670
	H	-3.645062417319	6.674758074045	4.294443463786
	C	-3.958187819402	5.416256357726	2.554775686622
	H	-3.579953210617	4.601310397672	3.209119997635
	C	-5.206432879403	4.945274440041	1.790420431559
	O	-5.656597445664	5.632921367613	0.903125257479
	N	-5.689897882013	3.733188712351	2.198110056751
	H	-5.233847848723	3.344467604403	3.017299573833
	C	-6.430834932391	2.763423634729	1.406282612432
	H	-7.480003442525	2.686362811689	1.754498879752
	C	-6.423262247204	3.084674601096	-0.096805177632
	H	-6.911050691440	4.061574967158	-0.237758795040
	H	-5.370575555048	3.225887486780	-0.403376027322
	C	-7.061646122587	2.041469347046	-0.993213381910
	H	-6.515357027639	1.081841527478	-0.925988138268
	H	-8.101492170044	1.815471412646	-0.689663383939
	C	-7.023162130495	2.493340451725	-2.445712334980
	H	-7.796109165583	3.270638255151	-2.611463677711
	H	-6.049806463527	2.989721924843	-2.645683072687
	N	-7.182001772758	1.360225712900	-3.328265615233
	H	-7.296549067608	0.455942871193	-2.874268715585
	C	-6.541602261234	1.257149541446	-4.540633225060
	N	-6.466478089339	2.436852547846	-5.245229615249
	H	-7.259173331429	3.066553757012	-5.153096945723
	H	-6.120880259840	2.363464950471	-6.196290399120
	N	-6.019236052606	0.129852953541	-4.883916602608
	H	-6.504499898021	-1.152770159889	-3.843501509688
	H	-5.735566249026	0.100193816534	-5.862814962042
	C	-5.770438818064	1.400578833120	1.594972211959
	O	-4.526865292482	1.282782045995	1.701118410515
	N	-6.562710930025	0.345499226314	1.584052771720
	H	-7.565949183327	0.438916854620	1.355396854084
	C	-6.126645063873	-1.029346890373	1.405346556088
	H	-5.169689557663	-1.039351504859	0.851050654814
	C	-5.958268271723	-1.790976660349	2.735011875392
	H	-5.579426257417	-2.795743923685	2.466676390213
	C	-4.980002813570	-1.139690506992	3.688306491060
	H	-5.352836845731	-0.152441321515	4.015835531641
	H	-4.869763725333	-1.767959873649	4.584229508594
	H	-3.988409264386	-0.993012194012	3.231269304071
	O	-7.204461483984	-2.011993957314	3.327772337369
	H	-7.528215088315	-1.188812020143	3.714951438689
	C	-7.245687778415	-1.690632429944	0.581463189182
	O	-8.297902285713	-1.089107570324	0.412814089828
	N	-6.963319274608	-2.922937888024	0.158847754550
	H	-6.074926560455	-3.364704723557	0.414341148258
	C	-7.927119323403	-3.918298247868	-0.269476222520

	H	-8.897597959503	-3.701727865457	0.216458015856
	C	-8.113068716131	-3.974984667300	-1.786222807116
	H	-7.110151832173	-4.057235676674	-2.240182690816
	H	-8.636057660546	-4.911621397015	-2.040973656133
	C	-8.888057581634	-2.786616663911	-2.363872088863
	H	-9.844642298638	-3.102167417620	-2.803911954444
	H	-9.135125041584	-2.070199575835	-1.558772603966
	C	-8.175142318711	-1.988955326177	-3.422039565039
	O	-8.685900124781	-1.622135028320	-4.446590664882
	O	-6.915542136258	-1.672355055230	-3.076502654426
	C	-7.383972218371	-5.248634913181	0.266532315819
	O	-6.236428969717	-5.332136384269	0.687260024919
	N	-8.241196815900	-6.277820843709	0.210550088428
	H	-9.141990322624	-6.203269188680	-0.272761468655
	C	-7.856686201985	-7.644393889541	0.455006687317
	H	-6.882216352319	-7.849806791912	-0.035102052573
	C	-7.688218285512	-7.949402977358	1.945999765165
	H	-7.090962277593	-7.140686946972	2.403389479453
	H	-8.675638637075	-7.949778216945	2.439343205237
	O	-7.109715843038	-9.212329991340	2.152004663416
	H	-6.155011557388	-9.145141255636	2.031751123287
	C	-8.930481615851	-8.510885597808	-0.218167354152
	O	-9.790769038380	-7.992568124312	-0.907930795454
	N	-8.827486895359	-9.830066164232	0.014443522640
	H	-8.173043019647	-10.173331433335	0.715328464640
	H	-9.544691404474	-10.437020668183	-0.368775662827
	H	11.477833628309	-0.424558276623	5.469103119957
	H	11.433033641161	-2.111991259114	4.900417015995
	H	12.621992947209	-0.972501908270	4.225861340884
	C	11.579683791329	-1.059701730358	4.576231401942
	Mn	-2.686066636474	2.104094886788	1.735979903873
**(MnA5)$^{2+}$ - 5**<br><br>**E** = -6170.898252<br>**H** = -6169.013537<br>**G** = -6169.292353	N	4.512644324504	-1.189950857081	0.800471954393
	H	3.670675430804	-1.478742117454	1.340119747864
	C	4.854442502601	0.173177154899	1.224066721485
	H	5.856231306557	-0.068062335953	1.598041627869
	C	5.241541290651	0.959022072615	0.034935723342
	H	5.650724363853	0.431899198076	-0.832500438858
	H	5.511928251574	2.018449430738	0.184258526078
	C	4.270109738537	1.367593873925	-0.528645273668
	O	3.455802550121	0.658513537257	-0.923536869429
	O	3.654053185525	2.334046177016	-0.298966051867
	C	3.888484680657	0.623728338058	2.301642180206
	O	2.972588031904	-0.120083674104	2.605260091728
	N	4.064832787215	1.863044193454	2.782838611062
	H	4.826193645189	2.439725715848	2.430406150470
	C	3.023333166691	2.479898169663	3.594697837888
	H	2.838181032405	1.853457390865	4.484362884652
	C	3.449605602413	3.886010947949	4.013811166903

	H	4.387804902584	3.797778335915	4.592573498908
	H	3.685755077362	4.474629296177	3.105893558547
	C	2.392076991346	4.591564408111	4.816628225656
	C	1.723620343327	5.703711234001	4.299854176984
	H	2.012695110410	6.101962660159	3.320424671761
	C	0.718290252360	6.332634303897	5.033771521817
	H	0.216322344985	7.213947949402	4.624727107615
	C	0.370740235072	5.851068322639	6.293022759500
	H	-0.408774895112	6.349487805512	6.875780046353
	C	1.037644753302	4.745033081216	6.820001409325
	H	0.781317009001	4.374258061857	7.816152375855
	C	2.041035780075	4.120844634252	6.086343488346
	H	2.569000895669	3.260663539308	6.514086735190
	C	1.693776122344	2.489700373851	2.839203057889
	O	0.650591918965	2.195443088213	3.400852808390
	N	1.758616104828	2.855777436971	1.541795626961
	H	2.666008875190	3.033181347393	1.116618582852
	C	0.616201430401	2.721618600435	0.659151042465
	H	-0.257826767329	3.193964472479	1.148049293103
	C	0.878252120815	3.382948707314	-0.690756222089
	H	1.900979760526	3.100869001991	-1.019360069289
	H	0.205149561138	2.921172902106	-1.435973983030
	C	0.693776169218	4.898226259354	-0.756512189225
	H	-0.380945040246	5.098937601788	-0.571283371864
	C	1.489259593315	5.657305839499	0.293684168340
	H	2.572787529309	5.446915792264	0.202804214824
	H	1.366178786147	6.744276893723	0.165398871177
	H	1.183921357249	5.405750215630	1.322050932487
	C	1.035649860909	5.390984101299	-2.153609152465
	H	0.463408749480	4.859488510896	-2.930583528048
	H	0.829041864639	6.466600604583	-2.262678425601
	H	2.109377010910	5.240957308149	-2.369512110071
	C	0.341347772707	1.233024388892	0.440077487621
	O	1.246403325912	0.470185941037	0.117328394003
	N	-0.936267508340	0.854275799348	0.600025925557
	H	-1.677033264042	1.551572872449	0.704224143282
	C	-1.427800304954	-0.478561090457	0.318700763489
	H	-1.088548792367	-0.764187309952	-0.698897831028
	C	-0.869195191631	-1.547203455649	1.270314066618
	H	-0.969504496337	-2.532023313791	0.778190184309
	H	0.218611618682	-1.365546177252	1.306031477718
	C	-1.410106205279	-1.663001037585	2.692619261257
	H	-2.402185697931	-2.152633347027	2.697666686152
	H	-0.748650466429	-2.356262821525	3.236582198854
	C	-1.515498045791	-0.374459384078	3.493283145410
	H	-1.488776711426	-0.602558099238	4.574374606472
	H	-0.656125464891	0.285259494498	3.284203007661
	N	-2.768586596360	0.284178374795	3.164729675884

	H	-3.568994559811	-0.319470581136	2.915645193348
	C	-3.086534385421	1.565939050490	3.315200697460
	N	-2.162436388910	2.500217126079	3.552699039925
	H	-1.154465597636	2.304286614334	3.577948425438
	H	-2.457570553001	3.436593548213	3.801468106362
	N	-4.377738297918	1.903571238361	3.225291720695
	H	-5.038553218995	1.162112094300	2.968262319112
	H	-4.646669504384	2.868704575693	3.080086324961
	C	-2.951626466738	-0.350236819390	0.234673803517
	O	-3.486527603786	0.745692587051	0.195004898897
	N	-3.652389343474	-1.501735419267	0.180826128979
	H	-3.173085504995	-2.394235924734	0.234298722683
	C	-5.093219895870	-1.480118608022	0.031899569414
	H	-5.359584989562	-0.936894692913	-0.892983170498
	C	-5.638441889020	-2.902056791780	-0.067542260320
	H	-4.976344618007	-3.501280831055	-0.720291916860
	H	-5.654799969602	-3.382142383809	0.927254441086
	C	-7.030101450191	-2.955345813897	-0.682808154438
	O	-7.528661249704	-1.991058646861	-1.239698236047
	N	-7.621892036106	-4.164476367114	-0.637616470291
	H	-8.577867746457	-4.221878144227	-0.974475038066
	H	-7.301450975519	-4.880789826410	0.002275322122
	C	-5.761915753172	-0.768360338236	1.206729779411
	O	-5.225879605010	-0.689778765618	2.317289846153
	N	-6.988467943283	-0.305691011951	0.954799408089
	H	-7.374590775064	-0.559964971723	0.037454358872
	C	-7.955331039331	-0.141801018443	2.022282811605
	H	-7.576147144771	0.589793121319	2.760259512157
	C	-9.274746883252	0.349143983958	1.412203034448
	H	-9.012646730641	1.112922388723	0.653733352249
	H	-9.728424233927	-0.499458231227	0.864315580786
	C	-10.305306044617	0.961061013723	2.358770650499
	H	-10.600711348547	0.188493243144	3.092379551673
	C	-9.769565473699	2.189977756169	3.081260976743
	H	-9.345288015038	2.910466445126	2.357163395258
	H	-10.575180837620	2.723590600620	3.615901060071
	H	-8.977580251479	1.953610355445	3.816395291663
	C	-11.563122063641	1.303531189775	1.574801454460
	H	-12.022398548827	0.407935940396	1.121070244125
	H	-12.306888450140	1.778217027522	2.236694747223
	H	-11.345522172183	2.024726972781	0.766563948478
	C	-8.153368405733	-1.503566901726	2.701275013519
	O	-7.870347464479	-2.546409574108	2.132495566659
	N	-8.710805538612	-1.466334863629	3.931898121872
	H	-9.050767140920	-0.564552192332	4.254998049422
	C	-9.414203630734	-2.628976592260	4.443274647273
	H	-8.686989894295	-3.451278142533	4.561749686169
	C	-10.017459540263	-2.289399270532	5.824580024759

	H	-9.153557217986	-1.938684434622	6.424436875356
	C	-11.052739079108	-1.172253450049	5.773325318741
	H	-11.935840798662	-1.476254640418	5.186977691256
	H	-11.394552344049	-0.934292389706	6.792727513367
	H	-10.679050637361	-0.227195351259	5.340774173792
	C	-10.616536870438	-3.502528191791	6.518553541516
	H	-9.880033166508	-4.310446725604	6.659442486055
	H	-10.979607391850	-3.221454388917	7.519117036661
	H	-11.485154791724	-3.894749352150	5.959792437323
	C	-10.490476255597	-3.014716887099	3.420906054325
	O	-11.048574708163	-2.137121754247	2.773853027913
	N	-10.849957108433	-4.309063641487	3.281818466184
	C	-10.276350301652	-5.517634589764	3.863118303641
	H	-9.861486392949	-5.336335077522	4.862886778763
	H	-9.455976417094	-5.897989310154	3.221158216659
	C	-11.458932492671	-6.470465852541	3.876727678839
	H	-12.104116898965	-6.239697898543	4.740339853230
	H	-11.159534900947	-7.527013051482	3.940287253468
	C	-12.186174811561	-6.122281351731	2.583988011943
	H	-13.243321986375	-6.428137811077	2.583188496559
	H	-11.701389977391	-6.615580594920	1.724225353325
	C	-12.026722450837	-4.602989709794	2.459933562030
	H	-11.847961867535	-4.288595515296	1.415817203931
	C	-13.224258672319	-3.841788060087	3.032486344571
	O	-13.532633226896	-3.956384848885	4.205924633191
	N	-13.894367702757	-3.077889823663	2.140428406442
	H	-13.416269928022	-2.825873593332	1.274995644822
	C	-14.850003393283	-2.076428547564	2.562747517690
	H	-15.672056701217	-2.563310320668	3.117742048823
	C	-14.172042880625	-1.004965514173	3.440607095759
	H	-14.045979101305	-1.431149063993	4.451249009742
	H	-13.152833137181	-0.867790832994	3.031487956438
	C	-14.833340342560	0.361713224231	3.522747755801
	H	-14.823153892188	0.830136461435	2.516715480493
	H	-15.897365768549	0.300253403758	3.821678888364
	C	-14.066260824810	1.262121574586	4.481422209637
	H	-14.435474988247	1.120445549269	5.511351533657
	H	-12.996074616744	0.948569430899	4.491382225064
	N	-14.174263572951	2.668596892205	4.115025105409
	H	-13.928499688236	2.810596622125	3.134678567764
	C	-13.396230594156	3.569840019671	4.855321808981
	N	-13.701244433984	3.590214402112	6.189080282555
	H	-14.642419382495	3.319597475060	6.480928234763
	H	-13.187898014523	4.223456092829	6.787809742190
	N	-12.490855931194	4.245764882704	4.245964755652
	H	-16.077499609147	5.313743876589	5.563624212657
	H	-12.077531674280	4.944146345401	4.869144590780
	C	-15.371159161872	-1.396725819357	1.309971249963

	O	-14.625310023680	-1.136690648666	0.381837446120
	N	-16.676942600801	-1.029384292493	1.359685319922
	H	-17.181310274083	-1.144707006641	2.243623832752
	C	-17.178119599860	0.097589608946	0.603808717371
	H	-16.317034230957	0.687169656389	0.233910200747
	C	-18.031549784843	-0.298799155630	-0.605978274933
	H	-18.294238715937	0.631390134047	-1.148181856929
	C	-17.331540996270	-1.255060963384	-1.538243783608
	H	-17.127111834405	-2.210138768795	-1.026145476315
	H	-17.962479771101	-1.458770909867	-2.415421051645
	H	-16.364059302601	-0.848770128290	-1.869540592661
	O	-19.285539841773	-0.814379332803	-0.174733959659
	H	-19.148526326816	-1.594163787748	0.385654115861
	C	-18.013378420207	0.914602190840	1.592877855064
	O	-18.413315820956	0.400102948158	2.626783446567
	N	-18.264395394555	2.185646196964	1.239599899405
	H	-17.998580772205	2.559534889965	0.325664323328
	C	-19.156951675304	3.048104907830	1.983607063670
	H	-19.928224922753	2.416530808086	2.464918259842
	C	-18.427550900625	3.876113645317	3.046456174143
	H	-17.726355643742	4.562731481894	2.538928607821
	H	-19.161890645322	4.517062680955	3.565824837078
	C	-17.679552244448	3.009182809894	4.040571464590
	H	-18.328908398418	2.255232121481	4.512706237246
	H	-16.895179320035	2.429348100194	3.518610166517
	C	-16.924071074888	3.703997596765	5.125364605177
	O	-16.574919767244	3.168903696321	6.152468863535
	O	-16.619154487254	4.970490349929	4.834577282930
	C	-19.810315659004	3.969949572363	0.958052255347
	O	-19.293594501598	4.156743643108	-0.133965922141
	N	-20.944009386533	4.566539171497	1.363955097683
	H	-21.290532641205	4.484441284852	2.322239982870
	C	-21.619614478338	5.561385991280	0.576361293610
	H	-20.876818110778	6.274743794553	0.162576308524
	C	-22.367982235646	4.950514041073	-0.614242658285
	H	-21.710102941233	4.210976558684	-1.103714134885
	H	-23.258232203190	4.404922534034	-0.255844967776
	O	-22.804476629535	5.943665980213	-1.510306526927
	H	-22.067775325529	6.186984012975	-2.083277807707
	C	-22.549654976602	6.321469881051	1.526225232005
	O	-22.535896526881	6.097905463483	2.723143596716
	N	-23.348600966192	7.225860364290	0.927758133159
	H	-23.421553870917	7.237185762856	-0.088536753829
	H	-24.035897858890	7.700670842329	1.502874703798
	H	5.864864178305	-2.663661054661	1.455596172128
	H	5.094483970381	-2.977057681251	-0.139066894482
	H	6.379342153353	-1.735273700831	0.006152228109
	C	5.528770689704	-2.209675177775	0.514455964103

	Mn	-21.259181376334	0.539105561017	-0.124420675842
**(ZnA5)$^{2+}$ - 1**<br><br>**E** = -6799.291591<br>**H** = -6797.408855<br>**G** = -6797.681185	N	-14.745674065135	-1.543473105428	0.809982065787
	H	-15.004456080600	-0.646908042430	1.223203096095
	C	-13.777626998494	-1.370647091512	-0.238593008576
	H	-13.549107994887	-2.382887168412	-0.629460036634
	C	-14.191757991415	-0.543231032543	-1.475433098159
	H	-14.927097087347	-1.129363078509	-2.049066136256
	H	-14.648838068220	0.420730035362	-1.203855079387
	C	-12.935655935167	-0.291903016275	-2.252143154998
	O	-12.562682919894	0.890319067509	-2.488675172180
	O	-12.100270853128	-1.252753083943	-2.359055162993
	C	-12.442282871752	-0.946362065616	0.395920037361
	O	-11.761353833480	0.048144007412	0.086182014739
	N	-11.962730859726	-1.821526126435	1.287312099586
	H	-12.623709915711	-2.514340178797	1.644019126993
	C	-10.641200746317	-1.720731121683	1.874027145419
	H	-10.617700778454	-0.972211067162	2.687699201205
	C	-10.258891721428	-3.109782220024	2.411896183651
	H	-10.963382800053	-3.352067235295	3.228725242112
	H	-10.450194747467	-3.848516273764	1.611567125819
	C	-8.844517618435	-3.254898232429	2.899282215048
	C	-7.928932549246	-4.033890286778	2.184954165289
	H	-8.245276613737	-4.530456321045	1.259272097850
	C	-6.635390461783	-4.237378301614	2.668446198272
	H	-5.946515403776	-4.887731351436	2.121284161951
	C	-6.240727423593	-3.647045260346	3.868696289401
	H	-5.241517373346	-3.835070276749	4.273192314020
	C	-7.139880533763	-2.843536201797	4.574801337469
	H	-6.839407500407	-2.387975170619	5.522227382737
	C	-8.431173617918	-2.650699186164	4.093171304596
	H	-9.140787661490	-2.047194145279	4.670377342187
	C	-9.570622677207	-1.253542087222	0.895701075304
	O	-8.670161599630	-0.521217035143	1.266256099806
	N	-9.583377701028	-1.752431124943	-0.377743018466
	H	-10.278700730896	-2.451331170761	-0.637538038468
	C	-8.372430604025	-1.681401118047	-1.177228076158
	H	-7.525651542050	-2.120628151499	-0.610135033845
	C	-8.538624592941	-2.403065167884	-2.517119174287
	H	-9.440782689037	-2.008276143175	-3.028857207126
	H	-7.679886548480	-2.112290149008	-3.148629217125
	C	-8.605768613168	-3.933588279426	-2.484431170643
	H	-7.705407550091	-4.295218306281	-1.946577129294
	C	-9.844378715450	-4.484901319674	-1.782932118393
	H	-10.764561752838	-3.999004281929	-2.161593148789
	H	-9.952530691576	-5.562721401691	-1.978975133221
	H	-9.807932690780	-4.382324310535	-0.683899040290
	C	-8.551087616756	-4.447097315056	-3.916422272186

	H	-7.656325566481	-4.089518293502	-4.449696310905
	H	-8.539584626306	-5.547060401007	-3.944366275878
	H	-9.437166662142	-4.111297292282	-4.482806312237
	C	-8.035791586599	-0.227167013945	-1.468148094892
	O	-8.936520639534	0.591723047023	-1.789606117733
	N	-6.766545508334	0.116475010309	-1.466006096515
	H	-6.035040407407	-0.592937043133	-1.314734085796
	C	-6.196833440813	1.370685102314	-1.949538130007
	H	-6.494797486079	1.478685109316	-3.012448209108
	C	-6.688697449467	2.636418189786	-1.235821078884
	H	-6.491591465716	3.487365252862	-1.912203129893
	H	-7.788123563389	2.571019189747	-1.210722080253
	C	-6.168436413771	2.992967215450	0.153100019658
	H	-5.137601365847	3.389709242882	0.101777015907
	H	-6.778198467616	3.833603275704	0.520131045951
	C	-6.197050456403	1.884160138305	1.190443092594
	H	-6.208079462791	2.318958169854	2.206467167833
	H	-7.119637493115	1.286596096309	1.087975088677
	N	-5.021154356424	1.047907078576	1.022140083628
	H	-4.150500293378	1.505002109930	0.701397058627
	C	-4.851572341922	-0.199868013290	1.454347115196
	N	-5.883497431477	-0.947979067244	1.849843141877
	H	-6.847500472664	-0.612471044477	1.811160136806
	H	-5.735196407046	-1.845345129969	2.307423174764
	N	-3.609944257120	-0.690854048624	1.472548113757
	H	-2.857772202234	-0.095261006669	1.107585090457
	H	-3.445498241201	-1.681319122033	1.599079124838
	C	-4.682192330251	1.111450079373	-1.961465134312
	O	-4.254713301607	-0.023745001059	-1.819098123631
	N	-3.890031271427	2.177355158826	-2.162593145620
	H	-4.297829303870	3.101199224342	-2.264432153583
	C	-2.448775170563	2.034688146979	-2.252111155650
	H	-2.199192150141	1.337178096334	-3.072433212588
	C	-1.811718122464	3.388963245839	-2.555580173627
	H	-2.417815168767	3.911203282735	-3.319434229042
	H	-1.790278119922	4.019863290187	-1.649424111664
	C	-0.403902022628	3.262831234404	-3.120916215178
	O	0.049731009452	2.190402156194	-3.487949242290
	N	0.259342025755	4.425008318951	-3.254363223646
	H	1.220099095813	4.378343315383	-3.579473246852
	H	-0.046630996098	5.268012378602	-2.784461192682
	C	-1.863946128321	1.470289103707	-0.956143060380
	O	-2.454207169732	1.564762112534	0.126070017791
	N	-0.651681041295	0.929500065415	-1.086197068996
	H	-0.229040010888	1.024817074912	-2.018731135125
	C	0.282687025647	0.901011062075	0.022882010282
	H	-0.130896004233	0.287437019591	0.845434068037
	C	1.600138119640	0.304347019873	-0.488547026701

	H	1.334302102654	-0.596062046459	-1.076913066921
	H	2.034655152778	1.029427071309	-1.204428077804
	C	2.664925195735	-0.093509009279	0.532160046524
	H	2.963633216587	0.812911056568	1.092341086737
	C	2.177924161955	-1.160906085464	1.502077114436
	H	1.897388140752	-2.083647149797	0.961885078041
	H	2.966881216581	-1.430799103331	2.225777168447
	H	1.298808099605	-0.846511060158	2.095344157794
	C	3.902474287050	-0.569453045840	-0.216167006999
	H	4.358317316784	0.246396014456	-0.803645051811
	H	4.663282340011	-0.959358071465	0.480453043186
	H	3.654174267452	-1.391881101227	-0.910738054301
	C	0.484058040969	2.342790164689	0.513563045577
	O	0.210779021619	3.300936235042	-0.192009005111
	N	1.013096079310	2.463952175152	1.749761134675
	H	1.347665103508	1.612365114527	2.192198167902
	C	1.674315127761	3.692263265770	2.152652164698
	H	0.923137074128	4.501585320524	2.172370166263
	C	2.247194167655	3.502426251214	3.576227268315
	H	1.377529105136	3.173045227848	4.180177310225
	C	3.324352243642	2.426187170897	3.653183271926
	H	4.213054309523	2.719400194027	3.069914228339
	H	3.639969266218	2.293336164181	4.700231347657
	H	3.002560219996	1.432764102501	3.292248245302
	C	2.782056209087	4.791184343508	4.179667310359
	H	2.018289150412	5.584758406131	4.219119310517
	H	3.111155228606	4.611403331841	5.214753382003
	H	3.662268271605	5.155853366512	3.621250266630
	C	2.766897207109	4.007188288549	1.122288089817
	O	3.351431248910	3.086048218232	0.565838050840
	N	3.112160231454	5.291301379371	0.885441074586
	C	2.506276189577	6.535279492709	1.349734107175
	H	2.050107154907	6.427048457503	2.341891177211
	H	1.712041131653	6.857942515648	0.646532056702
	C	3.679466272901	7.499828538982	1.339439106674
	H	4.290335317925	7.343359504959	2.243789171354
	H	3.368522250566	8.554487633499	1.310391101655
	C	4.460798327878	7.063670490153	0.106716016088
	H	5.514568388741	7.380821549948	0.122834017420
	H	4.006433295492	7.484777520913	-0.806332049836
	C	4.320581316734	5.537533411322	0.093226015340
	H	4.191103306970	5.141922367966	-0.930185058905
	C	5.498770384067	4.835614345798	0.773467062862
	O	5.718819429544	4.998761358573	1.961697152076
	N	6.247444482085	4.062346290624	-0.042536994456
	H	5.847467445960	3.783569270472	-0.939864060046
	C	7.168671533267	3.068478215913	0.470563042671
	H	7.989085555919	3.562750250931	1.021686081299

	C	6.426494461841	2.076065145799	1.383622110191
	H	6.278268474744	2.571247180473	2.359068180754
	H	5.418661415405	1.949053139058	0.945966076989
	C	7.027286485056	0.696808044107	1.584601121061
	H	7.036302501189	0.147890006112	0.620867055378
	H	8.077632573323	0.727614050032	1.930290148967
	C	6.170751458400	-0.073133009143	2.573729196399
	H	6.360215487664	0.331965019686	3.590552265782
	H	5.100529372117	0.139601006492	2.357010178682
	N	6.425800445879	-1.488428109154	2.514082189012
	H	7.002739487089	-1.850494135729	1.756286133576
	C	5.678250426174	-2.440982178304	3.149374235190
	N	4.849496353115	-1.953507144587	4.146583307505
	H	5.184178377822	-1.137045086423	4.650356344312
	H	4.480287327988	-2.665656195895	4.767282349106
	N	5.744994441318	-3.671139266713	2.770807207842
	H	7.224768502161	-3.976520292051	1.952322147389
	H	5.232065378661	-4.300225312618	3.385863251429
	C	7.708464554141	2.313612161987	-0.733662043269
	O	6.989707491896	2.083626146752	-1.692572113275
	N	8.981072654223	1.872640129515	-0.613406037175
	H	9.449134693558	1.943245132995	0.294330030048
	C	9.496376674956	0.781237051786	-1.410669094944
	H	8.641874641456	0.239222011938	-1.860312125275
	C	10.433012732616	1.250072081496	-2.536289175768
	H	10.732979766687	0.349331019412	-3.105645212835
	C	9.770022697250	2.231951153496	-3.475306240001
	H	9.511972692674	3.161449223912	-2.938988204410
	H	10.459465774117	2.490662174541	-4.292375301511
	H	8.838076631713	1.828454125563	-3.899090272628
	O	11.622590858538	1.758192118900	-1.993159136702
	H	11.411427810344	2.578388178735	-1.528467099176
	C	10.255804765946	-0.131559015171	-0.444646023327
	O	10.515229784686	0.240569011459	0.690925058919
	N	10.595950746837	-1.328649103190	-0.949923058078
	H	10.418584767840	-1.548298117126	-1.931989130734
	C	11.491728817431	-2.275403167437	-0.320610014712
	H	12.185814895480	-1.721164131550	0.339236033094
	C	10.739478751235	-3.336608248025	0.491111043934
	H	9.898459730697	-3.691061271399	-0.129296000836
	H	11.393409801275	-4.210536309544	0.658499056223
	C	10.230097758532	-2.812021209486	1.826705141837
	H	11.023701809629	-2.805133205575	2.589705195133
	H	9.912744711781	-1.757965133611	1.713483134364
	C	9.038689631847	-3.530025257798	2.403151180354
	O	8.918365626476	-3.823167281217	3.564722266992
	O	8.085335594866	-3.722030274500	1.486898114849
	C	12.274341909984	-2.932650216748	-1.455391097240

	O	11.871194837549	-2.889243214624	-2.608554176527
	N	13.399590974055	-3.573060264191	-1.084688067153
	H	13.641645008749	-3.716433272573	-0.101071998879
	C	14.145103026339	-4.404480326938	-1.991336136955
	H	13.445663949993	-5.035398368605	-2.579376179118
	C	14.958696060087	-3.583889268038	-2.997114208213
	H	14.314097050833	-2.780074207452	-3.393024236716
	H	15.811005130150	-3.104449229918	-2.485226169500
	O	15.478826124414	-4.392109321755	-4.024044282589
	H	14.783573051778	-4.551229332707	-4.673531328551
	C	15.018076097552	-5.323265415620	-1.131051074557
	O	14.901281069419	-5.335176401729	0.080887014268
	N	15.885974147276	-6.087344445451	-1.821285123241
	H	16.033359165889	-5.913986454938	-2.814235191843
	H	16.528693201435	-6.666966468231	-1.292587086574
	H	-16.562334163344	-2.452184172735	1.327918103030
	H	-16.545776207223	-1.841945129102	-0.345838016173
	H	-15.622062128806	-3.305916232172	0.089899015004
	C	-15.924961122343	-2.307048162723	0.444550040629
	Zn	-10.807863797946	0.233997020559	-1.755377118190
**$(ZnA5)^{2+}$ - 2**<br><br>**E** = -6799.286153<br>**H** = -6797.402716<br>**G** = -6797.672211	N	-14.784411056870	-0.695743133103	0.527088054046
	H	-14.621376097377	0.271407938558	0.246520033495
	C	-13.839452999039	-1.570015191584	-0.107484991595
	H	-14.062889996017	-2.604192263716	0.214475031539
	C	-13.804244984542	-1.552125185200	-1.662604101859
	H	-14.544986027667	-2.271503242239	-2.045275131611
	H	-14.075073032150	-0.551163119042	-2.034017129060
	C	-12.413207915736	-1.925949207449	-2.117643136722
	O	-11.752495882592	-0.998104133297	-2.756080183835
	O	-11.904573856719	-2.967836275680	-1.715414107438
	C	-12.478499921427	-1.204491155732	0.448839048162
	O	-11.916721861318	-0.122509073484	0.137886026109
	N	-11.864435868218	-2.081556211733	1.237905105893
	H	-12.353502910523	-2.952781278198	1.434609121314
	C	-10.539053767778	-1.914740196128	1.806175144242
	H	-10.568355793751	-1.187249142764	2.637517206263
	C	-10.063747715245	-3.286366292009	2.313336180177
	H	-10.796227772336	-3.622344319426	3.071281236051
	H	-10.131325713733	-4.004358343873	1.473997122776
	C	-8.679503641866	-3.314816284402	2.900611222541
	C	-7.683424521731	-4.108877332972	2.325813184855
	H	-7.912249548541	-4.707667378110	1.436802121794
	C	-6.416930429749	-4.197497334502	2.908532222777
	H	-5.663903372599	-4.859494379344	2.470644192977
	C	-6.131778453613	-3.477872278691	4.067568306969
	H	-5.152232365350	-3.568523283670	4.546324341161
	C	-7.115393491578	-2.665463226120	4.637740351230
	H	-6.902470505689	-2.110287186662	5.555228410599

	C	-8.378366596898	-2.588020231271	4.060025308627
	H	-9.151442645399	-1.973755191251	4.535384342386
	C	-9.495595715172	-1.357562146843	0.851908075419
	O	-8.624285636383	-0.620166091319	1.268531106884
	N	-9.509077669781	-1.741210175383	-0.475783017979
	H	-10.136991720374	-2.501809235081	-0.790342038489
	C	-8.271126604844	-1.541540153232	-1.234233070877
	H	-7.416375540530	-1.950616180482	-0.657644032792
	C	-8.349757581450	-2.209955202627	-2.613034169950
	H	-9.370603678535	-2.069318199376	-3.022209200972
	H	-7.671120568597	-1.668221159603	-3.296519219003
	C	-7.968008584844	-3.692301308322	-2.643732176090
	H	-6.905232493422	-3.759867305071	-2.331354152203
	C	-8.798249640681	-4.552005371143	-1.701595103964
	H	-9.876303714294	-4.464340375794	-1.928226124857
	H	-8.526135586523	-5.613534429919	-1.808460115728
	H	-8.639373591024	-4.291546355934	-0.639505032636
	C	-8.083478553146	-4.200636345150	-4.071866279294
	H	-7.478773528763	-3.603380300189	-4.772668325730
	H	-7.750074554996	-5.246574418684	-4.149474282193
	H	-9.132425655519	-4.160546347734	-4.412926302755
	C	-8.039801585956	-0.048092045116	-1.413965086023
	O	-9.010509673312	0.735204007563	-1.600884100808
	N	-6.793541485691	0.367100991992	-1.442990087706
	H	-6.020072470165	-0.312878051936	-1.396286083775
	C	-6.306141457557	1.688836090147	-1.825065115707
	H	-6.648381498657	1.881203101130	-2.862348188410
	C	-6.830439533568	2.852117170730	-0.974480052203
	H	-6.692351524207	3.775115240578	-1.565495097109
	H	-7.924780624551	2.731817152963	-0.925818050116
	C	-6.280921504882	3.091139189123	0.428715046994
	H	-5.268348416739	3.533346231334	0.388831043993
	H	-6.910631530141	3.865389243809	0.894722081518
	C	-6.233967501743	1.885927103474	1.352133111066
	H	-6.229179453076	2.219414129534	2.406011187590
	H	-7.137373556545	1.265391053757	1.216009106023
	N	-5.035365384987	1.116221056782	1.069365094312
	H	-4.195975330774	1.631064100631	0.749872070641
	C	-4.798631359682	-0.150418033470	1.404759116424
	N	-5.784316438501	-0.961165095351	1.800299144194
	H	-6.754197474735	-0.649755079531	1.844996148157
	H	-5.586680430817	-1.874436162390	2.204352173371
	N	-3.543607269240	-0.594400059474	1.322819109812
	H	-2.830298219067	0.057817993540	0.974010084597
	H	-3.332385249533	-1.581696128468	1.391597118256
	C	-4.781886371153	1.507169085472	-1.909477120920
	O	-4.300210331611	0.384763008179	-1.891833118456
	N	-4.040358325040	2.621280169685	-2.021808131492

	H	-4.484882359253	3.533587234529	-2.032623128017
	C	-2.597495218707	2.538614174221	-2.157549139209
	H	-2.346030195274	1.962595134117	-3.066536204203
	C	-1.998130185445	3.936358278903	-2.288813148647
	H	-2.627319235702	4.540843319011	-2.968818195816
	H	-1.974797183589	4.442937315115	-1.307733076084
	C	-0.597380084109	3.913599286191	-2.886503193038
	O	-0.138462044541	2.911887217119	-3.411288228537
	N	0.047580955886	5.094288372061	-2.864469189033
	H	1.006279022193	5.103313378916	-3.198876216200
	H	-0.248965069986	5.844809441556	-2.252716148123
	C	-1.970769171038	1.829823128273	-0.957014054381
	O	-2.525381208092	1.800191123242	0.148518026791
	N	-0.763651078898	1.313863097087	-1.185549070892
	H	-0.365253052650	1.527102112921	-2.108914135746
	C	0.199447990491	1.153438092704	-0.112589992034
	H	-0.194795033896	0.449029037082	0.643802063025
	C	1.504089088671	0.617135059288	-0.716776034296
	H	1.219028072812	-0.166061998351	-1.446680088289
	H	1.967122113188	1.438402120957	-1.297564077977
	C	2.535219163495	0.016284022438	0.235713032838
	H	2.861846184413	0.807096081240	0.935453081920
	C	1.985517134588	-1.173239063970	1.011443088338
	H	1.533968103058	-1.910564119783	0.321496038935
	H	2.789080195676	-1.703330101535	1.553331127233
	H	1.210608074306	-0.892056049495	1.749509140469
	C	3.771074257892	-0.380200998633	-0.557481024427
	H	4.239526284985	0.489345066739	-1.050573057681
	H	4.517051314100	-0.844925025983	0.108225023753
	H	3.524672243624	-1.125068053702	-1.335356080856
	C	0.426809998314	2.529760192162	0.528626054273
	O	0.139704971830	3.560331265307	-0.060814988361
	N	1.008477039273	2.519414194849	1.747720140359
	H	1.358070072334	1.624360128692	2.080051168136
	C	1.738257085870	3.689674281954	2.205629173325
	H	1.023700029365	4.524477337812	2.311583179996
	C	2.367215134383	3.389146261790	3.584286272685
	H	1.512017074046	3.072525236511	4.214945319131
	C	3.385320215028	2.255657188271	3.550435269743
	H	4.260017276925	2.524248213143	2.934725226577
	H	3.746341238247	2.049518175337	4.570123347086
	H	2.988214187733	1.301111116035	3.161565246024
	C	2.998581172216	4.617272357333	4.221040319183
	H	2.277650112245	5.441589395578	4.346756328507
	H	3.377796198273	4.367094340596	5.223780394439
	H	3.861570229813	4.974460385693	3.631010279517
	C	2.795858162470	4.024070312817	1.146118100238
	O	3.323456203681	3.114848250791	0.517375053266

	N	3.170860180902	5.306447421798	0.953327085984
	C	2.627681134731	6.543477506651	1.503233123304
	H	2.225858106107	6.402935490716	2.514919199251
	H	1.803288069591	6.916099518655	0.861455078587
	C	3.826255215677	7.475533588139	1.465337121221
	H	4.482030263218	7.261585533001	2.325258183756
	H	3.545997187158	8.538647665069	1.499112123727
	C	4.524865267070	7.072434564998	0.172865028567
	H	5.586988359058	7.359005582845	0.144632026364
	H	4.034441229087	7.546751585252	-0.694411035214
	C	4.337425261407	5.552960409827	0.101115023106
	H	4.133967250921	5.208191404195	-0.928870053441
	C	5.532078334733	4.789153382394	0.677508066110
	O	5.860364395120	4.935799394395	1.842281146071
	N	6.172815408424	3.987711327221	-0.201119998506
	H	5.677861375060	3.718420306684	-1.051993057202
	C	7.124333464940	2.984599261526	0.228241032619
	H	7.969993528705	3.474997301031	0.743152069923
	C	6.454733423639	1.955088183329	1.160055099654
	H	6.365588435944	2.415377217431	2.159725168975
	H	5.421166381748	1.820123171713	0.786456072987
	C	7.099165477471	0.581961092056	1.267199109539
	H	7.044790504263	0.078550053763	0.279823036353
	H	8.174939590907	0.638838099966	1.521630123827
	C	6.357293423812	-0.271325975352	2.287280180366
	H	6.776326450180	-0.109512961271	3.294859253474
	H	5.295459373729	0.066986042297	2.337275185235
	N	6.415401477924	-1.690777075253	1.960130157698
	H	6.113266457582	-1.854168091658	0.999147088737
	C	5.654559425325	-2.546969142134	2.769317214045
	N	6.051882425875	-2.560755140823	4.077801309064
	H	7.017098500070	-2.310773120755	4.304402325607
	H	5.550076393820	-3.151387188403	4.727471355103
	N	4.681601339807	-3.194763196616	2.237503175357
	H	8.334071603600	-4.317092255335	3.323081255141
	H	4.286081314938	-3.864983248440	2.901985225977
	C	7.596222514141	2.257784216223	-1.019158058703
	O	6.810204483496	1.976804188849	-1.908719122137
	N	8.897221608644	1.884031193685	-1.006454058152
	H	9.443338668068	2.032088209195	-0.153211995152
	C	9.379692668210	0.747497117856	-1.762104109339
	H	8.508759575293	0.143428067158	-2.082850134775
	C	10.192047691784	1.135671146489	-3.007323201537
	H	10.444546732853	0.193763082168	-3.531765240218
	C	9.417114654481	2.035479208757	-3.943120269835
	H	9.198010613735	2.998446274352	-3.450710234929
	H	10.013175674337	2.239520224402	-4.844662334821
	H	8.453266556393	1.591220170635	-4.234046289440

	O 11.423269791740 1.692589195117 -2.633054172971<br>H 11.248048779804 2.524574255121 -2.173896138820<br>C 10.253340705004 -0.044544936043 -0.788747040051<br>O 10.671548744040 0.476910104135 0.233871032981<br>N 10.497663745905 -1.324960028667 -1.125941063868<br>H 10.221146725365 -1.708624054236 -2.031205128785<br>C 11.385376837718 -2.175058085057 -0.365851010241<br>H 12.162668841362 -1.540166033378 0.099027023035<br>C 10.656175774533 -2.966071141465 0.727543066195<br>H 9.949889726679 -3.665015198954 0.244206033462<br>H 11.388279798270 -3.594454185979 1.265312105803<br>C 9.915551732498 -2.068109082280 1.699884136332<br>H 10.563912768651 -1.288016021746 2.128068166657<br>H 9.112648661275 -1.520972049583 1.170280100589<br>C 9.196035671324 -2.732160133905 2.827105221756<br>O 8.909398612156 -2.178945098330 3.864007292132<br>O 8.849396632670 -3.994566231095 2.566004198125<br>C 12.021726873262 -3.144772149992 -1.356154080268<br>O 11.517454822308 -3.348796168464 -2.449930158119<br>N 13.131152955493 -3.769387187905 -0.917099050589<br>H 13.456624957104 -3.681693177767 0.048605019487<br>C 13.740628989035 -4.855673259222 -1.636393100740<br>H 12.953701937712 -5.549273314427 -2.000327128296<br>C 14.517087062252 -4.375440222536 -2.866837188430<br>H 13.893205024789 -3.643770171769 -3.408851229719<br>H 15.436825114490 -3.855246180680 -2.547568165489<br>O 14.898554113219 -5.450280285210 -3.689529250345<br>H 14.147900045607 -5.699306335994 -4.241752290250<br>C 14.623022050292 -5.606333332997 -0.633931028752<br>O 14.609638046393 -5.311404278007 0.547662055904<br>N 15.377635104576 -6.585477352385 -1.167222069936<br>H 15.451769132920 -6.671515381699 -2.179711140607<br>H 16.026713175901 -7.067818393101 -0.555090021696<br>H -16.813965247890 -0.369328120700 0.921372082790<br>H -16.527783175320 -1.115059174047 -0.674343030525<br>H -16.337557183736 -2.071294242476 0.821993076979<br>C -16.174125182553 -1.076690166880 0.375790043261<br>Zn -10.769202796065 -0.032184060212 -1.489410092792
$(ZnA5)^{2+}$ - **3**<br><br>**E** = -6799.225088<br>**H** = -6797.343478<br>**G** = -6797.612305	N -11.871262889743 1.792557132872 -0.392284981251<br>H -11.163608812865 2.047398153692 -1.078160030774<br>C -11.834477874127 0.392871033942 -0.109366961268<br>H -12.622088939962 0.165671017329 0.636438090638<br>C -12.087669872804 -0.556939035797 -1.315258045358<br>H -12.970582956544 -0.181147007592 -1.856924089105<br>H -11.221651831093 -0.529086032072 -2.003062096239<br>C -12.427149884127 -1.955128136211 -0.837533012493<br>O -13.442822956096 -2.170344152955 -0.235281970342<br>O -11.518637850966 -2.910319201429 -1.041635026399

	C	-10.483464756125	0.000182006238	0.446285078946
	O	-9.454224690763	0.636768053820	0.189701060454
	N	-10.433541751112	-1.167934077924	1.117251126756
	H	-11.298609813826	-1.657699115603	1.342335145588
	C	-9.172441668665	-1.795160124009	1.424476149595
	H	-8.603327624354	-1.109282071935	2.085305194413
	C	-9.366384707081	-3.119695218053	2.175149204087
	H	-10.208955751573	-2.987545207147	2.876524254232
	H	-9.669518708234	-3.903059276624	1.456946153379
	C	-8.138436573847	-3.514776248061	2.950880256557
	C	-7.215468529516	-4.440202310130	2.452940224146
	H	-7.409452526252	-4.929121346849	1.492036156057
	C	-6.060778424608	-4.749754331711	3.169261277136
	H	-5.359203377213	-5.489933388598	2.774482248584
	C	-5.814098428239	-4.140946289194	4.400975363764
	H	-4.926656359847	-4.414062308723	4.979898406899
	C	-6.733684500772	-3.223820224106	4.914569401135
	H	-6.573281457245	-2.775482192497	5.900000459000
	C	-7.884399584939	-2.913526200770	4.189940350005
	H	-8.614735627488	-2.209339150349	4.606655378935
	C	-8.307020591808	-1.974473136608	0.179474059758
	O	-7.016672512384	-2.015050135847	0.461948079921
	N	-8.845052669551	-2.028343137135	-0.983039023238
	H	-10.621924777091	-2.532513178590	-1.201893038317
	C	-8.071503589507	-2.142198146433	-2.201195112487
	H	-7.079544537598	-2.637198181175	-2.018697098445
	C	-8.778569644626	-2.978251208913	-3.263096188161
	H	-9.862555742283	-2.757516190968	-3.216029184512
	H	-8.437030604627	-2.643569184206	-4.256684259314
	C	-8.550020636669	-4.486105314335	-3.147861179849
	H	-7.475083522888	-4.664053330096	-3.362714196876
	C	-8.851875669329	-5.050719357031	-1.766552082247
	H	-9.899838713690	-4.857789343789	-1.475571059959
	H	-8.705671615582	-6.142502442234	-1.759380079490
	H	-8.195154585161	-4.631316324639	-0.983180022516
	C	-9.372277677844	-5.199457365451	-4.208506258614
	H	-9.165595649677	-4.811253337759	-5.218670330833
	H	-9.167470683423	-6.280969448341	-4.215395257378
	H	-10.451059740599	-5.068705356168	-4.011966240514
	C	-7.638462553428	-0.773580047496	-2.735006149694
	O	-7.336096547217	-0.526070030792	-3.864198229867
	N	-7.402761556198	0.194100021494	-1.685475073767
	H	-8.236390616454	0.329249030685	-1.069965027851
	C	-6.739575513579	1.439734113011	-2.068516104253
	H	-7.077520540456	1.735006133067	-3.080997173764
	C	-7.089872542884	2.558873190730	-1.073256028788
	H	-6.923098522785	3.528478261028	-1.572532064416
	H	-8.181249606260	2.504634189616	-0.916772019050

	C	-6.385875451221	2.630675198439	0.285529067315
	H	-5.331834382813	2.939969219653	0.160373058227
	H	-6.861886526602	3.467154259188	0.821840107712
	C	-6.425610471247	1.438468109360	1.237038133639
	H	-6.294788435939	1.800894135218	2.270252209013
	H	-7.391603536396	0.904005072865	1.193181135057
	N	-5.285402386571	0.487778043257	0.941388116982
	H	-4.513149335695	1.022841079438	0.482755081696
	C	-4.747805350736	-0.192498005636	2.037217190888
	N	-5.496368395616	-1.083845071920	2.645284237086
	H	-6.312111476527	-1.470128096237	2.136059198210
	H	-5.192563381837	-1.615233107047	3.464018296601
	N	-3.510426262904	0.118191017296	2.381520218910
	H	-3.003730225602	0.821322069644	1.826624177718
	H	-3.011999223380	-0.398087019783	3.101648268280
	C	-5.236997384707	1.155200088898	-2.178053111867
	O	-4.773314350608	0.024211009944	-1.894916089233
	N	-4.423828327771	2.145878161442	-2.502174134545
	H	-4.825667358715	3.047790227159	-2.749412153614
	C	-2.972107222923	2.002500152335	-2.510316135075
	H	-2.687788203527	1.259808101486	-3.277653187517
	C	-2.326372175950	3.342327251767	-2.864641161056
	H	-2.919713219454	3.836355283155	-3.656403214942
	H	-2.304511175864	4.009162299608	-1.984630095933
	C	-0.908912075285	3.178136235781	-3.400288199895
	O	-0.426852039859	2.076136161386	-3.614332210744
	N	-0.265683029198	4.325041323132	-3.662876216888
	H	0.688131040087	4.267959317247	-4.007393244189
	H	-0.638850054080	5.231989383516	-3.411355201210
	C	-2.439781184079	1.517128116630	-1.154415038574
	O	-3.073436231021	1.679814131353	-0.097083960378
	N	-1.231781096343	0.972970081966	-1.217277041170
	H	-0.778533067170	1.024480084256	-2.142636109400
	C	-0.326990032277	0.981348079673	-0.082769959214
	H	-0.751554060527	0.370893036470	0.736916102343
	C	1.008934063204	0.384839037720	-0.543412990141
	H	0.764765044633	-0.531104025698	-1.116953035920
	H	1.459798095953	1.095745088185	-1.262869042614
	C	2.043769140787	0.015269011684	0.517873084201
	H	2.341371160021	0.940421077310	1.047643119672
	C	1.523038100441	-1.007842063617	1.518007154126
	H	1.244405080002	-1.948942128255	1.009643118384
	H	2.292802155543	-1.255303077979	2.269720211249
	H	0.635271037841	-0.660146035743	2.081348197448
	C	3.290565228812	-0.505799025243	-0.184108966645
	H	3.770370261647	0.280264031506	-0.792501012872
	H	4.030246282281	-0.878840054080	0.543087085748
	H	3.045116209852	-1.351216083936	-0.851170016035

	C	-0.173640021878	2.440411185469	0.380604074002
	O	-0.480319044394	3.370225254080	-0.346348978082
	N	0.323346013492	2.599238196271	1.627962164141
	H	0.741272046035	1.771988140206	2.048322192421
	C	0.955389056885	3.855917288559	2.006326193805
	H	0.187482003286	4.648930343921	1.988291187563
	C	1.505083100508	3.714907278848	3.445069295414
	H	0.629779035264	3.385715251136	4.041536337098
	C	2.600281178170	2.661614202481	3.572336302088
	H	3.500518244433	2.960874223997	3.010063265457
	H	2.889323199765	2.556330192417	4.629945379848
	H	2.309825157923	1.652945130076	3.225918279604
	C	2.006449132264	5.027804373450	4.024639337113
	H	1.230740077692	5.810503409681	4.026690338170
	H	2.315249155932	4.880106362370	5.070879412103
	H	2.894200197835	5.389024420233	3.476214297577
	C	2.059492139877	4.154747311225	0.982708118121
	O	2.648540180031	3.219133243002	0.454369079533
	N	2.403020161359	5.432323396113	0.722140097690
	C	1.782470114904	6.684895469686	1.144993127616
	H	1.305110082047	6.597373468613	2.129149201692
	H	1.003045061271	6.986395530247	0.416666076859
	C	2.951021202917	7.655043581677	1.136253130336
	H	3.541756244265	7.527342527949	2.058131196536
	H	2.634800177429	8.706479616324	1.072825125046
	C	3.761868261835	7.192784552972	-0.067667958092
	H	4.813707333750	7.514811557775	-0.034888955700
	H	3.327492227976	7.590670545197	-1.000510027076
	C	3.628425248655	5.666152428825	-0.049052956757
	H	3.522587242929	5.246213387506	-1.065403027829
	C	4.791771333372	4.984211370815	0.676312093777
	O	4.970733347994	5.164074383789	1.869168181709
	N	5.570523379380	4.207999312101	-0.106214960906
	H	5.204637365122	3.918129292156	-1.014717028601
	C	6.491239457010	3.231108243683	0.440152078675
	H	7.305793529559	3.740398281401	0.986300120369
	C	5.745182405077	2.256298175381	1.369730143758
	H	5.595985377728	2.766509209058	2.337258214521
	H	4.738122333140	2.124294164478	0.931167111335
	C	6.341615436538	0.878313072917	1.591963161951
	H	6.350593457745	0.316931034774	0.635506093083
	H	7.391991529155	0.909396076673	1.936634185124
	C	5.482512365611	0.122410020499	2.590108232401
	H	5.688536414787	0.520697049174	3.606008305535
	H	4.413186308705	0.352217036768	2.385716218779
	N	5.712213392439	-1.297171079201	2.520779228729
	H	6.273127464853	-1.662776105632	1.752434173004
	C	4.941300345360	-2.240779147661	3.142056274765

	N	4.155217289415	-1.751538117169	4.175067347162
	H	4.555116319522	-0.981227056836	4.704712383559
	H	3.782282263777	-2.473685168248	4.782750390599
	N	4.946881350259	-3.457663237681	2.719701242964
	H	6.450629442402	-3.798348259685	1.909283182750
	H	4.434359309575	-4.088296280356	3.334166285525
	C	7.043795492282	2.451941190418	-0.744125004630
	O	6.334167425512	2.209643173262	-1.707527075090
	N	8.311701587917	2.008538154706	-0.601118996545
	H	8.769006617971	2.091644161052	0.311057069413
	C	8.836327647987	0.905164076876	-1.376146052770
	H	7.987948565681	0.366292038731	-1.840583085072
	C	9.801498689198	1.357754111182	-2.484142134030
	H	10.109822702056	0.449972045756	-3.037624171278
	C	9.164099665594	2.332545182021	-3.447811200871
	H	8.899748641679	3.269456247806	-2.927467163724
	H	9.871308704405	2.578591197586	-4.253456256928
	H	8.239164563593	1.928726149732	-3.886658232140
	O	10.980153802935	1.865330149082	-1.918214091306
	H	10.770357778328	2.706988209358	-1.493260058365
	C	9.563163659293	-0.006689987319	-0.383693980705
	O	9.783263717457	0.367444039610	0.759850099262
	N	9.918141684708	-1.204205071928	-0.876032017343
	H	9.777496690880	-1.422401086848	-1.864737089555
	C	10.794672784989	-2.152364138813	-0.221500969204
	H	11.473625822496	-1.599903101440	0.455376079496
	C	10.018439717939	-3.211475217401	0.570008089273
	H	9.193816672366	-3.563079245718	-0.073516958458
	H	10.664905770363	-4.087454279977	0.753816102763
	C	9.474972672342	-2.687015181401	1.892691184657
	H	10.241730714914	-2.702775180565	2.682404237776
	H	9.182946630823	-1.625589105810	1.778319174478
	C	8.250833609864	-3.383163230352	2.423252221579
	O	8.081095562158	-3.679051249768	3.577721305026
	O	7.324156509277	-3.550673240752	1.473011154434
	C	11.603945827347	-2.813484188783	-1.336253046876
	O	11.235652802747	-2.758349184751	-2.500526133714
	N	12.707357915979	-3.471106234260	-0.933698017947
	H	12.912993919478	-3.624410248573	0.056930051050
	C	13.463740962325	-4.320293296140	-1.815059083026
	H	12.769446917617	-4.929062338294	-2.431683126428
	C	14.339204024165	-3.520993239345	-2.785103153478
	H	13.732965973292	-2.699998180073	-3.205995184813
	H	15.181593084601	-3.063882205670	-2.237608116497
	O	14.880254052677	-4.342113295574	-3.790198226245
	H	14.209764993597	-4.483177307823	-4.469239276511
	C	14.274164012680	-5.266461365377	-0.922881021132
	O	14.104898996518	-5.278018363657	0.283106067352

	N	15.148505059999	-6.052588401243	-1.578221069099
	H	15.342205100223	-5.881847403897	-2.563646136678
	H	15.750729142685	-6.652946446196	-1.025402027443
	H	-13.099828943251	3.420489250328	-0.855675013083
	H	-13.653608965500	1.906782142902	-1.615120069495
	H	-13.860687982440	2.166010159535	0.134897056261
	C	-13.172815979908	2.333212172901	-0.710048005959
	Zn	-5.974603444222	-0.830292052648	-0.566036995027
**$(ZnA5)^{2+}$ - 4**<br><br>**E** = -6799.256405<br>**H** = -6797.374793<br>**G** = -6797.625475	N	-7.548972595084	2.152687222690	-3.654215262813
	H	-6.757635539321	1.781873187092	-4.178828305123
	C	-7.483146596631	1.660265186044	-2.290377169049
	H	-8.377436620801	2.032607220949	-1.757055128663
	C	-7.422060597551	0.140313077453	-2.145551156361
	H	-8.260048649560	-0.328709948826	-2.689138198190
	H	-6.497247523824	-0.240704958152	-2.622367189714
	C	-7.419408572850	-0.380232959880	-0.707892053179
	O	-7.403643588836	-1.612747048207	-0.550301041677
	O	-7.391442560677	0.485454102100	0.212476012621
	C	-6.249333486111	2.290601222696	-1.673326121941
	O	-5.141026396798	2.165816200198	-2.186897161269
	N	-6.421914485669	3.015643275502	-0.532578038341
	H	-7.257003545955	2.775937263764	0.002478997716
	C	-5.259703396192	3.480088298381	0.177489010061
	H	-4.652254344351	4.087752333770	-0.520118039832
	C	-5.644181412788	4.362997365666	1.370307095129
	H	-6.403213467525	5.082013423132	1.018280068978
	H	-6.123338446915	3.736822323807	2.145825153756
	C	-4.455610321755	5.093398407056	1.929614135615
	C	-3.685464269472	4.544237361342	2.960397213041
	H	-3.987956299245	3.589334295551	3.404413242386
	C	-2.541223182103	5.191506397908	3.419492243349
	H	-1.961079147134	4.752022358942	4.236273300141
	C	-2.149703144557	6.407728460731	2.857066205699
	H	-1.270501075029	6.934487528456	3.241675230707
	C	-2.916211193213	6.973252513667	1.835998129342
	H	-2.642475163161	7.946207593458	1.415660098961
	C	-4.057764282411	6.316123486758	1.376732095406
	H	-4.663321319207	6.765667550360	0.581118038614
	C	-4.339179335818	2.345392208599	0.617448041172
	O	-3.103958248683	2.534814210605	0.680757045082
	N	-4.903926387216	1.189735127520	0.930050065788
	H	-5.924769446823	1.008929125981	0.739059053239
	C	-4.104599342380	-0.020715964239	1.100915075822
	H	-3.205008274535	0.234611046088	1.693388120468
	C	-4.885167408288	-1.132377039281	1.783456125581
	H	-5.889403472918	-1.213238032316	1.321413091153
	H	-4.374624379388	-2.085128110538	1.549290107505
	C	-5.014046415864	-1.024512030686	3.300218236767

	H	-3.988665343841	-1.042925038821	3.730667267428
	C	-5.698971472631	0.261283070659	3.744417266188
	H	-6.670518517597	0.374590087282	3.229668230678
	H	-5.891268452297	0.248428071497	4.829684343212
	H	-5.095653400651	1.159060130007	3.526089253295
	C	-5.776350468783	-2.236697106112	3.813581272394
	H	-5.312134436776	-3.187614181318	3.502547247592
	H	-5.849689514146	-2.233399109452	4.912863349560
	H	-6.804303575075	-2.237238099889	3.408229245364
	C	-3.697652316913	-0.423269996727	-0.319501025360
	O	-4.426497376104	-1.037096035004	-1.068495079288
	N	-2.462105224842	0.020112023822	-0.708110053038
	H	-1.986304185202	0.692367066923	-0.111966010564
	C	-1.969390193336	-0.280121002304	-2.027921150030
	H	-2.194488219490	-1.362700076923	-2.178090159566
	C	-2.675827236626	0.439147055719	-3.191327232863
	H	-2.562652230677	-0.177443989248	-4.103177300234
	H	-3.750336314806	0.379648061325	-2.949931214203
	C	-2.362027197052	1.892527155585	-3.539276255088
	H	-1.419130131145	2.001325156587	-4.116801296600
	H	-3.148934250767	2.207681185110	-4.242038305262
	C	-2.353033189401	2.888893227375	-2.390803173214
	H	-2.882094220494	3.814084302995	-2.680619195913
	H	-2.898973233744	2.481141206893	-1.533105110217
	N	-0.975035085053	3.247343242929	-1.993202148229
	H	-0.262326037212	3.129486230142	-2.711871197715
	C	-0.742052063884	4.257257310648	-1.135467085159
	N	-1.600888122555	4.538705342381	-0.181999015772
	H	-2.312144176673	3.816476295287	0.136157007183
	H	-1.472492105637	5.345358407012	0.429359028189
	N	0.405706027558	4.963022351750	-1.274745092441
	H	0.915259064252	4.921817346119	-2.151303159596
	H	0.557553045451	5.779138386202	-0.692576054894
	C	-0.468936082456	-0.193221009399	-1.992016145595
	O	0.193349964073	-0.311928024255	-0.961506070712
	N	0.179482966340	0.020347999833	-3.177588230406
	H	-0.352932070592	0.186052016602	-4.026999293015
	C	1.614064069836	0.170019997577	-3.160363230487
	H	2.044378091506	-0.702448068145	-2.635408191505
	C	2.180082109734	0.240053997639	-4.564640329938
	H	1.757270075170	-0.579014056330	-5.174740374584
	H	1.874657099183	1.187857066880	-5.046950366160
	C	3.686011221785	0.140354976649	-4.618116336836
	O	4.403388269141	0.036029962604	-3.605463263552
	N	4.231294260822	0.157841972753	-5.827797398438
	H	5.242409333350	0.103008959593	-5.921534427547
	H	3.672104221426	0.235412983516	-6.671613491161
	C	1.989988110831	1.446433084225	-2.384427174751

	O	1.854477110004	2.552247166600	-2.841629205744
	N	2.524064144129	1.174923059438	-1.110998082626
	H	1.898025095531	0.462547015960	-0.682980049912
	C	2.869352182682	2.297647138390	-0.206853017508
	H	2.280977144862	3.187795207563	-0.507726039012
	C	2.549946155751	1.880139110894	1.229453086872
	H	1.577789080544	1.348910082946	1.220881087493
	H	3.298524202806	1.125541053494	1.550269106583
	C	2.430611156199	3.002155193022	2.267878161121
	H	3.281323222243	3.711798235508	2.161062151170
	C	1.156298073481	3.808042263001	2.060231146489
	H	0.266434001037	3.162258224799	2.169122155685
	H	1.073433070329	4.610078319636	2.810429200899
	H	1.114163070585	4.277648298672	1.064364072969
	C	2.465766152427	2.419022153279	3.671065261275
	H	3.382865213600	1.835438101059	3.867405276834
	H	2.412373157565	3.216090209232	4.428731313736
	H	1.606192087289	1.746442108510	3.844376276723
	C	4.357531287482	2.562742143183	-0.506204038988
	O	4.784705319526	2.305730125073	-1.641016120190
	N	5.188101353981	2.869597159998	0.494836033188
	H	4.775896322825	3.032050172686	1.411298101481
	C	6.597876451589	2.483171120237	0.493914032850
	H	7.202479503515	3.221557165850	-0.062934007215
	C	7.054848470399	2.446100110776	1.980229140424
	H	6.708570489383	3.419248186801	2.382358171084
	C	6.391514416904	1.318885036806	2.762184195776
	H	6.780552459691	0.334184962697	2.446881171104
	H	6.612730456968	1.428000041439	3.834804275222
	H	5.293089347584	1.299381045104	2.660324190059
	C	8.558753595892	2.394315095070	2.176458155763
	H	9.087840620171	3.203681145621	1.648161113740
	H	8.787506601678	2.509150098298	3.246685232187
	H	8.974458626541	1.418033019705	1.874596132672
	C	6.720433426198	1.113481019321	-0.176663015177
	O	5.716632392892	0.350965973378	-0.177626015109
	N	7.849698522247	0.671855976831	-0.704703051156
	C	9.150340633778	1.337417014570	-0.869288066063
	H	9.272887659855	2.167183070324	-0.165277014174
	H	9.210581642627	1.741418041499	-1.896797139725
	C	10.119772663116	0.190806921839	-0.663322051756
	H	10.214359709640	-0.028732094827	0.413573027372
	H	11.123838761305	0.407839928465	-1.053298077091
	C	9.434056634532	-0.965330154226	-1.384406102161
	H	9.751524618581	-1.950200231289	-1.010723073897
	H	9.660238632654	-0.935893156304	-2.462209181713
	C	7.931300511600	-0.734783126438	-1.165927088539
	H	7.361016456391	-0.825416128808	-2.112002154874

	C	7.316823488467	-1.635685183292	-0.077839007819
	O	7.696702478510	-1.545865182389	1.066189073007
	N	6.325071397779	-2.462020233684	-0.526687040352
	H	6.210694392167	-2.497650235286	-1.534355111547
	C	5.186186298560	-2.965280259607	0.231580014058
	H	5.284543299058	-4.051033338308	0.425304028128
	C	4.966865289850	-2.226201203468	1.567349111935
	H	5.706245331424	-2.600954238713	2.293583162953
	H	5.214295315592	-1.166125133124	1.410693098424
	C	3.565321189802	-2.281802195694	2.161321151683
	H	2.847782140122	-1.750653152888	1.498254105152
	H	3.177158152180	-3.312616267032	2.265282160415
	C	3.571945197767	-1.589203148049	3.514342248632
	H	4.110541230288	-2.246709197873	4.227094302707
	H	4.189112251112	-0.664453083457	3.444768246478
	N	2.245293103679	-1.284292111485	3.970033282784
	H	1.453064045579	-1.395114114921	3.338609236540
	C	1.951178087344	-0.611203061703	5.130143369246
	N	2.991375164529	-0.515806064419	6.025360432579
	H	3.630441202303	-1.302918129338	6.084712423650
	H	2.741971148895	-0.146805035585	6.937072506400
	N	0.788601008298	-0.069443012264	5.266669374370
	H	-0.385443082967	-0.903144059147	4.323916308176
	H	0.629677999452	0.286275015145	6.208588425469
	C	3.949953212853	-2.756515236327	-0.624314047185
	O	3.789786208838	-1.710504157500	-1.303066097880
	N	3.016755135112	-3.685041292805	-0.591368043173
	H	3.070468134041	-4.468973349068	0.079392003342
	C	1.648238040237	-3.462537261775	-1.026667078574
	H	1.457197036313	-2.374601184045	-1.053066080494
	C	1.312373008039	-4.074902306758	-2.398571172431
	H	0.301554941533	-3.694635271038	-2.648699192503
	C	2.280987082811	-3.670406287430	-3.491248254971
	H	3.295074152355	-4.051920324046	-3.277310238326
	H	1.957098056730	-4.103774312715	-4.448723323280
	H	2.351653100427	-2.574773204902	-3.596735262720
	O	1.173749989896	-5.459493419868	-2.317561168375
	H	2.023535043234	-5.867214435855	-2.105828153950
	C	0.777310969356	-4.116532305221	0.055212001439
	O	1.283168001422	-4.926658365429	0.820949057893
	N	-0.484196115484	-3.702512259601	0.018992998888
	H	-0.780581131294	-3.046597212927	-0.712692055959
	C	-1.650388203819	-4.246147292515	0.684719048063
	H	-1.517363203623	-5.333539391909	0.837097060400
	C	-1.916737215814	-3.534201238891	2.012460141303
	H	-1.929477205710	-2.450241160880	1.793685127096
	H	-2.927412293335	-3.789392246960	2.377236169239
	C	-0.888557145400	-3.840991267447	3.089177222138

	H	-1.098159168336	-4.792990335499	3.600153255614
	H	0.114455925519	-3.962251286828	2.636792185806
	C	-0.714663124793	-2.780108191215	4.141897294796
	O	-0.512612110885	-3.004062210241	5.305399386257
	O	-0.704085109715	-1.546225102295	3.614764257508
	C	-2.814691284423	-3.965024260282	-0.289219023447
	O	-2.614223262980	-3.251953212868	-1.271407095272
	N	-3.979251375833	-4.485168284304	0.086617003729
	H	-4.028277383074	-5.024926326563	0.961657066148
	C	-5.315679448084	-4.040275244212	-0.273639022337
	H	-5.379715451575	-2.939434163245	-0.166961014405
	C	-5.726460484431	-4.338633261939	-1.715390125125
	H	-4.916329434082	-3.947285238750	-2.364944170218
	H	-5.772599501689	-5.432509348998	-1.876920136272
	O	-6.967755579158	-3.792898210280	-2.021630148016
	H	-7.037668572257	-2.868870144542	-1.670635123336
	C	-6.212352516159	-4.632795274506	0.820221058168
	O	-5.701939521985	-5.106978317665	1.832315129774
	N	-7.526644648993	-4.573067260666	0.599941042697
	H	-7.875977676103	-4.171243226605	-0.270428021887
	H	-8.151061684654	-4.905634280328	1.325820095158
	H	-8.760968680147	2.328553248775	-5.350616365076
	H	-9.090804700696	0.840886144131	-4.424834320180
	H	-9.617137727818	2.424523262997	-3.804782277142
	C	-8.797921656622	1.908320217743	-4.333823315918
	Zn	4.364676270448	0.148571971120	-1.619552119459
**(ZnA5)$^{2+}$ - 5**<br><br>**E** = -6799.213322<br>**H** = -6797.328455<br>**G** = -6797.605987	N	-11.407806282285	2.048952213443	-1.126949501277
	H	-10.564958213503	2.337347236475	-0.588426463466
	C	-11.748492288558	0.685289118595	-0.704191469251
	H	-12.712572772690	0.924371180876	-0.240481961420
	C	-12.080803543798	-0.485579738839	-1.540885459680
	H	-12.149627974295	-1.031755977871	-0.595002140080
	H	-12.647655553475	0.090603261261	-2.292251978284
	C	-11.297472282545	-0.412604980733	-2.729751381399
	O	-11.181249075708	-1.435348835349	-3.241932387565
	O	-10.481354776354	0.422487893017	-2.783069793524
	C	-10.780652236056	0.234050085448	0.371427606893
	O	-9.865295136427	0.978416139839	0.675262628727
	N	-10.954500244702	-1.006465003072	0.850427643655
	H	-11.716215281008	-1.583318047687	0.499154615707
	C	-9.910969165636	-1.623273046696	1.659658701090
	H	-9.725997110369	-0.998722002188	2.550694761438
	C	-10.334095166354	-3.031251148326	2.075769727108
	H	-11.271744228022	-2.946274145855	2.655911772371
	H	-10.570128169390	-3.617994192926	1.166593666460
	C	-9.274007083686	-3.736327200649	2.875615788989
	C	-8.602831046702	-4.845067278662	2.355073749591
	H	-8.892066048657	-5.241729305077	1.375056677701

	C	-7.594513961836	-5.472716343748	3.085972799690
	H	-7.090327955104	-6.351369391880	2.673938772161
	C	-7.246728936932	-4.993317285114	4.345989894438
	H	-6.464791921232	-5.490743348830	4.926347933979
	C	-7.916484027167	-3.890848211507	4.876812932309
	H	-7.743635530226	-3.365371524136	5.819941193881
	C	-8.922822094957	-3.267917163771	4.146137877647
	H	-9.452904131218	-2.410502104766	4.576810909038
	C	-8.582219063677	-1.628759048403	0.902655644221
	O	-7.539015968670	-1.333893023716	1.463895684528
	N	-8.647716072633	-1.992024074205	-0.395528449572
	H	-9.555190124620	-2.170047084966	-0.820268477942
	C	-7.506209995542	-1.853809062305	-1.278756510194
	H	-6.630847900721	-2.324996094383	-0.791130475748
	C	-7.767437974087	-2.513366107894	-2.629687611015
	H	-8.790910060352	-2.232987088289	-2.957413632535
	H	-7.095649943615	-2.048762077835	-3.374340660454
	C	-7.579700967693	-4.028098221068	-2.698283614236
	H	-6.504572926761	-4.226893232763	-2.513359602971
	C	-8.373663060117	-4.790762273269	-1.649527539558
	H	-9.457574120440	-4.581905257816	-1.739591544090
	H	-8.248846022396	-5.877256369455	-1.780165548185
	H	-8.068377000929	-4.540900254726	-0.620728464057
	C	-7.920390995714	-4.518942256450	-4.096342716967
	H	-7.348939945918	-3.984971217210	-4.872199771832
	H	-7.711760007739	-5.593974346963	-4.207330723306
	H	-8.994331086640	-4.370491242019	-4.312244732638
	C	-7.234982948289	-0.364216954765	-1.495468526897
	O	-8.142083030714	0.396894099267	-1.816719551473
	N	-5.958346856824	0.017417073956	-1.335250515487
	H	-5.215677809310	-0.678146975088	-1.232683507798
	C	-5.470114856218	1.351947172521	-1.614374538328
	H	-5.810850869742	1.638764188997	-2.631131608725
	C	-6.030333854315	2.417400244643	-0.660188465375
	H	-5.932232859187	3.403468315497	-1.150252504075
	H	-7.117756958204	2.233504234269	-0.624277465362
	C	-5.488905834495	2.531197256115	0.762109632997
	H	-4.497976773182	3.023147293114	0.767698633764
	H	-6.151659856808	3.221660306253	1.308045671468
	C	-5.379888812321	1.241075161835	1.559792693457
	H	-5.406711801636	1.466649177632	2.641419771855
	H	-6.237638882858	0.579573114684	1.349618677541
	N	-4.125234743779	0.586606118453	1.229110666657
	H	-3.326742677141	1.193061158457	0.980442651908
	C	-3.803377710708	-0.694445973031	1.377016681439
	N	-4.724668792337	-1.631902043756	1.613202696076
	H	-5.733122828275	-1.438784029713	1.639278698787
	H	-4.426737767732	-2.567781113361	1.860518715872

	N	-2.511229618997	-1.028055995759	1.285915674710
	H	-1.852754571545	-0.283991944411	1.030263652714
	H	-2.239439595943	-1.992022068624	1.138274661312
	C	-3.946056703128	1.227090160750	-1.699939541391
	O	-3.409039682601	0.132258084299	-1.742007545154
	N	-3.247798671025	2.380068244964	-1.752744546245
	H	-3.728667725846	3.271498309524	-1.695613543839
	C	-1.806960567270	2.361498245815	-1.902193557216
	H	-1.539974547577	1.819261209232	-2.827456622109
	C	-1.264456529015	3.784483348550	-2.001317563094
	H	-1.927928581353	4.382727393895	-2.653557611360
	H	-1.248245527957	4.264167385008	-1.006338491333
	C	0.126797571257	3.839920354038	-2.617393606413
	O	0.625354608464	2.876829286339	-3.176332646456
	N	0.717940608264	5.049320443688	-2.570589603857
	H	1.673997679458	5.107224447052	-2.907214629836
	H	0.398682586918	5.763199513009	-1.927374557153
	C	-1.136456521891	1.650451195543	-0.728002471625
	O	-1.672410557619	1.569822188523	0.382520607744
	N	0.091004567187	1.190555165509	-0.980171491303
	H	0.476455597867	1.445594183289	-1.897575554367
	C	1.058446636063	1.029017153202	0.087242586289
	H	0.681217612222	0.296374099993	0.825191641035
	C	2.379045736188	0.541478120788	-0.522945456829
	H	2.118801716170	-0.222423936296	-1.281943511927
	H	2.831105767729	1.391346180870	-1.070178498153
	C	3.410730806819	-0.068779924119	0.423398610411
	H	3.704334828436	0.703989131302	1.157453664098
	C	2.877448772233	-1.299173011187	1.145154664063
	H	2.454597742597	-2.020131064489	0.420661610427
	H	3.684412829041	-1.831382048941	1.679084702649
	H	2.085167714530	-1.064967998040	1.880701716069
	C	4.669432901396	-0.407790947237	-0.360543445715
	H	5.126466934488	0.489094117711	-0.813752480345
	H	5.414540953309	-0.880470979897	0.301181601492
	H	4.453808883701	-1.129316999289	-1.169008503735
	C	1.252660651332	2.391317251501	0.766338637651
	O	0.965433632558	3.433265323285	0.197928594242
	N	1.811190691867	2.355731250988	1.996460726276
	H	2.155083717061	1.455012183627	2.318424748403
	C	2.512504741104	3.520290331670	2.506645761475
	H	1.783547687899	4.341017394415	2.625222767536
	C	3.117590783690	3.182842308065	3.887650857332
	H	2.254852723329	2.831104281485	4.488625901790
	C	4.154877862265	2.067535230352	3.836460853816
	H	5.036865923006	2.372401255100	3.248884815432
	H	4.498127885771	1.831295213176	4.855787931732
	H	3.782444835332	1.121295161259	3.405395823794

	C	3.715273827066	4.397635396521	4.579949909103
	H	2.977601773855	5.204566453836	4.720480918151
	H	4.079440851364	4.118120375193	5.580563972426
	H	4.582932889760	4.790535427788	4.020151871507
	C	3.587235819724	3.907384362456	1.483072687024
	O	4.146312858609	3.030152298115	0.836545641630
	N	3.943965845174	5.202304453934	1.342411675001
	C	3.368326801961	6.410091553119	1.923131719406
	H	2.954721768891	6.228887504453	2.923461789175
	H	2.546517741078	6.788186580821	1.281615670345
	C	4.548800883115	7.365607596669	1.934924720155
	H	5.195061932273	7.137078591612	2.798316783270
	H	4.247022861620	8.421557672003	1.997469724412
	C	5.276128939302	7.017414576106	0.642253626164
	H	6.332474013702	7.326030588969	0.640275626167
	H	4.789442903571	7.508067615543	-0.217983435624
	C	5.120786925818	5.497554491967	0.520888617488
	H	4.943835917036	5.180565455662	-0.522710459796
	C	6.319754016123	4.740661424589	1.096498657554
	O	6.625240035519	4.858551431578	2.270592744956
	N	6.993413063886	3.977754368903	0.207033594810
	H	6.518123029549	3.722696353660	-0.659162470066
	C	7.952520146582	2.979853296791	0.631011625404
	H	8.771922170663	3.470148337236	1.186768665497
	C	7.276341109133	1.906908223138	1.507716688473
	H	7.148118074965	2.331506252085	2.518826760276
	H	6.257962009924	1.766702208297	1.097375657717
	C	7.941625127873	0.542146122396	1.588475693176
	H	7.935089153835	0.076362090045	0.581275621577
	H	9.005026230543	0.607339129607	1.888837715729
	C	7.174936090014	-0.361918941706	2.544159761086
	H	7.543195088518	-0.223148931353	3.574814835828
	H	6.104494003582	-0.049286920360	2.554577763547
	N	7.283477064335	-1.767412044437	2.173668735281
	H	7.041148069144	-1.906849053608	1.192127664665
	C	6.506561035835	-2.672445107610	2.909693787805
	N	6.806387054752	-2.693110108904	4.244901884243
	H	7.745926120591	-2.420681088447	4.540096909009
	H	6.294234019948	-3.330666155274	4.840061926914
	N	5.606579969650	-3.352255158254	2.296496743893
	H	9.187396215442	-4.407706230026	3.607816837376
	H	5.194585942629	-4.053027210924	2.917881789713
	C	8.476079173647	2.300683248987	-0.621846462122
	O	7.725194120165	2.027505231052	-1.542425530471
	N	9.786023268696	1.952543225526	-0.578875459717
	H	10.295307316128	2.083342236331	0.299733601615
	C	10.307774308772	0.834601146744	-1.336121514780
	H	9.456584236920	0.236050102923	-1.714260543570

	C	11.176031352342	1.254692178352	-2.531315603050
	H	11.497592416577	0.324349111355	-3.041751637602
	C	10.434280321519	2.135833243281	-3.508823670973
	H	10.138417268686	3.081787307947	-3.024397640145
	H	11.084867341770	2.375718259807	-4.362531732221
	H	9.516343271130	1.653345207709	-3.876992698761
	O	12.368642457666	1.850604224229	-2.083646567903
	H	12.146955449850	2.648118281351	-1.583207532976
	C	11.139092346146	0.023433089277	-0.341926444513
	O	11.530327374118	0.531179124998	0.697871631968
	N	11.389122404925	-1.252610001242	-0.690046471425
	H	11.142235341499	-1.618050030206	-1.611961537699
	C	12.280008443522	-2.107587061284	0.061483584564
	H	13.033191502126	-1.468082018064	0.561105620619
	C	11.546937401280	-2.947778125806	1.112044657438
	H	10.856903339262	-3.638517173631	0.595045622708
	H	12.279820426846	-3.584129166787	1.639103699871
	C	10.781825364541	-2.088707064810	2.099897733113
	H	11.419331387785	-1.326770006897	2.575284765640
	H	9.994821265011	-1.518833021426	1.570958693251
	C	10.029191274416	-2.792606115805	3.180562809607
	O	9.679699282460	-2.266380077739	4.212061885018
	O	9.728589280564	-4.058377203495	2.881313789686
	C	12.970121511704	-3.013185127005	-0.953476488797
	O	12.496003462095	-3.185379139241	-2.066875570313
	N	14.090141563707	-3.615566168955	-0.516243458721
	H	14.400945618660	-3.552806165273	0.455730612706
	C	14.794635605725	-4.595704242514	-1.296754511071
	H	14.069535560384	-5.308977292758	-1.741137546006
	C	15.571470664814	-3.963072195738	-2.456963596013
	H	14.921062665226	-3.220206140826	-2.951652633286
	H	16.447832745882	-3.417394152343	-2.065258568685
	O	16.039523708136	-4.938018262594	-3.355965663121
	H	15.318583683903	-5.182610284291	-3.948308704792
	C	15.703321685282	-5.361396289268	-0.331002443825
	O	15.653843718336	-5.153438277167	0.867764643939
	N	16.526030749663	-6.251610378595	-0.918175487274
	H	16.626309785431	-6.251676366998	-1.932097559353
	H	17.198623817484	-6.731472382653	-0.329997443623
	H	-12.758878350632	3.521903322134	-0.467815453522
	H	-11.991822328352	3.836750344187	-2.063793567930
	H	-13.276195414539	2.594645253721	-1.916968556432
	C	-12.424647354278	3.068751285714	-1.410022519922
	Zn	14.335512587232	-0.145277919952	-1.835508550627
